# Natural Extracts of *Alnus japonica* Induce BAK-Dependent Autophagy to Inhibit Liver Cancer Stem Cell Tumorigenesis

**DOI:** 10.3390/antiox15060685

**Published:** 2026-05-29

**Authors:** Kenly Wuputra, Yoshimasa Matsuura, Satoshi Gushiken, Hirosuke Fukuda, Ya-Han Yang, Chia-Chen Ku, Chun-Chieh Wu, Ying-Chu Lin, Yi-Chun Tsai, Deng-Chyang Wu, Toshihiko Nozaki, Kohsuke Kato, Atsushi Kawaguchi, Kyosuke Nagata, Yoshiharu Tanaka, Kazunari K. Yokoyama

**Affiliations:** 1 Cell Therapy Research Center, Department of Medicine, Kaohsiung Medical University Hospital, Kaohsiung 80756, Taiwan; kenlywu@hotmail.com (K.W.); r991046@gap.kmu.edu.tw (C.-C.K.); 2 Graduate Institute of Medicine, Kaohsiung Medical University, Kaohsiung 80708, Taiwan; 3 Regenerative Medicine and Cell Research Center, Kaohsiung Medical University, Kaohsiung 80708, Taiwan; dechwu@kmu.edu.tw; 4 Division of Nephrology, Department of Internal Medicine, Kaohsiung Medical University Hospital, Kaohsiung 80756, Taiwan; sc9522059@yahoo.com.tw (Y.-H.Y.); 920254@kmuh.org.tw (Y.-C.T.); 5 Faculty of Liberal Arts and Sciences, Osaka Prefecture University, Sakai City 599-8531, Osaka, Japan; ynda1956@yahoo.co.jp; 6 Okinawa Eco-Science Co., Ltd., Nago City 905-2261, Okinawa, Japan; e-science@outlook.jp (S.G.); hukuda@leaf.ocn.ne.jp (H.F.); 7 Division of Gastroenterology, Department of Internal Medicine, Kaohsiung Medical University Hospital, Kaohsiung 80756, Taiwan; 8 Forensic Pathology Division, Department of Pathology, Kaohsiung Medical University Hospital, Kaohsiung 80756, Taiwan; lazzz_wu@yahoo.com.tw; 9 Department of Medicine, Kaohsiung Medical University, Kaohsiung 80708, Taiwan; 10 School of Dentistry, Kaohsiung of Medical University, Kaohsiung 80756, Taiwan; chulin@cc.kmu.edu.tw; 11 Division of Nephrology, Department of Internal Medicine, Kaohsiung Medical University CiJin Hospital, Kaohsiung 805, Taiwan; 12 Ryukyu Fertilizer Co., Ltd., Okinawa City 904-2162, Okinawa, Japan; t-nozaki@ryuhi.co.jp; 13 Department of Infection Biology, Institute of Medicine, University of Tsukuba, Tsukuba 305-8575, Ibaraki, Japan; kkato@md.tsukuba.ac.jp (K.K.); ats-kawaguchi@md.tsukuba.ac.jp (A.K.); knagata@md.tsukuba.ac.jp (K.N.); 14 Radiation Biology and Molecular Genetics, Division of Quantum Radiation, Faculty of Technology, Osaka Metropolitan University, Sakai City 599-8531, Osaka, Japan; 15 Research Institute for Biomedical Sciences, Tokyo University of Science, Noda 278-0022, Chiba, Japan

**Keywords:** BAK, autophagy, cancer stem cells, *Alnus japonica*, natural extracts, liver tumorigenesis

## Abstract

**Background:** Cancer stem cells (CSCs) contribute to hepatocellular carcinoma (HCC) progression and therapeutic resistance. Natural products with antioxidant and bioactive properties may offer novel strategies to suppress CSC-driven tumorigenesis. **Methods:** We investigated the effects of unfermented and fermented *Alnus japonica* bark extracts on CSC-like rG2-DC-1C cells. Cell proliferation, invasion, and xenograft tumor formation were assessed, and autophagy/apoptosis markers were analyzed. **Results:** Bark extracts reduced OCT4 expression, suppressed CSC proliferation and invasion, and inhibited xenograft tumor formation. Mechanistically, extracts activated BAK-dependent autophagy, evidenced by LC3B accumulation and p62 modulation, whereas diarylheptanoids Hirsutenone (Hir) and Oregonin (Ore) primarily induced apoptosis via Caspase-3 cleavage. Blocking autophagy with chloroquine or BAK knockdown reversed the anti-invasive effects of bark extracts, confirming BAK’s role in CSC suppression. Component analysis suggests quercetin contributes to autophagy induction, though synergistic effects of other constituents remain possible. **Conclusions:** Together, these findings indicate that *Alnus japonica* bark extracts suppress CSC-driven liver tumorigenesis through autophagy, while Hir and Ore act via apoptosis, highlighting complementary mechanisms that broaden the therapeutic potential of this traditional medicinal plant and support further preclinical validation.

## 1. Introduction

*Alnus japonica* (*A. japonica*) is an alder species whose roots form symbiotic associations with *Frankia* actinomycetes, enabling nitrogen fixation [1]. Traditionally, *A. japonica* has been used in East Asia to treat fever, hemorrhage, diarrhea, alcoholism, skin infections, and inflammatory conditions [2,3,4]. Fermented extracts prepared by cultivating alder chips with *Frankia* exhibit diverse biological activities, including antibacterial, antiviral, and anthelmintic effects in both *in vitro* and *in vivo* models [5,6,7], and have been patented for use in fermented extracts and nutrient drinks [8]. Clinical and animal studies further suggest metabolic and protective benefits, including improved lipid profiles, reduced organ fat, and alleviation of allergic and inflammatory symptoms [9,10,11]. Extracts have also demonstrated growth inhibition of prostate cancer cells, suppression of allergic responses, and efficacy against viral infections, including influenza and coronavirus [11].

These properties have been patented for use in fermented extracts and nutrient drinks [8]. Clinical and animal studies further suggest metabolic and protective benefits, such as improved lipid profiles, reduced organ fat, and alleviation of allergic and inflammatory symptoms [9,10,11].

Phytochemical investigations of *A. japonica* bark have identified bioactive compounds including diarylheptanoids, triterpenoids, flavonoids, and lignans. Among them, Hirsutenone (Hir) and Oregonin (Ore) are major diarylheptanoids with potent antioxidant activity [12,13]. These compounds, together with other constituents, display antioxidative, anti-inflammatory, hepatoprotective, anticancer, and anti-obesity effects [12,13,14,15,16,17,18,19]. Despite these findings, the comparative roles of whole bark extracts versus purified components in regulating cancer stem cell biology remain unclear, particularly in the balance between autophagy and apoptosis. For example, lignan derivatives such as (2R,3R)-1,4-O-4-O-diferido secoisolariciresinol exhibit anticancer activity in xenograft models by downregulating the FOX1–Wnt/β-catenin pathway [13,20]. Hir and Ore have been shown to induce apoptosis in diverse cancer cell lines, including melanoma, gastric, leukemia, colon, ovarian, thyroid, and prostate cancers, through caspase activation, PARP cleavage, and ROS-mediated pathways [16,17,18,19,20,21,22,23,24,25]. Ore additionally demonstrates hepatoprotective and anti-inflammatory effects, including suppression of pro-inflammatory mediators via AMPK–HO-1 signaling [26,27]. Other bark-derived triterpenes and steroids exhibit anti-influenza activity [28], and some components are expected to be effective against COVID-19 [29]. Despite these findings, no clinical trials of fermented alder water in humans have been reported. To investigate cancer stem cell (CSC) biology, HepG2-derived CSC-like cells (rG2-DC-1C) were generated by lentiviral transduction of OCT4, SOX2, KLF4, c-Myc, and shRNA against TP53 [30]. This clone exhibits enhanced tumor sphere formation, colony growth, chemoresistance, invasion, and stemness marker expression compared to parental HepG2 cells. We previously identified an OCT4–c-Jun positive feedback loop as critical for maintaining CSC-like characteristics in rG2-DC-1C cells [30].

In this study, we compared unfermented and fermented *A. japonica* bark extracts with purified Hir and Ore to evaluate their effects on CSC-like HepG2 cells. We found that whole bark extracts suppress CSC tumorigenicity primarily via BAK-mediated autophagy, whereas Hir and Ore induce apoptosis. These findings suggest that flavonoid-enriched bark extracts, including quercetin, inhibit CSC-driven tumorigenesis through autophagy, providing a distinct and complementary mechanism to apoptosis.

## 2. Materials and Methods

### 2.1. Animals and Reagents

Mouse embryonic fibroblasts (MEFs), HepG2, SNL76/7, 293, and 293T cells were obtained from the American Type Culture Collection (Manassas, VA, USA) and the RIKEN Cell Bank (Tsukuba, Ibaraki, Japan). Cells were cultured in Dulbecco’s Modified Eagle Medium (DMEM; Gibco, Grand Island, NY, USA) with or without high glucose. All media were supplemented with 10% fetal bovine serum (FBS; Gibco) and 1% penicillin–streptomycin (P/S; Gibco). The study was conducted in accordance with the Declaration of Helsinki. Animal experiments were approved by the National Laboratory Animal Center (Taiwan), and Kaohsiung Medical University (Taiwan). All procedures complied with the animal welfare guidelines of the National Laboratory Animal Center (protocol no. 106022; 28 April 2018), and Kaohsiung Medical University (protocol nos. 106189; 5 May 2018, 107128; 26 March 2019, 108244; 15 March 2020; 11036; 27 April 2021, and 111035; 20 June 2022).

Experiments were conducted in accordance with these guidelines approved. Hirstenone (Hir) and Oregonin (Ore) were purchased from Sigma-Aldrich, Inc. (SMB00096 and SMB00088; St. Louis, MO, USA; Merck KGaA, Darmstadt, Germany). Apigenin (cat. no. 010-18914) was obtained from Fujifilm Wako Pure Chemicals (Osaka, Japan). Luteolin (cat. no. 491-70-3) was purchased from Sigma-Aldrich, Inc. Kaempferol (cat. no. 520-18-3) and quercetin (cat. no. 849061-97-8) were obtained from Tokyo Kasei Co. (Tokyo, Japan). Chloroquine diphosphate (C6628) was purchased from Sigma-Aldrich, Inc. (St. Louis, MO, USA; Merck KGaA, Darmstadt, Germany). Torin 1 (CAS 1222998-36-8) was obtained from NJ, USA, and Bafilomycin A (CAS 88899-55-2) was purchased from AdipoGen Life Sciences (San Diego, CA, USA).

### 2.2. Generation of Unfermented and Fermented Tree Bark Extracts of A. japonica

*Alnus japonica* has been cultivated in the Yanbaru district of Okinawa since its introduction from Taiwan (*Alnus formosana*) in 1910. Gushiken et al. reported the preparation of fermented bark extracts from *A. japonica* roots coexisting with the symbiotic fungus *Frankia*, yielding fermented tree extracts that were developed as agricultural antibacterial materials [31]. A voucher specimen of *A. japonica* stem bark was deposited at the University of the Ryukyu, Okinawa, Japan [32]. Stems, leaves, and rhizomes of *A. japonica* were gently washed to remove *Frankia* fungi, then mixed and cut into small pieces using an electric cutter (Hitachi Inc., Ibaraki, Japan). The pieces were fermented at room temperature for 7–10 days, dried, and ground into powder. During fermentation, the materials were stirred occasionally to ensure uniform processing, allowing the surfaces to be covered with white mycelium and promoting bacterial growth. For preparation of unfermented extracts, the fermentation step was omitted. For extraction, water was added at a ratio of 100 L per 1 m^3^ of plant material, followed by boiling and distillation.

### 2.3. Isolation of Polyphenols, Flavonoids, and Other Secondary Metabolites

Boiled extracts of fermented and unfermented *A. japonica* bark were prepared with 50% aqueous EtOH under sonication. The concentrated residue was partitioned with CH_2_Cl_2_ and EtOAc, yielding soluble fractions subsequently fractionated by silica gel and RP-C18 chromatography to isolate diarylheptanoids, triterpenoids, and flavonoids, including hirsutanone, oregonin, and related compounds [2,3,4,19]. Lignans such as (−)—(2R,3R)-1,4-O-diferuloylsecoisolariciresinol (DFS) were obtained by methanol extraction and purified by silica gel and RP chromatography, with purity confirmed by HPLC (Shimadzu Corp. Kyoto, Japan) [13]. Major flavonoids (quercetin, kaempferol, myricetin, apigenin, luteolin) were extracted by refluxing bark material in acidified aqueous methanol, followed by filtration and reversed-phase liquid chromatography (RP-HPLC; Shimadzu Corp., Kyoto, Japan), quantification against authentic standards [33]. Structural confirmation was achieved by LC-MS and NMR, consistent with reported data [34]. In general, NMR spectra were recorded at 100 and 270 MHz (^1^HNMR), and 25.05 MHz (^13^CNMR). Chemical shifts are given in 6 (ppm) with TMS as int. std. Negative FAB-MS were measured at 1.5 kV (accelerating voltage) with Me and O-glycerol as the matrix. In some cases, high-resolution analytical methods (HPLC, UHPLC-QqQ-MS/MS (Agilent Technologies, Wilmington, DE, USA) coupled with a hybrid quadrupole linear ion trap mass spectrometer QTRAP^®^ 5500 (AB Sciex, Foster City, CA, USA) equipped with an electrospray ionization source (ESI), LC-ESI-MS/MS (Agilent Technologies, Wilmington, DE, USA) were also employed for separation, identification, and quantification of phenolic compounds, with QTOF-MS (Time-of-Flight Mass Spectrometry, Shimadzu Corp. Kyoto, Japan), providing accurate molecular mass determination [35].

### 2.4. Generation of rG2-DC-1C Cells

Recombinant lentiviruses encoding human OSKM (RDB08323, RDB08324, RDB12904 in RIKEN BRC) C-JUN (RDB06254 in RIKEN BRC), and *shBAK* (TRCN000033464; siRNA Core Facility at Academia Sinica (Taipei, Taiwan) were produced in 293T cells co-transfected with *pCAG-HIVgp* and *pCMV-VSV-G-RSV-Rev* by the Lentivirus preparation protocol in RIKEN BioResorce Center, Japan as described elsewhere [30]. HepG2 cells and the iPSHep FB/Ng/gfp-103C-1 cell line were infected with lentiviruses at a multiplicity of infection (MOI) of 50 and incubated for 1 week in DMEM [30]. Subsequently, cells were transferred onto mitomycin C (Sigma-Aldrich)-treated MEFs in DMEM supplemented with ESGRO (Merck KGaA, Darmstadt, Germany; 10 ng/mL) to generate iPSC-like cells from HepG2 cells. A single colony (approximately 200 cells) of HepG2-derived iPSC-like cells was injected into severe combined immunodeficiency (SCID) mice. Tumor formation was observed approximately 9 weeks later. The tumor was resected, and a primary cancer cell line was established, designated “reprogrammed HepG2-derived cancer cells from one colony” (rG2-DC-1C). This cell line was used for subsequent experiments.

### 2.5. Cell Viability Assays Using MTT and Trypan Blue Dye Exclusion

Cell viability was assessed using the 3-[4,5-dimethylthiazol-2-yl]-2,5 diphenyl tetrazolium bromide (MTT) assay as described previously [36]. Cells (3 × 10^4^) were treated with unfermented or fermented tree bark extracts, as well as the indicated concentrations of Hir and Ore, at 37 °C for indicated time periods. Following treatment, 10 μL of MTT solution (10 mg/mL) was added to each well and incubated for 2 h at 37 °C. After centrifugation at 412× *g* for 5 min, the medium was removed, and 100 μL of dimethyl sulfoxide (DMSO; Sigma) was added to dissolve the formazan crystals. Absorbance was measured at 570 nm using a microplate reader (Immuno Mini NJ-2300, Nihon InterMed, Tokyo, Japan) during the culture periods until 8 days. Cell viability was expressed relative to the absorbance of untreated control cultures. Cell viability was also determined using the trypan blue dye exclusion assay as described elsewhere [37]. Briefly, cells were stained with trypan blue and counted using a hemocytometer to determine the number of viable cells in suspension. Proliferation rates were calculated based on viable cell counts.

### 2.6. Nuclear Staining Using Hoechst 33258

Cells were exposed to tree bark extracts or chemical treatments for 24 h, harvested, and washed with phosphate-buffered saline (PBS). They were then fixed with 1% glutaraldehyde for 30 min, followed by PBS washing. Nuclear staining was performed using Hoechst 33258 for 10 min. After washing with PBS, nuclear morphology was examined using fluorescence microscopy (Eclipse E600, Nikon, Tokyo, Japan).

### 2.7. Cell Proliferation Assay and Apoptosis

For the cell proliferation assay, cells were plated and cultured for 3~8 days under environmental oxygen conditions (20%), followed by treatment with 10 μM 5-ethynyl-2-deoxyuridine (EdU) for 6 h. Growing cells were stained with Alexa Fluor azide, and total cells were counterstained with Hoechst using the Click-iT EdU Assay Kit (C10337, Thermo Fisher Scientific, Waltham, MA, USA). Fluorescence microscopy was used to image stained cells. The percentage of proliferating cells was calculated by dividing the number of EdU-positive cells by the total number of Hoechst-stained cells. Cell counts were obtained from five randomly selected fields using Cell Count software (version 1.1.7). For apoptosis analysis, cells were incubated for 24 h at 37 °C. Caspase-3 and -7 activities, key indicators of apoptotic pathways, were measured using the Caspase-Glo 3/7 Assay Kit (Promega). Cell viability was additionally assessed using the trypan blue dye exclusion assay, in which viable cells were quantified by hemocytometer counting.

### 2.8. Sodium Dodecyl Sulfate Polyacrylamide Gel Electrophoresis (SDS-PAGE), Immunohistochemistry, and Western Blotting

Cell lysates were prepared from nuclear and cytoplasmic fractions using NE-PER nuclear and cytoplasmic extraction reagents (78833; Thermo Fisher Scientific). Lysates were separated by 10% sodium dodecyl sulfate polyacrylamide gel electrophoresis (SDS-PAGE) and transferred to Immobilon-P polyvinylidene difluoride (PVDF) membranes (0.45 μm; IPVH00010; Merck) using a Mini Trans-Blot transfer system (Bio-Rad Laboratories, Hercules, CA, USA). Protein transfer was confirmed by Ponceau S staining (P17170; Merck). Membranes were incubated with the indicated primary antibodies, followed by appropriate secondary antibodies. Signals were detected using a ChemiDoc XRS Plus imaging system (Bio-Rad). Immunoprecipitation was performed using protein A/G beads coated with the relevant antibodies.

Primary antibodies included: AHR (SC-8088; Santa Cruz, Dallas, TX, USA), OCT4 (2750S; Cell Signaling Technology, Boston, MA, USA), ABCG2 (GTX100437; GeneTex, Irvine, CA, USA), HO-1 (SC-10789; Santa Cruz), NQO1 (SC-16464; Santa Cruz), β-actin (SC-81178; Santa Cruz), ARNT (5537S; Cell Signaling Technology), ATG5 (GTX113309; GeneTex), BECLIN 1 (GTX133555; GeneTex), p62 (GTX100685; GeneTex), RIPK1 (GTX22563; GeneTex), BAK (SC-832; Santa Cruz), C-JUN (9165S; Cell Signaling Technology), GAPDH (MAB374; Millipore, Billerica, MA, USA), Caspase-8 (4790S; Cell Signaling Technology), Caspase-3 (9662S; Cell Signaling Technology), cleaved Caspase-3 (9664S; Cell Signaling Technology), and LC3B (GTX116080; GeneTex). Fluorescently labeled secondary antibodies (A11034 and A11029) were obtained from Invitrogen (Thermo Fisher Scientific, Waltham, MA, USA).

### 2.9. Nuclei Isolation and G-Banding

Nuclei were stained with 0.5 μg/mL 4′,6-diamidino-2-phenylindole (DAPI; D3571; Invitrogen, Thermo Fisher Scientific) for 1 h. Metaphase mitotic chromosomes were prepared using a conventional air-drying technique, and GTG (G-banding) staining was performed as described previously [38].

### 2.10. Isolation of RNA and Quantitative PCR (qPCR)

Total RNA was extracted using the PureLink™ RNA Mini Kit (Invitrogen). RNA was reverse transcribed into cDNA using SuperScript III Reverse Transcriptase (Invitrogen) [39]. Quantitative real-time PCR was performed on a StepOne or ABI7500 instrument (Applied Biosystems, Foster City, CA, USA) using Fast SYBR^®^ Green Master Mix (Applied Biosystems) in 20 μL reaction volumes. Threshold cycle (Ct) values were averaged from technical duplicates. Transcript levels of target genes were normalized to Gapdh expression. Relative gene expression was calculated using the 2^−ΔΔCt^ method, with expression levels normalized to DMSO-treated rG2-DC-1C cells (set as 1.0). Data are presented as mean ± SEM from three biological replicates.

The primer sequences used were as follows:

• OCT4: forward 5′-GGGTTTTTGGGATTAAGTTCTTCA-3′, reverse 5′-GCCCCCACCCTTTGTGTT-3′
• SOX2: forward 5′-GCTACAGCATGATGCAGGACCA-3′, reverse 5′-TCTGCGAGCTGGTCATGGAGTT-3′
• KLF4: forward 5′-CATCTCAAGGCACACCTGCGAA-3′, reverse 5′-TCGGTCGCATTTTTGGCACTGG-3′
• C-JUN: forward 5′-CCTTGAAAGCTCAGAACTCGGAG-3′, reverse 5′-TGTCTGCGTTAGCATGAGTTGGC-3′

### 2.11. Construction of AhR or c-Jun Promoter Luciferases and Their Corresponding cis-Element Mutant Luciferases

*AhR* promoter regions [39,40] were cloned into the *pGL4.1* plasmid (Promega, Madison, WI, USA) as described previously [40]. The orientation and integrity of the constructs were confirmed by restriction enzyme digestion and next-generation sequencing. Putative binding sites within the *AhR* promoter region were predicted using ALGGEN-PROMO (http://alggen.lsi.upc.es/cgi-bin/promo_v3/promo/promoinit.cgi?dirDB=TF_8.3, accessed on 17 May 2026). Site-directed mutagenesis of individual promoter sites, including DRE2, was performed using the QuickChange Lightning Site-Directed Mutagenesis Kit (Agilent Technologies, Santa Clara, CA, USA) [39,40].

Wild type and mutant M4 *c-JUN* promoter were generated and transfected into rG2-DC-1C cells as described previously [30]. Two days after transfection, cells were harvested and measured for luciferase. The WT *c-JUN* promoter and M1 to M4 mutants were co-transfected with various amounts (0–200 ng) of OCT4-expressing plasmids into rG2-DC-1C cells.

### 2.12. Transient Transfection and Luciferase Reporter Assay

rG2-DC-1C cells were seeded into 24-well plates (4 × 10^4^ cells/well) and cultured for 24 h. Cells were co-transfected with 500 ng of the *AhR* luciferase plasmid and 10 ng of the pRL-CMV plasmid encoding *Renilla* luciferase using either Lipofectamine 2000 (Invitrogen) or polyethylenimine (linear, molecular weight 25,000; Polysciences, Warrington, PA, USA; Cat# 23,966). The total amount of transfected DNA was maintained at 1 μg/well by supplementing with pBluescript II SK+ control plasmid (Addgene, Watertown, MA, USA). The human OCT 4 expression vector (*pCEP4_WT_OCT4*) was a gift from James Thomson (Addgene Plasmid #40629; http://n2t.net/addgene:40629 (accessed on 5 April 2013); RRID: Addgene_40629) and used for co-transfecting rG2-DC-1C cells.

Transfected cells were treated with DMSO for the indicated times and harvested 48 h post-transfection. Luciferase activity was measured using the Dual-Luciferase Reporter Assay System (Promega) according to the manufacturer’s instructions, with detection performed using a GloMax 20/20 Luminometer (Promega). Reporter activity was calculated as the ratio of Firefly luciferase to Renilla luciferase and expressed as fold induction relative to the empty vector in rG2-DC-1C cells [30]. All measurements were performed in duplicate, and values are presented as mean ± standard error of the mean (SEM) from at least three independent experiments.

### 2.13. shRNA Lentivirus and Autophagosome Inhibitor

shRNA lentiviruses targeting human BAK1 (TRCN0000033464) were obtained from the siRNA Core Facility at Academia Sinica (Taipei, Taiwan). Predesigned ON-TARGETplus SMARTpool siRNA against human BAK1 and scrambled control siRNA were purchased from GE Dharmacon (Austin, TX, USA). Mouse embryonic fibroblasts (MEFs) were seeded into six-well plates (for Western blotting) or 24-well plates (for luciferase reporter assays) and transfected with 20–40 nM of either siRNA or control RNA in OPTI-MEM medium (0.5 mL for 24-well plates; 2 mL for six-well plates) using Lipofectamine RNAiMAX (Invitrogen). For rG2-DC-1C cells, shRNA was transduced at a multiplicity of infection (MOI) of 10. After 24 h, fresh culture medium containing 10% FBS was added, and cells were subsequently transfected with luciferase plasmids for reporter assays as described above. Knockdown efficiency was confirmed 48 h post-infection by immunoblotting and complementary analyses. To inhibit autophagosome formation, 10 μM chloroquine was added during rG2-DC-1C cell culture and invasion assays involving *A. japonica* bark extracts. Following treatment, protein levels of BAK, Bax, Bad, Bcl-2, and p62 were examined by Western blotting.

### 2.14. ROS Detection Using CM-H2DCFDA Fluorescence

Reactive oxygen species (ROS) levels were measured as described previously [40]. rG2-DC-1C cells were cultured in 0.1% gelatin-coated 12-well plates with or without tree bark extracts (unfermented or fermented), or with 20 μM Hir or Ore for the indicated times. As positive controls, cells were treated with 150–200 μM hydrogen peroxide for 15 min or with 50 μM apigenin, luteolin, kaempferol, or quercetin for the indicated times before CM-H2DCFDA addition. Cells were rinsed with warm Hanks’ balanced salt solution (HBSS; Gibco Invitrogen, Waltham, MA, USA) and incubated with 10 μM CM-H2DCFDA (C-6827; Life Technologies, Carlsbad, CA, USA) in complete growth medium for 30 min at 37 °C in the dark. After treatment, cells were washed twice with HBSS and examined using a Nikon inverted fluorescence microscope. Five randomly selected fields were imaged using a 10× objective lens, and fluorescence intensity was quantified with ImageJ software version 1.54p run with Java 21.0.7 (64-bit) (National Institutes of Health, Bethesda, MD, USA).

### 2.15. Cellular ROS Accumulation

The concentration of 8-oxo-dGuo was measured by liquid chromatography–mass spectrometry as described previously [39,40]. Reduced glutathione (GSH) and oxidized glutathione (GSSG) concentrations (mmol/mg protein) were determined using a GSH assay kit (703002; Cayman Chemical Co., Ann Arbor, MI, USA) and calculated from a standard curve, with values normalized to protein concentration. NQO1 activity was assessed using a 2,6-dichlorophenolindophenol reduction assay, as described previously [40]. Net intracellular ROS accumulation was measured using the ROS-Glo™ H_2_O_2_ assay (Promega). Briefly, cells were treated with antioxidants or H_2_O_2_ for 2 h, washed twice with Hanks’ balanced salt solution (HBSS), and incubated with ROS-Glo™ Detection Solution for 20 min. Fluorescence was detected using a GloMax^®^ fluorometer (Promega) [41,42,43,44].

### 2.16. RNA Sequencing and Gene Clustering

RNA sequencing was performed by Welgene Biotech (Taipei, Taiwan) following the manufacturer’s protocol (Illumina, San Diego, CA, USA). cDNA libraries were prepared using TruSeq RNA Sample Prep Kits and sequenced on an Illumina GAIIx platform. Raw sequences were processed using the CASAVA Pipeline software 1.6 version, and low-quality reads were trimmed with ConDeTri. After filtering, qualified reads were analyzed using TopHat/Cuffdiff for gene expression estimation. Human Genome Build 19 and associated gene features were used for data processing. Gene expression levels were calculated as fragments per kilobase of transcript per million mapped reads (FPKM). Differentially expressed genes were identified using the following criteria: FPKM ≥ 0.3, fold change ≥ 2, and *p* < 0.05. Gene-level normalization was performed by transforming FPKM values into a log_2_ median-centered ratio. Clustering was conducted using Euclidean distance and complete linkage settings. Heatmaps were generated by coloring each gene according to its log_2_ median-centered ratio. Lists of liver cancer genes, oncogenes, tumor suppressor genes, stemness-related genes, and OCT4-signaling-associated genes were compiled from GSEA, KEGG, Gene Ontology, Life Technologies panels, and Qiagen panels. RNA sequencing data were deposited in the NCBI BioProject Database under accession number PRJNA273617 [30,41].

### 2.17. Measurements of Autophagy Activity

Autophagic activity was assessed using the Autophagy LC3 HiBiT Reporter Assay System [45]. U2OS HiBiT-HaloTag-LC3 cells were cultured in Dulbecco’s Modified Eagle Medium (DMEM) with high glucose, supplemented with 10% fetal bovine serum, 100 U/mL penicillin, and 100 U/mL streptomycin (8 × 10^3^ cells per 80 μL per well) in 96-well white clear-bottom plates. Cells were treated with unfermented or fermented *A. japonica* bark extracts, 20 μM Hir, 20 μM Ore, or 50 μM apigenin, luteolin, kaempferol, or quercetin for the indicated times, and autophagic activity was measured using the screening kit as described above. Autophagy responses were further quantified using plasmid-based reporters [43,44]. Plasmids pMRX-IP-GFP-LC3-RFP-LC3ΔG (#84572) and pMRX-IP-GFP-LC3-RFP (#84573) were obtained from the RIKEN DNA Bank (Tsukuba, Japan). rG2-DC-1C cells were transfected with these plasmids as described previously, and stable transfectants were established. Cells were then treated with the indicated compounds for 12 h in DMEM supplemented with 10% dialyzed fetal bovine serum. Autophagic flux was measured and calculated based on the GFP/RFP ratio as described [46,47].

### 2.18. Autophagosome Inhibitor

Chloroquine (10 μM; Sigma-Aldrich, C6628) was used as an autophagosome inhibitor [48] during rG2-DC-1C cell culture and invasion assays involving *A. japonica* bark extracts. Following treatment, protein levels of BAK and Bax were examined by Western blotting.

### 2.19. Invasion Assay

Cells (1 × 10^4^) were seeded onto Transwell inserts coated with Matrigel (1 mg/mL; Corning, NY, USA) in serum-free medium. The inserts were placed into wells containing DMEM supplemented with 10% FBS and incubated for 3 days in the presence or absence of 10 μM chloroquine (Selleck Inc., Yokohama, Japan; S6999). Treatments included unfermented or fermented *A. japonica* bark extracts, 20 μM Hir, 20 μM Ore, or 50 μM quercetin for the indicated times. Invaded cells on the lower surface of the membrane were fixed, stained, and counted under a microscope according to the manufacturer’s instructions [40].

### 2.20. Tumor Formation and Immunohistochemistry

rG2-DC-1C cells (200–500) were injected subcutaneously into SCID mice, and teratomas were examined by immunohistochemistry as described previously [30]. Cells were fixed in 4% formaldehyde for 10 min, washed with PBS, and incubated with blocking solution containing 10% FBS and 0.1% Triton X-100 in PBS for 15 min. Cells were then incubated overnight with primary antibodies. After washing with PBS containing 0.05% Tween-20, cells were incubated for 1.5 h with the following secondary antibodies: Alexa Fluor^®^ 594-labeled goat anti-rabbit IgG (Thermo Fisher Scientific; A-11037), Alexa Fluor^®^ 488-conjugated rabbit anti-goat IgG (Thermo Fisher Scientific; A-11078), and Alexa Fluor^®^ 647-labeled goat anti-rat IgG (H+L; Cell Signaling Technology; 4418). Nuclei were visualized using 4′,6-diamidino-2-phenylindole (DAPI; 1:3000 dilution; 5 mg/mL stock in DMSO; Sigma-Aldrich). Cells were mounted with ProLong^®^ Gold antifade reagent (Molecular Probes, Thermo Fisher Scientific; P36934), and immunofluorescence was observed using an Olympus FV1000 confocal laser scanning microscope.

### 2.21. Ingenuity Pathway Analysis (IPA)

Ingenuity Pathway Analysis (IPA; QIAGEN Inc., Hilden, Germany, release date 4 November 2025) was used to identify direct and indirect network molecules targeted by apigenin, luteolin, kaempferol, and quercetin. Targeted molecules for each compound were compared with those in the autophagy signaling pathway. Molecules common to all four drug networks and the autophagy pathway were retained in the final network. These common molecules were highlighted in the autophagy canonical signaling pathway using distinct color coding.

### 2.22. Statistical Analysis

Data are presented as mean ± standard error. Statistical comparisons between two groups were performed using Student’s *t*-test (two-tailed, paired). For multiple group comparisons, one-way analysis of variance (ANOVA) followed by Tukey’s post hoc test or two-way ANOVA followed with a Bonferroni post hoc test was applied depending on required analysis applied. All statistical analyses were conducted using GraphPad Prism 5.0 (GraphPad Software, San Diego, CA, USA). Differences were considered statistically significant at *p* < 0.05.

## 3. Results

### 3.1. Cellular Toxicity and Cancer Stemness Gene Characteristics of Fermented or Nonfermented Tree Bark Extracts in rG2-DC-1C Cells During Prolonged Cultivation

The morphology of rG2-DC-1C cells was not altered after 8 days of culture with unfermented and fermented tree bark extracts and 0.05% dimethyl sulfoxide (DMSO). By contrast, treatment with 20 μM Hir and 20 μM Ore resulted in small cell bodies and a round cellular phenotype in rG2-DC-1C cells (Figure S1A). We prepared cell culture media using extracts of unfermented or fermented *A. japonica* tree bark with *Frankia* fungi, or the diarylheptanoids Hir and Ore as the controls [12]. The cytotoxicity of these compounds was examined using rG2-DC-1C cells for 8 days (Figure S1B).

**Figure 1:**
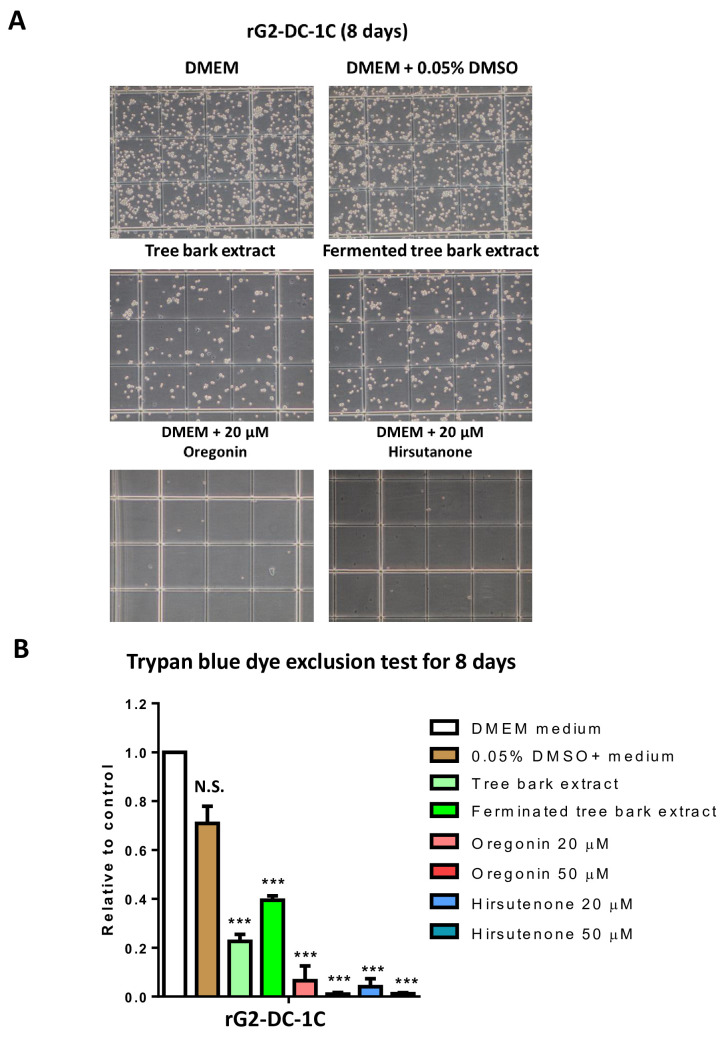

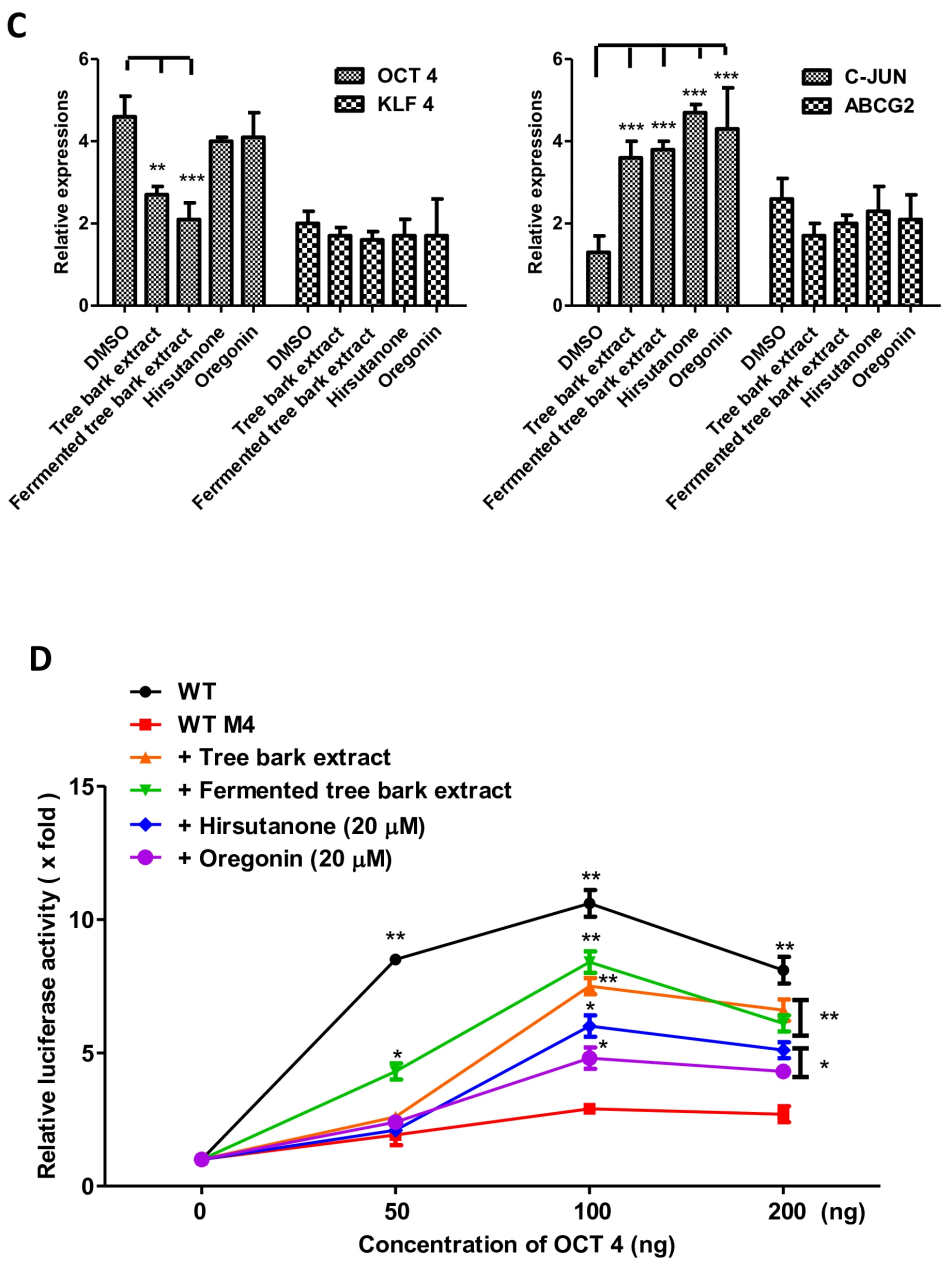
**Cell proliferation and cytotoxicity assay using trypan blue staining and characterization of cancer stemness of rG2-DC-1C.** (**A**) rG2-DC-1C were used for the cell growth assay as described elsewhere after treatment with 0.05% DMSO, unfermented and fermented tree bark extracts, and 20 μM of Hir and Ore. After 8 days of cultivation, rG2-DC-1C cells were stained using the trypan blue dye exclusion test, as described in Section 2. The relative cell numbers were calculated in (**B**). N.S. indicates not significant. *** indicates *p* < 0.001 (*n* = 4). (**C**) Relative expression of stemness genes *OCT4* and *KLF4* and cancer stemness genes *c-JUN* and *ABCG2*. qPCR expression studies were performed using the specific primers as described in Section 2. ** indicates *p* < 0.01, and *** indicates *p* < 0.001 (*n* = 5). (**D**) Relative luciferase activities of *c-JUN* promoter-(WT)-pGL4.1 luciferase and mutant M4 *c-JUN* promoter-pGL4.1 luciferase as described elsewhere [30]. The treatments of rG2-DC-1C clones co-transfected by *c-JUN* promoter-(WT)-pGL4.1 luciferase and various doses of OCT4 expression vector (pCEP_WT_Oct4) as described in Section 2, with unfermented and fermented tree bark extracts, and 20 μM of Hir and Ore as a control, were examined relative to treatment with 0.05% DMSO. * *p* < 0.05, and ** *p* < 0.05 (*n* = 3).

Proliferation was not reduced in rG2-DC-1C cells treated with either unfermented or fermented tree bark extract until 8 days; however, treatment with 10 μM Ore or 10 μM Hir inhibited cell proliferation after 8 days of culture. We also measured cell viability using various cytotoxicity assays, including the trypan blue dye exclusion test, over an 8-day cultivation period (Figure 1A). After 8 days of culture, unfermented and fermented tree bark extracts decreased cytotoxicity by 22–30% ± 2.5% and 38–42% ± 3.5% in rG2-DC-1C cells, respectively. By contrast, treatment with 20 μM Ore and 20 μM Hir markedly reduced the viability of rG2-DC-1C cells by 5.5–8.5% ± 0.5%, compared with control cultures or those treated with 0.05% DMSO (Figure 1B). Moreover, treatment with 50 μM Hir and 50 μM Ore significantly reduced the viability of rG2-DC-1C cells. Therefore, we used 20 μM Hir and 20 μM Ore, which showed modest cytotoxicity, as controls in subsequent experiments. Treatment with fermented tree bark extracts and unfermented tree extracts reduced by about 40 and 20% cytotoxicity in rG2-DC-1C cells. The nonspecific cytotoxic activity of the extract against rG2-DC-1C cells is noteworthy and warrants further investigation to identify the toxic components.

### 3.2. Characterization of RNA Sequencing and Gene Clustering of Cell Growth, Cell Cycle, and Death-Related Family Genes in Exposure of Fermented or Nonfermented Tree Bark Extracts

Regarding stemness features, we reported that rG2-DC-1C cells exhibited a greater than 20-fold increase in OCT4 expression and a 2.5- to 3.0-fold increase in KLF4 expression, but no alterations in SOX2 and MYC oncogene expression compared with HepG2 parental cancer cells were observed [30]. The expression of OCT4 significantly decreased by about 40–41% ± 0.5% after incubating unfermented or fermented tree bark extracts, without any reduction by treatments with 20 μM Hir and 20 μM Ore. (Figure 1C). However, we did not find the significant decreasing of KLF4 expressions by treatments with unfermented or fermented tree bark extracts, and 20 μM Hir and 20 μM Ore. In contrast, the expression of C-JUN as liver cancer stem cell markers increased about 3.0–3.5-fold in all treatment of tree bark extracts and 20 μM Hir and 20 μM Ore (Figure 1C). Analysis of serum dependency demonstrated that rG2-DC-1C cells can grow rapidly, even in a low serum concentration of 0.05%. Cell cycle analysis revealed that the proportion of S-phase cells in the rG2-DC-1C population (40%) was higher than that in the parental HepG2 cells (24%) [30]. Furthermore, we reported that the feedback of C-JUN and OCT4 played the critical role of the stemness features as liver cancer stem cells [30]. Thus, we examined the transactivation of *c-JUN*-promoter luciferase gene by an increased dose of OCT4 (Figure 1D). The treatment of *C-JUN* promoter by the increased dose of OCT4 resulted in the activation; however, the mutants of the OCT4 binding site in *C-JUN* promoter (M4) lost the *C-JUN* promoter activities. The OCT4 provided reasonable inductions of *C-JUN* promoter activation in the case of WT control cG2-DC-1C clone. The inductions of the *c-JUN* promoter activation were modest and reduced in the case of treatment with fermented or unfermented tree bark extracts and 20 μM Hir and 20 μM Ore. These results indicate the feedback regulation between OCT4 and c-JUN which maintains the stemness and cancer features of cG2-DC-1C clone. If the increased stemness character of OCT4 was expressed at higher levels, the cancer character of c-JUN was repressed when treated with fermented or unfermented tree bark extracts and 20 μM Hir and 20 μM Ore (Figure 1C,D). We will study this feedback regulation further in the future. RNA sequencing data of rG2-DC-1C cells and their parental HepG2 cells were analyzed to detect the upregulation of various genes, such as those involved in the cell cycle and growth (Figure S2A), including cyclin D3, bone morphogenetic protein receptor 1B (BMPR1B), protein kinase C, protein phosphatase 2, inhibitor of DNA binding 2 (ID2; a transcriptional regulator that lacks a basic DNA-binding domain), Jun transcription factor, and T-cell factor 7 (TCF7)/lymphoid enhancer-binding factor. Some of these encoded proteins bind to β-catenin, activating transcription through the WNT signaling pathway. The downregulated genes identified were clusters of differentiation 44 (CD44), leukemia inhibitory factor, and SMAD9 (Figure S2A,B).

Regarding BMP and transforming growth factor (TGF)-β/SMAD signaling, the expression of BMPR1B, BMP8b, and SMAD7 increased in rG2-DC-1C cells compared with parental HepG2 cells. In addition, the expression of connective tissue growth factor, CD44, BMP1, TGF-α, TGF-β receptor III, and SMAD9 decreased in rG2-DC-1C cells. Taken together, our findings suggest that SMAD9 functions as a novel type of transcriptional regulator within the BMP signaling pathway. Thus, the BMP–TGF-β/Smad pathway may mediate cell death signaling (Figure S2B).

Regarding apoptosis-related genes, BCL3, BCL2A1, TCF7, SMAD7, and BAK1 were significantly upregulated in rG2-DC-1C cells. By contrast, the gene expression of BAG cochaperone 4, BCL-2-modifying factor, BCL7A, SMAD9, BMP1, BMPR1A, SMAD1, TCF23, TCF7L1, and CD44 was downregulated in rG2-DC-1C cells (Figure S2C). Regarding autophagy-related genes, the expression of V-type proton ATPase subunit E1 (ATP6VDE1), CD63, GABA type A receptor-associated protein like 2, ATP6VOC, cathepsin D (CTSD), CTSA, ATP6VOB, autophagy-related protein 9B (ATG9B), ATG4A, and sequestosome 1 was increased. However, the expression of CTSB, WD repeat domain phosphoinositide-interacting protein 1, neighbor of BRCA1 gene 1 protein, tripeptidyl-peptidase 1, ATPV1B2, glucosamine (N-acetyl)-6-sulfatase, chloride voltage-gated channel 3 (CLCN3), ATG2B, ATG4A, and CLCN4 was decreased (Figure S2D).

### 3.3. Comparative ROS Activity in rG2-DC-1C Treated with Fermented or Nonfermented Tree Bark Extracts

**Figure 2:**
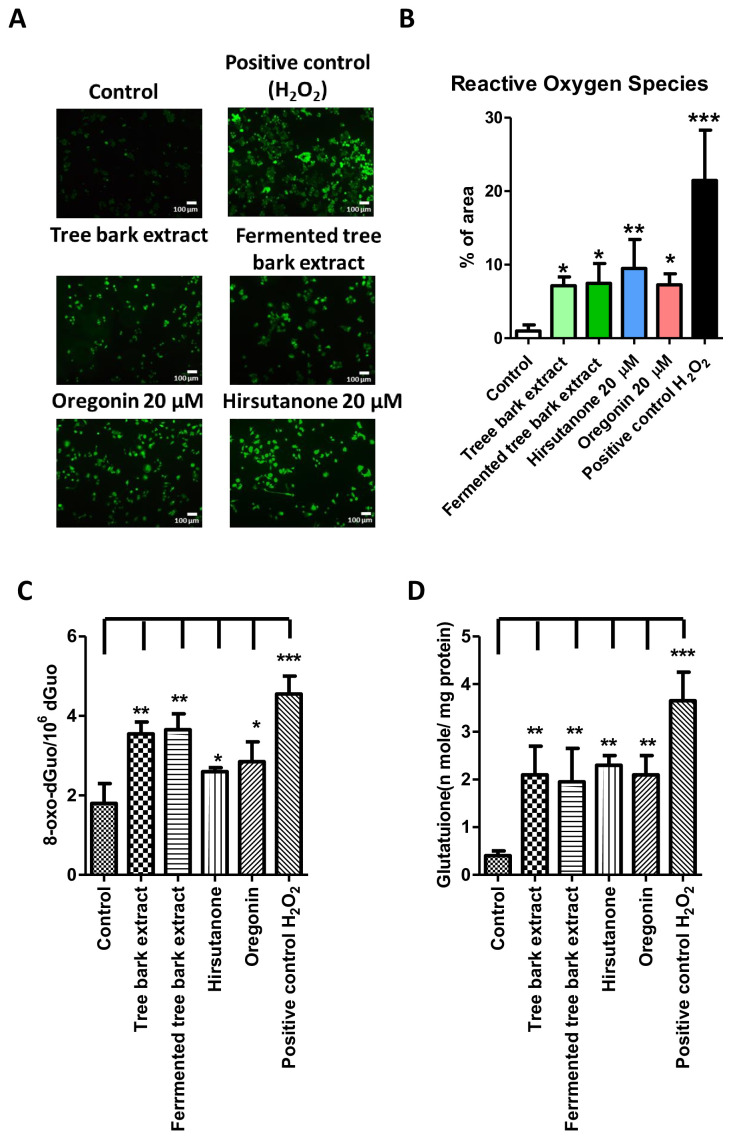

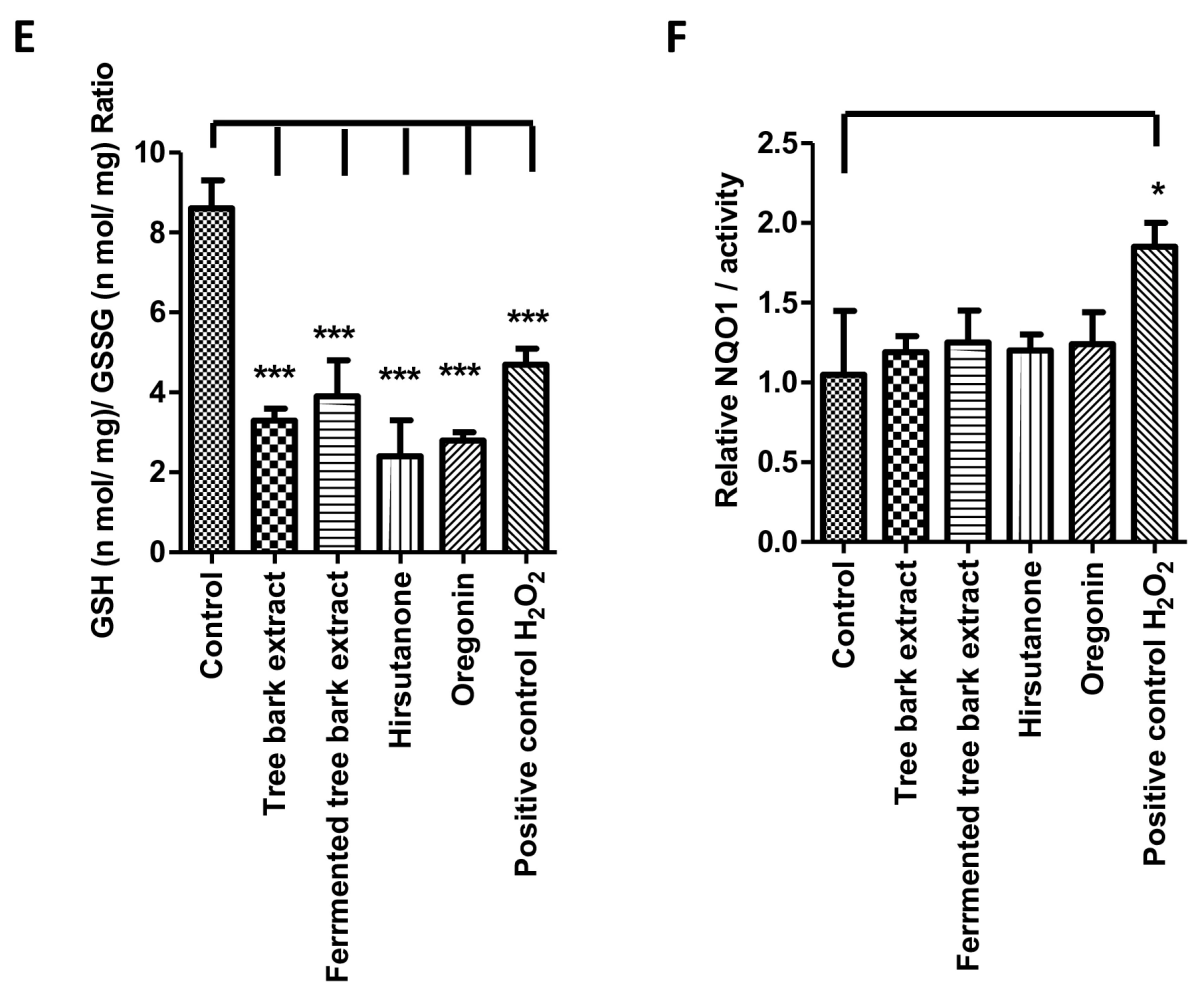
**ROS, 8-oxo-dGuo/10^6^ dGuo, and GSH levels, GSH/GSSG ratio, and relative NQO1 activity in rG2-DC-1C cells after various treatments.** (**A**) rG2-DC-1C cells were incubated with unfermented and fermented tree bark extracts, 20 μM Hir and 20 μM Ore for 24 h, and ROS levels were measured by staining with CM-H_2_DCFDA, as described elsewhere [30]. After washing the cells, fluorescence was measured and analyzed using ImageJ version 1.54p run with Java 21.0.7 (64-bit) software, as described elsewhere. H_2_O_2_ was taken as positive control. (**B**) The quantitative data were calculated as the % of the fluorescent intensities of cell areas. ROS activity of the control (0.05% DMSO) was set at 1.0%. *, **, and *** indicate *p* < 0.05, *p* < 0.01, and *p* < 0.001, respectively (*n* = 5). (**C**) Levels of 8-Oxo-dGuo/10^6^ dGuo in rG2-DC-1C cells were measured in response to unfermented and fermented tree bark extracts and Hir and Ore as controls for 24 h. (**D**) Determination of total glutathione (nmole/mg protein) in rG2-DC-1C cells treated with unfermented and fermented tree bark extracts and Hir and Ore as controls for 24 h. (**E**) Determination of the GSH/GSSG (nmol/mg GSH/nmol/mg GSSG) ratio in rG2-DC-1C cells after various treatments for 24 h. *** indicates *p* < 0.001 (*n* = 5). (**F**) Relative NQO1 activity was measured after 24 h. Data are presented as the mean ± standard error of mean (SEM; *n* = 3). Data were analyzed using one-way analysis of variance (ANOVA) with Tukey’s post hoc test (* *p* < 0.05).

To understand the role of tree bark components in ROS production in rG2-DC-1C cells, we used chloromethyl-2′,7′-dichlorofluorescein diacetate and flow cytometry to measure ROS production, as described previously [39,40,41,42,43]. Unfermented and fermented tree bark extracts significantly induced ROS production by 6.0 ± 1.1-fold and 6.5 ± 2.2-fold, respectively, as determined by calculating the relative ROS-positive cell numbers per total cell numbers compared with the control (0.05% DMSO) culture (Figure 2A,B). Control 20 μM Hir and 20 μM Ore cultures exhibited 8.0 ± 2.8-fold and 6.2 ± 1.0-fold higher ROS activities compared with the control 0.05% DMSO culture.

The increase in ROS production was regulated by the aryl hydrocarbon receptor (AhR) pathway, as confirmed by comparing *AhR* luciferase activity in rG2-DC-1C cells. A significant increase in *AhR* promoter activity was observed, as evaluated by measuring wild-type *AhR* luciferase expression (Figure S3). Treatment with fermented or unfermented tree bark extracts and 20 μM Hir and 20 μM Ore resulted in comparable increases in *AhR* luciferase activity in rG2-DC-1C cells; about 1.5-, 1.5-, 2.4-, and 2.5-fold higher *AhR*-promoter activities compared with 0.05% DMSO culture, respectively. These increases were significantly reduced with the DRE2-mutated *AhR* construct. This indicates that AHR–nuclear factor erythroid 2-related factor 2 (NRF2)–Jun dimerization protein 2 (JDP2) axis is critical for both treatments with fermented or unfermented tree bark extracts and 20 μM Hir and 20 μM Ore [40]. ROS activation may be one of the resultant targets of CSC-mediated tumor invasion and tumor development.

Furthermore, we examined another oxidative stress marker, 7,8-dihydro-8-oxo-27-deoxyguanosine (8-oxo-dGuo), which is a marker of DNA oxidation. Its levels were higher after treatment with unfermented or fermented tree bark extract and 20 μM Hir and 20 μM Ore (approximately 1.9-, 2.0-, 1.38-, and 1.56-fold, respectively) compared with those in control 0.05% DMSO culture. Indeed, the positive control hydrogen peroxide (H_2_O_2_) treatment resulted in more than 2.5-fold increase of 8-oxo-dGuo levels (Figure 2C). The total glutathione (GSH) levels were approximately 5.5-, 5.25-, 6.0-, 5.5-fold higher after treatment with unfermented or fermented tree bark extract, 20 μM Hir and 20 μM Ore compared with those in control 0.05% DMSO culture. The positive control H_2_0_2_ showed about 9.5-fold increase as compared with that of 0.05% DMSO culture (Figure 2D). The GSH/oxidized GSH (GSSG) ratio was also calculated. Treatments with unfermented and fermented tree bark extracts and 20 μM Hir and 20 μM Ore reduced the GSH/GSSG ratio by approximately 39.0, 46.7, 29.0, 33.3%, respectively, compared with that in control 0.05% DMSO culture. The control H_2_O_2_ treatment showed a 55.5 ± 3.1% reduction in this ratio (Figure 2E). In contrast, the NAD(P)H: quinone oxidoreductase (NQO) activity did not increase significantly after treatments with tree bark extracts and Hir and Ore (Figure 2F). These findings were consistent with changes in ROS production levels.

### 3.4. Expression of AHR–NRF2 Axis and Cell Death-Related Genes After Treating with Fermented or Nonfermented Tree Bark Extracts

**Figure 3:**
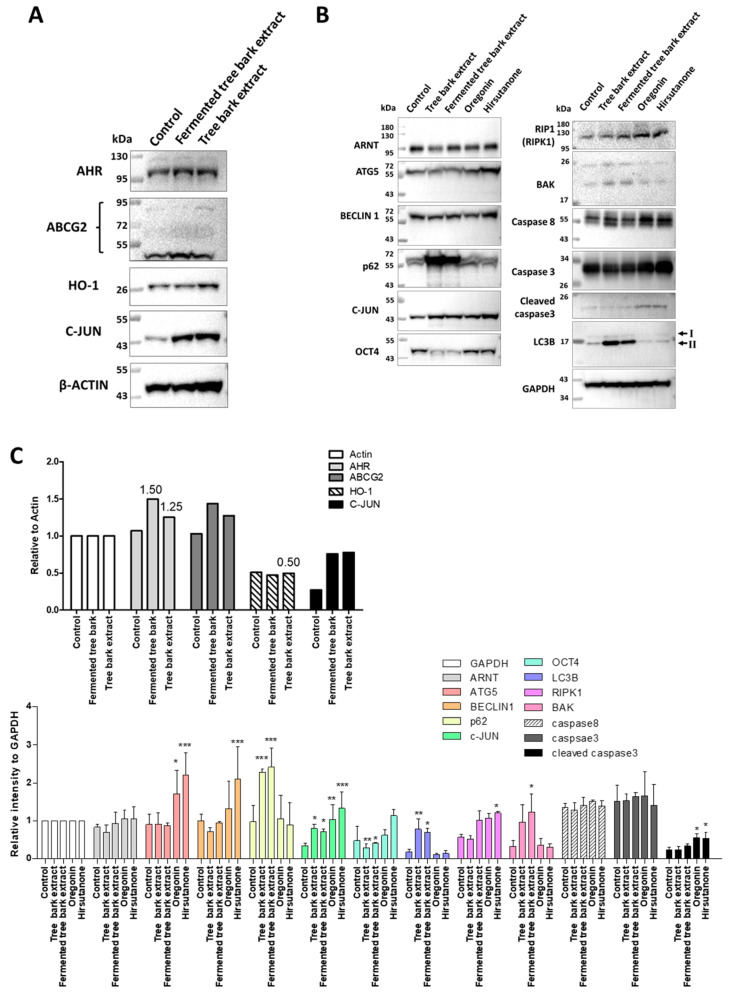

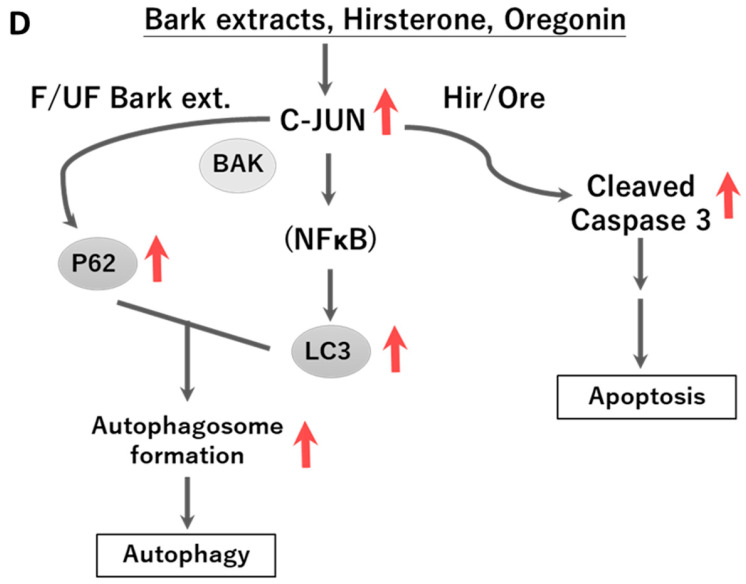
**Western blots of stem cell marker proteins, oxidative stress/antioxidation-related proteins, and cell death-related proteins after induction.** 40 μg of protein was fractionated on 10% sodium dodecyl sulfate polyacrylamide gels and transferred to membranes. (**A**) Then, the membranes were incubated with primary antibodies against AHR, ABCG2, HO-1, NQO1, and c-JUN. Subsequently, secondary antibodies were applied to visualize the intensities of each band. The intensity of each band was then quantified, and the relative value was normalized to that of β-actin. (**B**) The membranes were incubated with primary antibodies against ARNT, ATG5, BECLIN 1, p62, c-JUN, OCT4, HO-1, RIP1, BAK, Caspase-8, cleaved and uncleaved Caspase-3, and LC3B I and II. Then, secondary antibodies were applied. The intensity of each band was quantified, and the relative value was normalized to that of GAPDH. (**C**) The relative values of individual repeats (n ≥ 4) were normalized to those of β-actin or GAPDH and shown as ratios of one-way ANOVA and were used with a post hoc Tukey HSD compared between control group and each column treatment of specific protein. The significance shown in asterisk as * *p* < 0.05, ** *p* < 0.01, *** *p* < 0.001. (**D**) Schematic model linking unfermented or fermented tree bark extracts and Hir and Ore to autophagy and apoptosis, respectively. NFkB; nuclear factor kappa B.

We compared the protein expression of stemness and CSC markers, the AHR–NRF2 axis genes, and cell death-related genes (Figure 3). We reported that rG2-DC-1C showed that TP53 expression was reduced by 4.8 ± 2.5-fold, but cyclin D3 expression was increased compared with that in HepG2 cells (3.3 ± 0.7-fold) [30]. c-JUN expression in rG2-DC-1C cells after treatment with unfermented or fermented tree bark extract was significantly increased compared with that in control cells (2.8 ± 0.3-fold and 3.1 ± 0.2-fold, respectively; Figure 3A,C). Hir and Ore treatment also led to a similar increase in c-JUN expression (3.0 ± 0.2-fold, 3.6 ± 0.3-fold, respectively, Figure 3B,C). Regarding the OCT4–JUN auto-amplification cascade, which promoted cancer stemness features, Jun was involved in the maintenance of self-renewal and tumorigenicity in glioma stem-like cells [49]. Kuo et al. demonstrated that OCT4 cross-regulated JUN, creating a feedback loop [30]. Treatment with unfermented or fermented tree bark extract, as well as Hir and Ore, displayed distinct and varying effects on the OCT4–JUN cascade. Indeed, the interaction between OCT4 and c-JUN was not functional in rG2-DC-1C cells upon treatment with either unfermented or fermented tree bark extract because OCT4 expression was reduced by 43–58% ± 1.5%, although c-JUN expression was induced about 2-fold. This might be one reason why these extracts led to a reduction in tumorigenesis, as described below. When the OCT4 expression was increased in rG2-DC-1C cells, c-JUN promoter activations were activated in the case of unfermented or fermented tree bark extracts (Figure 1D). Thus, in this liver cancer stem-like cell, rG2-DC-1C, the OCT4-c-JUN positive regulatory feedback circuit seems to be functional in unfermented or fermented tree bark extracts.

The expression of cell death-related proteins was also examined (Figure 3B,C). Caspase-3 expression increased by approximately 1.3 ± 0.5-fold and 1.5 ± 0.5-fold in rG2-DC-1C cells treated with 20 μM Hir and 20 μM Ore, respectively. In addition, the expression of cleaved Caspase-3 was significantly increased under these conditions (by 3.8 ± 1.2-fold and 3.5 ± 0.6-fold, respectively). Regarding the expression of caspase family members, Hir treatment resulted in the activation of caspases 3, 8, and 9 and the cleavage of PARP in leukemia and colon cancer cells [17]. Hir induced apoptosis mediated by the TRAIL protein by increasing the activities of Caspase-8 and the BID-dependent pathway [22]. By contrast, treatment with *A. japonica* hot water extract (AJHW) and Ore effectively prevented muscle cell apoptosis, promoted the synthesis of muscle proteins, and inhibited their degradation in vitro. AJHW and Ore treatment significantly increased cell viability and reduced apoptosis by upregulating BCL-2 and downregulating BCL-2-associated X protein (BAX), cleaved Caspase-3, and cleaved PARP [50]. These findings indicate that AJHW and Ore could serve as therapeutics to prevent and treat sarcopenia [50].

However, treatment with unfermented or fermented tree bark extract decreased the expression of uncleaved Caspase-3 by about 0.8-fold (Figure 3B,C). BAK expression was increased by treatment with unfermented or fermented tree bark extract (by 2.5 ± 0.7-fold and 2.8 ± 0.5-fold, respectively, Figure 3B,C). By contrast, the expression of receptor-interacting serine/threonine-protein kinase 1 (RIP1), which is a necrosis marker, was not significantly increased by treatment with unfermented or fermented tree bark extract but was significantly increased upon treatment with Hir and Ore (by 2.2 ± 0.5-fold, 2.3 ± 0.3-fold) compared with that in control cells. Interestingly, the expression of autophagy markers p62 and light chain 3B (LC3B; involved in autophagosome formation) was increased by 2.0–2.3 ± 0.2-fold and 3.3–4.2 ± 0.3-fold, respectively, upon treatment with unfermented or fermented tree bark extract. However, treatment with Hir and Ore did not lead to higher changes in their expression compared with that in control cells. Other autophagy markers, such as ATG5 and BECLIN 1, were not significantly altered, but showed a slight decrease in expression by treatment with unfermented or fermented tree bark extract (by 75–90%) (Figure 3B,C).

Next, expression of the oxidative stress-related gene AHR was examined. The expression of AHR proteins showed the moderate increased about 1.5 ± 0.3-fold and 1.25 ± 0.3-fold, respectively (Figure 3A,C). We also found that treatment with unfermented or fermented tree bark extract increased ROS levels and AHR luciferase activity in rG2-DC-1C cells compared with those in the control cells. Thus, activation of AHR by both unfermented and fermented tree bark extracts can induce ROS production, primarily through autophagy rather than apoptosis; however, Hir and Ore treatment induced the Caspase-3 mediated apoptosis reaction to induce the cell death of CSCs (Figure 3D).

### 3.5. Transwell Invasion Assay by Exposure of Fermented or Nonfermented Tree Bark Extracts

**Figure 4:**
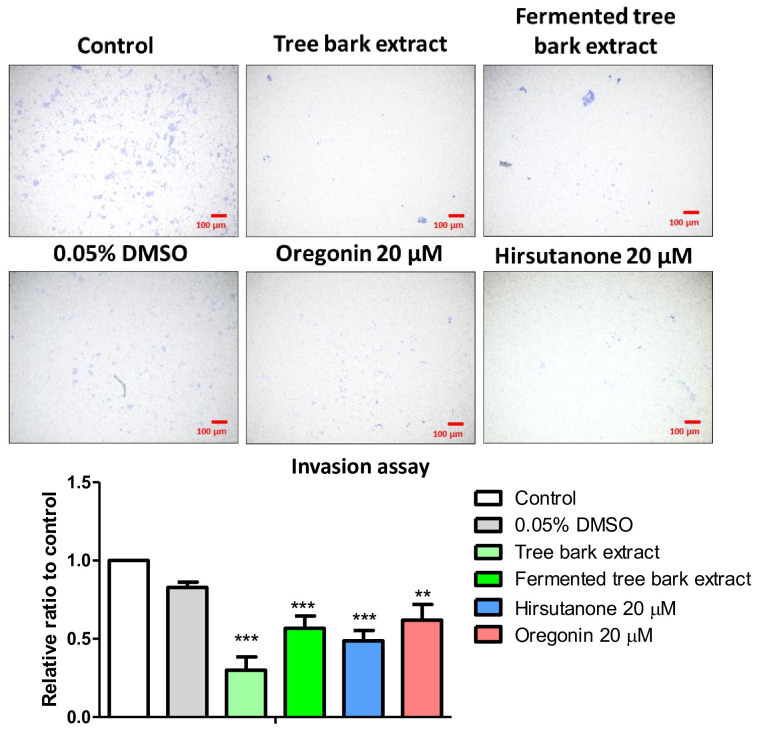
**Invasion activities of rG2-DC-1C cells after treatment.** rG2-DC-1C cells were examined for invasion potency after treatment with unfermented or fermented tree bark extracts, as described elsewhere [30]. The stained migrated cells were detected and quantified as described in Methods and Materials. Data are presented as the mean ± SEM (*n* = 3) and were analyzed using an unpaired, two-tailed Student’s *t*-test (** *p* < 0.01, *** *p* < 0.001). The bar represents the average of triplicates with the standard deviation.

Transwell invasion assay demonstrated that the invasion activity of rG2-DC-1C cells was 9-fold higher than that of HepG2 cells, as previously reported [30]. Treatment with unfermented and fermented tree bark extracts resulted in a significant reduction in invasion activity (by 25–30 ± 2.5% and 50–60 ± 1.5%, respectively) (Figure 4). Control treatment with 20 μM Hir and 20 μM Ore showed a decrease in invasion activity (by 45–60%). Thus, both unfermented and fermented tree bark extracts significantly reduced CSC-invasion activity.

### 3.6. Xenotransplantation Assay Exhibits the Suppression of Tumor Formation of rG2-DC-1C by Treatment with Fermented or Nonfermented Tree Bark Extracts

**Figure 5:**
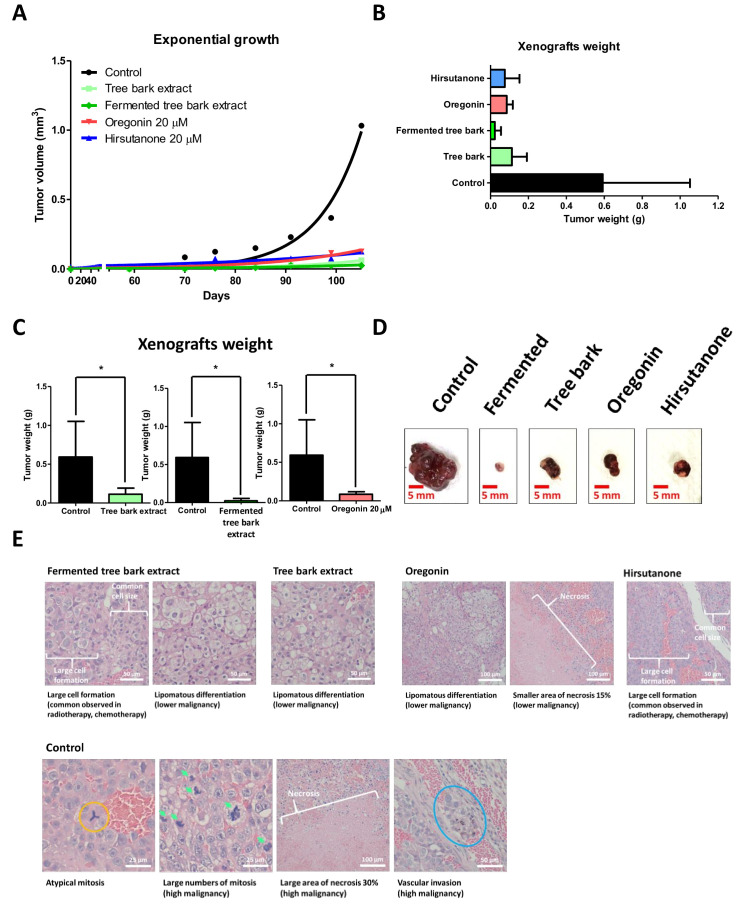
**Tumor formation assays of rG2-DC-1C cells after various treatments.** (**A**) Xenografts after the injection of 10^4^ rG2-DC-1C cells in the left/right flank of SCID male mice. Tumors were harvested 105 days after injection. The tumor sizes and weights were measured. Quantification analysis of clonogenic efficiency over time. The bar graphs show clonogenic efficiency in rG2-DC-1C cells. The treatments were shown to be different in colors. (**B**,**C**) The weight of xenografts and the morphologies of cancer grafts were assessed after treatment with the indicated tree bark extracts and Hir and Ore. The data are presented as the mean ± SEM (n  =  3). * Indicates *p* < 0.05. Data were analyzed using a one-way ANOVA with Tukey’s post hoc test. (**D**) One example of the tumor xenografts after treatments of various tree bark extracts and Hir and Ore. 5 mm is the scale bars. (**E**) Histochemistry of tumors derived from rG2-DC-1C cells treated with unfermented and fermented tree bark extracts and Hir and Ore in SCID mice. Typical cancerous physiologies of the grafts were observed. rG2-DC-1C clones exhibited the typical malignant mitosis of cancer cells, accompanied by higher necrosis; vascular invasion was also observed in some cases. Furthermore, giant cells and abnormal mitosis were identified as markers of necrosis. The unfermented and fermented tree bark extracts showed large cell formation and lipomatous differentiated cell types. The orange area, green arrowhead, and blue circle indicate cancer-like traits, and the white line indicates necrosis. Scar bars are indicated in 25, 50, and 100 μm.

To verify the tumor regression activities of unfermented and fermented tree bark extracts in CSC clones, we performed xenotransplantation experiments. We found that the colony formation activity of rG2-DC-1C cells was 3.2- to 5.0-fold higher than that of HepG2 cells [30]. To confirm this finding, xenotransplantation experiments using rG2-DC-1C cells as CSCs were performed. A total of 5 × 10^6^ rG2-DC-1C cells were injected to obtain a tumor size of 1.15 cm^2^ and tumor weight of 0.38 g in severe combined immunodeficiency (SCID) mice [30]. Although control rG2-DC-1C cells showed variable tumor growth, a significant reduction in tumor formation was observed upon treatment with unfermented and fermented tree bark extracts (by 2.5–8.0 ± 0.2% to obtain a tumor size of 1.15 cm^2^ and tumor weight of 0.38–0.1 g) and with 20 μM Hir and 20 μM Ore as the control (by 4.1–8.2% ± 1.1%), as calculated by comparing tumor weights (Figure 5A–D).

Thus, both unfermented and fermented tree bark extracts showed a reduction in the CSC potency of rG2-DC-1C cells, like that of Hir- or Ore-treated CSC clones. Histological analysis confirmed that both unfermented and fermented tree bark extracts reduced cancer phenotypes compared with control rG2-DC-1C cells, which displayed clear cancer histology (Figure 5E).

These findings suggest that unfermented and fermented tree bark extracts can inhibit the development of tumors driven by CSCs. rG2-DC-1C clones exhibited atypical mitosis of cancer cells, accompanied by increased necrosis; vascular invasion was observed in some cases. Furthermore, necrotic giant cells and abnormal mitotic activity were detected. However, treatment with unfermented and fermented tree bark extracts led to the formation of large, differentiated lipomatous cells that exhibited low-grade malignancy. These findings suggest that treatment with unfermented and fermented tree bark extracts suppresses the progression of CSC-derived hepatocellular carcinoma (HCC).

### 3.7. Autophagy-Inducing Components in A. japonica Bark Extracts

The specific constituents responsible for autophagy-mediated CSC death remain incompletely defined (Supplementary Table S1) [18]. Because the full compositional analysis of the fermented extract is ongoing in collaboration with the company, we acknowledge this limitation and emphasize that future studies are required to identify and validate the specific molecules responsible for the enhanced activity.

While Hir, Ore, and lignans primarily triggered apoptosis, several flavonoids identified in bark extracts—including apigenin-7,4′-dimethyl ether, luteolin-7,4′-dimethyl ether, kaempferol, acacetin, lupenone, taraxarone, platyphylloside, acerogenin L, platyphyllone, and quercetin—have been implicated in autophagy [18,51,52,53,54,55,56]. Among these, luteolin, kaempferol, apigenin, and quercetin were selected for further analysis. Luteolin, kaempferol, and apigenin have demonstrated dual apoptotic and autophagic activity [52,53,54,55], whereas quercetin is consistently reported as a potent autophagy inducer [51,56,57,58,59].

Ingenuity Pathway Analysis (IPA) [30] revealed that these flavonoids converge on autophagy-related signaling, with quercetin showing strong association with mTOR inhibition (Figures S4A–H and S5A–C). Functional assays using LC3 HiBiT reporters confirmed that quercetin, but not Hir or Ore, induced autophagic flux (Figure S6A). GFP/RFP-LC3 assays further demonstrated reduced GFP/RFP ratios following quercetin treatment, consistent with enhanced autophagy (Figure S6B). These findings suggest that quercetin is a key autophagy-inducing component of bark extracts, although contributions from other flavonoids cannot be excluded.

### 3.8. Effects of an Autophagy Inhibitor on Autophagy and BAK Expression Mediated by Fermented or Nonfermented Tree Bark Extracts

**Figure 6:**
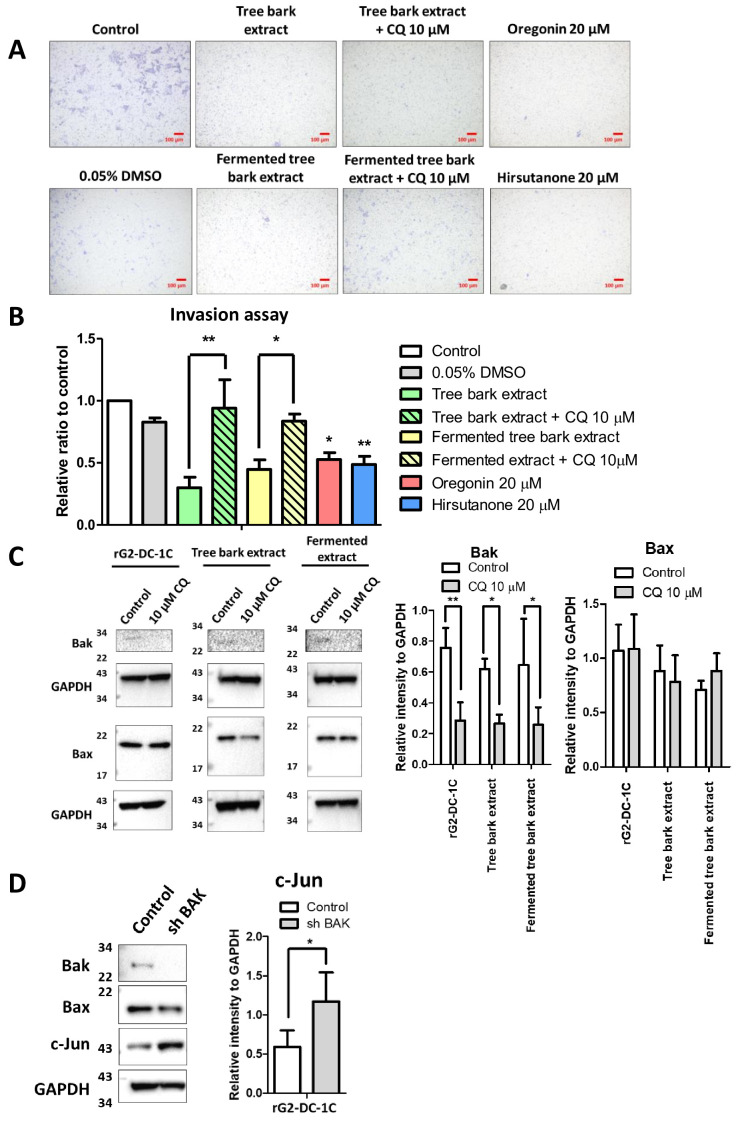

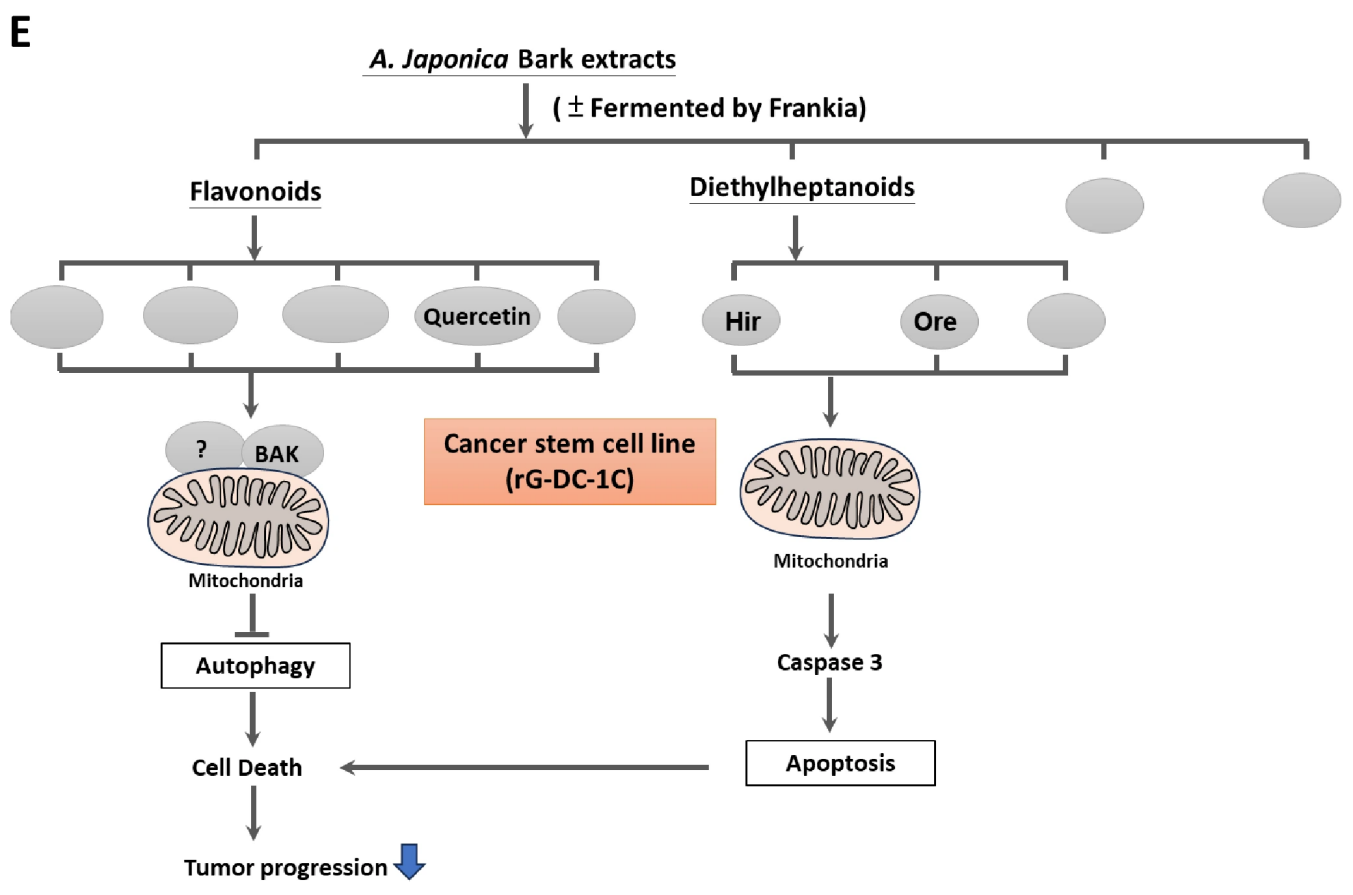
**Effect of chloroquine on the invasion activity of rG2-DC-1C cells after treatment with unfermented or fermented tree bark extracts.** (**A**) The rG2-DC-1C cells were incubated with 10 μM CQ during the invasion assay, as described elsewhere. The stained migrated cells were detected and quantified as described elsewhere [37]. (**B**) Data are presented as the mean ± SEM (*n* = 3) and were analyzed using an unpaired, two-tailed Student’s *t*-test (* *p* < 0.05, ** *p* < 0.001, *n* = 3). Scale bars: 100 μm. The bar represents the average of triplicates with the standard deviation. (**C**) Western blots of BAK and BAX proteins after treatment with CQ and quantification of their expression. The relative values of individual repeats (*n* ≥ 4) were normalized to those of GAPDH and shown as ratios of two-way ANOVA followed with a Bonferroni post hoc test compared between control group and each column treatment of specific targets. The significance shown in asterisk as * *p* < 0.05, ** *p* < 0.01. (**D**) Western blots of sh BAK rG2-DC-1C and quantification of c-Jun expression. The relative values of individual repeats (*n* = 3) were normalized to those of GAPDH and shown as ratios of two-way ANOVA followed with a Bonferroni post hoc test compared between control group and each column treatments of specific targets. The significance shown in asterisk as * *p* < 0.05. (**E**) Schematic modeling of the cell death functions of flavonoids and diethylheptanoids from *A. Japonica* which are shown in the present study. The flavonoids induced the BAK-mediated autophagy reaction, and Hir and Ore mediated the Caspase-3 mediated apoptosis reaction.

Western blot analysis showed that unfermented and fermented bark extracts increased BAK expression and autophagic markers, whereas Hir and Ore induced Caspase-3 cleavage without autophagy (Figure 3D, Figure 6A,B and Figure S6). Treatment with the autophagy inhibitor chloroquine (CQ) [48] suppressed BAK expression (Figure 6C) and reversed the anti-invasive effects of bark extracts, confirming the role of BAK in autophagy-mediated tumor suppression (Figure 6A,B). Notably, CQ reduced BAK expression in CSC rG2-DC-1C cells more strongly than in parental HepG2 cells, while BAX remained unchanged (Figure 6C). shRNA-mediated BAK knockdown yielded similar results, reinforcing BAK’s pivotal role in autophagy induction (Figure S6).

Quercetin was identified as the primary flavonoid component responsible for autophagy induction and CSC suppression, consistent with prior reports of its activity in liver cancer and early-phase clinical studies [56,57,58,59]. However, synergistic effects of other flavonoids within bark extracts remain possible and warrant further investigation.

## 4. Discussion

The rG2-DC-1C clone exhibited cancer stem cell (CSC)-like properties, reflected by elevated OCT4 and c-JUN expression. Consistent with clinical data, c-JUN was detected in nearly half of human hepatocellular carcinoma (HCC) specimens, with a strong correlation to OCT4 positivity [30]. Interestingly, treatment with unfermented and fermented *Alnus japonica* bark extracts reduced OCT4 expression by more than 50%, while c-JUN levels were increased. In contrast, the diarylheptanoids Hirsutenone (Hir) and Oregonin (Ore) did not affect OCT4 (Figure 3B,C), underscoring mechanistic differences between crude extracts and isolated compounds.

Programmed cell death encompasses apoptosis, autophagy, necrosis, pyroptosis, and ferroptosis, each defined by distinct morphological and molecular signatures [60,61,62]. Apoptosis typically involves caspase activation, chromatin condensation, and DNA fragmentation [62]. However, in our study, apoptosis was not the primary driver of CSC suppression by bark extracts. Instead, autophagy was strongly implicated, as evidenced by a 2–4-fold increase in LC3B and p62 expression, reduced proliferation, and diminished colony formation, invasion, and tumorigenesis (Figure 3B,C). By contrast, Hir and Ore induced apoptosis, confirmed by cleaved Caspase-3 expression, alongside antiproliferative and anti-invasive activity (Figure 3B,C). Both compounds also triggered BAK expression, consistent with their known pro-apoptotic activity [12,13,14,15,16,17,18,19]. Previous reports have demonstrated Hir’s broad anticancer activity across leukemia, colon, ovarian, prostate, thyroid, and epithelial carcinoma cell lines [21,22,23,24], while Ore acts as a weaker analog and potential prodrug of Hir. Ore additionally exhibits antioxidant, anti-inflammatory, and metabolic regulatory properties, supporting its pharmacological relevance [13,20,26,27].

Our xenotransplantation assays further confirmed that both unfermented and fermented bark extracts suppressed tumor formation and invasion in vivo, reducing tumor incidence to below 5% over 105 days (Figure 5 and Figure 6). Our study demonstrates that *Alnus japonica* fermented or nonfermented bark extracts suppress CSC-driven tumorigenesis primarily through BAK-mediated autophagy, a mechanism that distinguishes them from the apoptotic effects of Hir and Ore (Figure 6E). Both mechanisms function in the extract complementarily rather than exclusively. The autophagy inhibitor chloroquine (CQ) and *shBAK* attenuated BAK expression and invasion, reinforcing the role of BAK-dependent autophagy in CSC suppression (Figure 6 and Figure S6) [48,57,60,63]. Prior studies have shown that BAX/BAK activation can initiate AMPK phosphorylation, autophagic flux, and mitochondrial clearance, highlighting the dual roles of these proteins in cell death regulation [64,65,66]. Notably, only BAK, not BAX, was detected in our system, suggesting a unique pathway of BAK-mediated autophagy in CSCs.

Although quercetin and related flavonoids are known to induce autophagy and suppress tumor growth in diverse cancers, our findings highlight a distinctive mechanism: BAK-mediated autophagy without concomitant BAX activation or Caspase-3 cleavage (Figure 6E). This suggests that additional flavonoid components within bark extracts may synergize with quercetin to promote CSC death. Further studies are warranted to dissect the contribution of individual compounds and to explore the impact of fermentation on bioactivity.

## 5. Conclusions

Our study demonstrates that *Alnus japonica* bark extracts potentially suppress CSC-driven liver tumorigenesis primarily through BAK-mediated autophagy—a mechanism that distinguishes them from the apoptotic effects of Hir and Ore. By establishing natural extracts as robust autophagy modulators, we provide a mechanistic foundation for harnessing phytochemicals in liver cancer prevention and therapy. These findings position *A. japonica* bark as a promising candidate for next-generation autophagy-based interventions. Future work should explore fermentation-enhanced activity and conduct rigorous preclinical validation to advance these extracts toward clinically actionable strategies, thereby strengthening the role of natural products in cancer therapeutics. Our findings demonstrate that whole bark extracts of *A. japonica* suppress CSC-like tumorigenicity via autophagy, while purified Hir and Ore act through apoptosis, highlighting complementary mechanisms that together broaden the therapeutic potential of this traditional medicinal plant.

## Data Availability

The datasets used and analyzed during the current study are available from the corresponding author on reasonable request. The RNA sequencing data were deposited in the NCBI Bioproject Database with the accession number PRJNA273617 (www.ncbi.nlm.nih.gov/bioproject/?term=PRJNA273617, accessed on 26 January 2015).

## Supplementary Materials

The following supporting information can be downloaded at: https://www.mdpi.com/article/10.3390/antiox15060685/s1.

## Abbreviations

AhR	Aryl hydrocarbon receptor
BAK	B-cell lymphoma 2 (BCL-2) antagonist/killer
BAX	BCL-2-associated X protein
BCL2	B-cell lymphoma 2
CHCl3–MeOH	chloroform–methanol
CSC	cancer stem cells
DMEM	Dulbecco’s Modified Eagle Medium
DMSO	dimethyl sulfoxide
EdU	5-ethynyl-2-deoxyuridine
EtOAc	Ethyl acetate
*Hir *	Hirstenone
HO-1	heme oxygenase-1
iPSCs	induced pluripotent stem cell
MEF	Mouse embryonic fibroblasts
NRF2	Nuclear factor 2-like factor 2
Ore	Oregonin
PARP	poly (adenosine diphosphate-ribose) polymerase
PBS	phosphate-buffered saline
PCR	polymerase chain reaction
ROS	reactive oxygen species
SCID	severe combined immunodeficiency
TNF	tumor necrosis factor
VDAC2	Voltage-dependent anion channel 2
