# Supplementary material for: Natural Extracts of Alnus japonica Induce BAK-Dependent Autophagy to Inhibit Liver Cancer Stem Cell Tumorigenesis
Source: Antioxidants (Basel). 2026 May 29;15(6):685. doi: 10.3390/antiox15060685 (PMC13296072; doi:10.3390/antiox15060685)
Supplement: Supplementary file 1 [file antioxidants-15-00685-s001.zip › antioxidants-4260875-supplementary.pdf]

Supplementary Data of Western blotting

Natural Extracts of *Alnus japonica* Induce BAK-Dependent Autophagy to Inhibit Liver Cancer Stem Cell Tumorigenesis

Kenly Wuputra[1-4], Yoshimasa Matsuura[5], Satoshi Gushiken[6], Hirosuke Fukuda[6], Ya-Han Yang[4], Chia-Che Ku[1-3,7], Chun-Chieh Wu[8,9], Ying-Chu Lin[10], Yi-Chun Tsai[4,10,11], Deng-Chyang Wu[3,7,9], Toshihiko Nozaki[12], Kohsuke Kato[13], Atsushi Kawaguchi[13], Kyosuke Nagata[13], Yoshiharu Tanaka[14*], and Kazunari K. Yokoyama[1-3,15*]

1 Cell Therapy Research Center, Department of Medicine, Kaohsiung Medical University Hospital, Kaohsiung 80756, Taiwan.

2 Graduate Institute of Medicine, Kaohsiung Medical University, Kaohsiung 80708, Taiwan.

3 Regenerative Medicine and Cell Research Center, Kaohsiung Medical University, Kaohsiung 80708, Taiwan.

4 Division of Nephrology, Department of Internal Medicine, Kaohsiung Medical University Hospital, Kaohsiung 80756, Taiwan.

5 Faculty of Liberal Arts and Sciences, Osaka Prefecture University, Osaka 599-8531, Japan.

6 Okinawa Eco-Science Co., Ltd., Okinawa 905-2261, Japan.

7 Division of Gastroenterology, Department of Internal Medicine, Kaohsiung Medical University Hospital, Kaohsiung 80756, Taiwan.

8 Director of Forensic Pathology Division, Department of Pathology, Kaohsiung Medical University Hospital, Kaohsiung 80756, Taiwan.

9 Department of Medicine, Kaohsiung Medical University, Kaohsiung 80708, Taiwan.

10 School of Dentistry, Kaohsiung of Medical University, Kaohsiung 80756, Taiwan.

[11] Division of Nephrology, Department of Internal Medicine, Kaohsiung Medical University CiJin Hospital, Kaohsiung 805, Taiwan.

[12] Ryukyu Fertilizer Co., Ltd., Okinawa 904-2162, Japan.

[13] Department of Infection Biology, Institute of Medicine, University of Tsukuba, Tsukuba 305-8575, Japan.

[14] Radiation Biology and Molecular Genetics, Division of Quantum Radiation, Faculty of Technology, Osaka Metropolitan University, Osaka 599-8531, Japan.

[15] Research Institute for Biomedical Sciences, Tokyo University of Science, Noda 2780022, Japan.

*Corresponding authors: Kazunari Yokoyama, Ph.D. Medical Researcher, Professor, Cell Therapy and Research Center Kaohsiung Medical University Hospital and Graduate Institute of Medicine Kaohsiung Medical University, Phone +886-7321-1101; FAX +886-7313-3849, e-mail Kazu@kmu.edu.tw; Yoshiharu Tanaka, Ph.D. Radiation Biology and Molecular Genetics, Division of Quantum Radiation, Faculty of Technology, Osaka Metropolitan University, Osaka 599-8531, Japan. Phone and FAX, +81-72-254-9750, e-mail, dudgi905@outlook.jp (t21355k@omu.ac.jp). ORCID of KK Yokoyama; 0000-0001-8508-7587; K Wuputra; 0000-0003-4026-7052; CC Ku; 0000-0002-1496-3081; YC Tsai; 0000-0003-4923-3342; CC Wu; 0000-0001-8776-3302; YC-Liu; 0000-0002-0681-3796; DC-Wu; 0000-0003-3742-0634; Y Tanaka; 0000-0002-3126-328X, and YC Lin; 0000-0002-2499-8632.

1. **Materials and Methods**
2. **Supplementary Figures**

   Supplementary Figure S1. Microscopic morphology and cell proliferation/cytotoxicity of rG2-DC-1C cells after various treatments.

   Supplementary Figure S2. Comparative analysis between rG2-DC-1C and HepG2 cells.

   Supplementary Figure S3. Relative AHR-luciferase activities in wild-type and BRD2 mutant constructs in rG2-DC-1C cells.

   Supplementary Figure S4. Ingenuity pathway analysis (QIAGEN Inc. released Nov. 4, 2025) was used to identify the network of extract components and the molecules of autophagy pathways.

   Supplementary Figure S5. The proposed common network signaling of four flavonoids (apigenin, luteolin, kaempferol, and quercetin) and the autophagy pathway.

   Supplementary Figure S6. Light chain 3 HiBiT receptor assays and relative GFP/RFP ration value of autophagy reaction using apigenin, luteolin, kaempferol, and quercetin.

Supplementary Figure S7. Full-length western blotting is used in this study.

Supplementary Figure S8. Bright view images taken in this study.

3. **Supplementary Table S1. Chemical constituents of the tree bark extract from *Alnus japonica*.**
4. **References**

5. Materials and Methods

5.1. Animals and reagents

Mouse embryonic fibroblasts (MEFs), HepG2, SNL76/7, 293, and 293T cells were obtained from the American Type Culture Collection (Manassas, VA, USA) and the RIKEN Cell Bank (Tsukuba, Ibaraki, Japan). Cells were cultured in Dulbecco's Modified Eagle Medium (DMEM; Gibco, Grand Island, NY, USA) with or without high glucose. All media were supplemented with 10% fetal bovine serum (FBS; Gibco) and 1% penicillin–streptomycin (P/S; Gibco). Animal experiments were approved by the National Laboratory Animal Center (Taiwan), Kaohsiung Medical University (Taiwan), and the Animal Care Committee of the RIKEN BioResource Research Center (Japan). All procedures complied with the animal welfare guidelines of the National Laboratory Animal Center (protocol no. 106022), Kaohsiung Medical University (protocol nos. 106189, 107128, and 108244), and the RIKEN BioResource Research Center (Kiteisv.intra.riken.jp/JoureiV5HTMLContents/act/print/print110000514.htm).

Experiments were conducted in accordance with these guidelines approved. Hirsutanone (Hir) and Oregonin (Ore) were purchased from Sigma-Aldrich, Inc. (SMB00096 and SMB00088; St. Louis, MO, USA; Merck KGaA, Darmstadt, Germany). Apigenin (cat. no. 010-18914) was obtained from Fujifilm Wako Pure Chemicals (Osaka,

Japan). Luteolin (cat. no. 491-70-3) was purchased from Sigma-Aldrich, Inc. Kaempferol (cat. no. 520-18-3) and quercetin (cat. no. 849061-97-8) were obtained from Tokyo Kasei Co. (Tokyo, Japan). Chloroquine diphosphate (C6628) was purchased from Sigma-Aldrich, Inc. (St. Louis, MO, USA; Merck KGaA, Darmstadt, Germany). Torin 1 (CAS 1222998-36-8) was obtained from NJ, USA, and Bafilomycin A (CAS 88899-55-2) was purchased from AdipoGen Life Sciences (San Diego, CA, USA).

5.2. Generation of unfermented and fermented tree bark extracts of A. japonica

A. japonica has been cultivated in the Yanbaru district of Okinawa since its introduction from Taiwan (*Alnus formosana*) in 1910. Gushiken et al. reported the preparation of fermented bark extracts from *A. japonica* roots coexisting with the symbiotic fungus *Frankia*, yielding fermented tree extracts that were developed as agricultural antibacterial materials [31]. A voucher specimen of *A. japonica* stem bark was deposited at the University of the Ryukyus, Okinawa, Japan [32]. Stems, leaves, and rhizomes of *A. japonica* were gently washed to remove *Frankia* fungi, then mixed and cut into small pieces using an electric cutter (Hitachi Inc., Ibaraki, Japan). The pieces were fermented at room temperature for 7–10 days, dried, and ground into powder. During fermentation, the materials were stirred occasionally to ensure uniform processing, allowing the surfaces to be covered with white mycelium and promoting bacterial growth. For preparation of unfermented extracts, the fermentation step was omitted. For extraction, water was added at a ratio of 100 L per 1 m^3 of plant material, followed by boiling and distillation. Hirsutanone (Hir) and oregonin (Ore) were used as positive controls.

5.3. Isolation of Polyphenols, Flavonoids, and Other Secondary Metabolites

Previous phytochemical studies of A. japonica identified diarylheptanoids, triterpenoids, and flavonoids [2–4,19]. To extend these findings, boiled extracts of fermented and unfermented tree bark (1.0 kg) were prepared with 50% aqueous EtOH (2.0 L, three times) at 40 °C under sonication. After solvent removal, the residue was resuspended in 1.0 L of water and partitioned successively with CH_2Cl_2 and EtOAc (1.0 L each, three times), yielding CH_2Cl_2-soluble (35.0 g) and EtOAc-soluble (53.5 g) fractions.

A portion of the EtOAc fraction (40.0 g) was fractionated on silica gel using a $CHCl_3$–MeOH gradient (15:1 to 0:1, v/v), producing six fractions (F1–F6). Fraction 2 (2.6 g) was further chromatographed as described [33], with silica gel ($CHCl_3$–MeOH, 10:1), followed by RP-C18 chromatography (MeOH–H_2O, 1:1), yielding compound 5 (hirsutanone, 60 mg). Fraction 4 (4.5 g) was subjected to RP chromatography (MeOH–H_2O, 6:5), generating seven subfractions (F2.1–F2.7). Subfraction 2.1 (1.8 g) was re-chromatographed on silica gel ($CHCl_3$–MeOH, 6:1), affording compound 4 (oregonine, 1.6 g). Fraction 6 (80 mg) was chromatographed with EtOAc–MeOH (15:1), yielding compounds 1 (18 mg) and 3 (15 mg). Compound 2 (11 mg) was purified from subfraction 2.7 (350 mg) using silica gel chromatography ($CHCl_3$–MeOH, 5:1). The lignan [(−) -(2R,3R)-1,4-O-diferuloylsecoisolariciresinol, DFS] was extracted with methanol [20]. Concentrated extracts (50 g) were partitioned with $CHCl_3$, and the soluble fraction (4.8 g) was chromatographed on silica gel using hexane/EtOAc, yielding three fractions. Fraction 2 (300 mg) was subjected to RP chromatography (gradient 30–80% MeOH), affording DFS (27.5 mg). Purity was confirmed by HPLC Major flavonoids (quercetin, kaempferol,

myricetin, apigenin, luteolin) were extracted by refluxing boiled bark extract with aqueous methanol (62.5%) containing tertiary butylhydroquinone (2 g/L) and HCl (final concentration 1.2 M in 50% aqueous methanol) at 90 °C for 2 h [33]. The cooled extract was filtered, adjusted to 50 mL with methanol, and passed through a 0.45 μm membrane filter prior to HPLC analysis. Extracts were stored in amber bottles at −20 °C until use.

Flavonoid aglycones were quantified by RP-HPLC (Nova-Pak C18, 3.9 × 150 mm, 4 μm; Waters, Milford, MA) using MeOH/H_2O (50:50, v/v, pH 2.5 with trifluoroacetic acid) as mobile phase, UV detection at 365 nm, and a flow rate of 1 mL/min. Chromatograms were compared with authentic standards. All determinations were performed duplicate. Structures were confirmed by LC-MS, 1D/2D NMR, and comparison with reported data [34]. In general, NMR spectra were recorded at 100 and 270 MHz (^{1}HNMR), and 25.05 MHz (^{13}CNMR). Chemical shifts are given in 6 (ppm) with TMS as int. std. Negative FAB-MS were measured at 1.5 kV (accelerating voltage) with Me & O-glycerol as matrix. High-resolution techniques including HPLC, UHPLC-QqQ-MS/MS (Agilent Technologies, Wilmington, DE, USA), LC-ESI-MS/MS (Agilent Technologies, Wilmington, DE, USA, coupled with a hybrid quadrupole linear ion trap mass spectrometer QTRAP® 5500 (AB Sciex, Foster City, CA, USA) equipped with an electrospray ionization source (ESI), were employed for separation, identification, and quantification of phenolic compounds [35]. Coupling HPLC with UV-visible or diode array detectors (HPLC-UV, HPLC-DAD; Shimadzu Corp. Kyoto, Japan), as well as GC-MS (Shimadzu Corp. Kyoto, Japan), enabled sensitive detection of flavonoids and phenolic acids. QTOF-MS (Shimadzu Corp. Kyoto, Japan), provided accurate molecular mass determination, facilitating identification of novel bioactive compounds in tree bark extracts.

5.4. Generation of rG2-DC-1C cells

Recombinant lentiviruses encoding human OSKM (RDB08323, RDB08324, RDB12904 in RIKEN BRC) C-JUN (RDB06254 in RIKEN BRC), and shBAK (TRCN000033464; siRNA Core Facility at Academia Sinica (Taipei, Taiwan) were produced in 293T cells co-transfected with *pCAG-HIVgp* and p*CMV-VSV-G-RSV-Rev* by the Lentivirus preparation protocol in RIKEN BioResource Center, Japan as described elsewhere [30]. HepG2 cells and the iPSHep FB/Ng/gfp-103C-1 cell line were infected with lentiviruses at a multiplicity of infection (MOI) of 50 and incubated for 1 week in DMEM [30]. Subsequently, cells were transferred onto mitomycin C (Sigma-Aldrich)-treated MEFs in DMEM supplemented with ESGRO (Merck KGaA, Darmstadt, Germany; 10 ng/mL) to generate iPSC-like cells from HepG2 cells. A single colony (approximately 200 cells) of HepG2-derived iPSC-like cells was injected into severe combined immunodeficiency (SCID) mice. Tumor formation was observed approximately 9 weeks later. The tumor was resected, and a primary cancer cell line was established, designated "reprogrammed HepG2-derived cancer cells from one colony" (rG2-DC-1C). This cell line was used for subsequent experiments.

5.5. Cell viability assays using MTT and trypan blue dye exclusion

Cell viability was assessed using the 3-[4,5-dimethylthiazol-2-yl]-2,5 diphenyl tetrazolium bromide (MTT) assay as described previously [36]. Cells (3×10^4) were treated with unfermented or fermented tree bark extracts, as well as the indicated concentrations of Hir and Ore, at 37 °C for indicated time periods. Following treatment, 10 µL of MTT solution (10 mg/mL) was added to each well and incubated for 2 h at 37 °C.

After centrifugation at 412 × g for 5 min, the medium was removed, and 100 μL of dimethyl sulfoxide (DMSO; Sigma) was added to dissolve the formazan crystals. Absorbance was measured at 570 nm using a microplate reader (Immuno Mini NJ-2300, Nihon InterMed, Tokyo, Japan) during the culture periods until 8 days. Cell viability was expressed relative to the absorbance of untreated control cultures. Cell viability was also determined using the trypan blue dye exclusion assay as described elsewhere [37]. Briefly, cells were stained with trypan blue and counted using a hemocytometer to determine the number of viable cells in suspension. Proliferation rates were calculated based on viable cell counts.

5.6. Cell proliferation assay and apoptosis

For the cell proliferation assay, cells were plated and cultured for 3~8 days under environmental oxygen conditions (20%), followed by treatment with 10 μM 5-ethynyl-2-deoxyuridine (EdU) for 6 h. Growing cells were stained with Alexa Fluor azide, and total cells were counterstained with Hoechst using the Click-iT EdU Assay Kit (C10337, Thermo Fisher Scientific, Waltham, MA, USA). Fluorescence microscopy was used to image stained cells. The percentage of proliferating cells was calculated by dividing the number of EdU-positive cells by the total number of Hoechst-stained cells. Cell counts were obtained from five randomly selected fields using Cell Count software (version 1.1.7). For apoptosis analysis, cells were incubated for 24 h at 37 °C. Caspase-3 and -7 activities, key indicators of apoptotic pathways, were measured using the Caspase-Glo 3/7 Assay Kit (Promega). Cell viability was additionally assessed using the Trypan blue dye exclusion assay, in which viable cells were quantified by hemocytometer counting

5.7. Isolation of RNA and quantitative PCR (qPCR)

Total RNA was extracted using the PureLink™ RNA Mini Kit (Invitrogen). RNA was reverse transcribed into cDNA using SuperScript III Reverse Transcriptase (Invitrogen) [39]. Quantitative real-time PCR was performed on a StepOne or ABI7500 instrument (Applied Biosystems, Foster City, CA, USA) using Fast SYBR® Green Master Mix (Applied Biosystems) in 20 μL reaction volumes. Threshold cycle (Ct) values were averaged from technical duplicates. Transcript levels of target genes were normalized to Gapdh expression. Relative gene expression was calculated using the 2^–ΔΔCt method, with expression levels normalized to DMSO-treated rG2-DC-1C cells (set as 1.0). Data are presented as mean ± SEM from three biological replicates.

The primer sequences used were as follows:

- OCT4: forward 5′- GGGTTTTTGGGATTAAGTTCTTCA-3′,
  reverse 5′-GCCCCCACCCTTTGTGTT-3′
- SOX2: forward 5′- GCTACAGCATGATGCAGGACCA-3′,
  reverse 5′- TCTGCGAGCTGGTCATGGAGTT-3′
- KLF4: forward 5′-CATCTCAAGGCACACCTGCGAA-3′,
  reverse 5′- TCGGTCGCATTTTTGGCACTGG-3′
- C-JUN: forward 5′-CCTTGAAAGCTCAGAACTCGGAG-3′,
  reverse 5′-TGTCTGCGTTAGCATGAGTTGGC-3′

5.8. Construction of AhR or c-Jun promoter luciferases and their corresponding cis-element mutant luciferases

AhR promoter regions [39,40] were cloned into the pGL4.1 plasmid (Promega, Madison, WI, USA) as described previously [40]. The orientation and integrity of the constructs were confirmed by restriction enzyme digestion and next-generation sequencing. Putative binding sites within the AhR promoter region were predicted using ALGGEN-PROMO (http://alggen.lsi.upc.edu). Site-directed mutagenesis of individual promoter sites,

including DRE2, was performed using the QuickChange Lightning Site-Directed Mutagenesis Kit (Agilent Technologies, Santa Clara, CA, USA) [39,40].

Wild type and mutant M4 c-JUN promoter were generated and transfected into rG2-DC-1C cells as described previously [30]. Two days after transfection, cells were harvested and measured for luciferase. The WT c-JUN promoter and M1 to M4 mutants were cotransfected with various amounts (0–200 ng) of OCT4-expressing plasmids into rG2-DC-1C cells.

5.9. Transient transfection and luciferase reporter assay

rG2-DC-1C cells were seeded into 24-well plates (4 × 10^4 cells/well) and cultured for 24 h. Cells were cotransfected with 500 ng of the AhR luciferase plasmid and 10 ng of the pRL-CMV plasmid encoding *Renilla* luciferase using either Lipofectamine 2000 (Invitrogen) or polyethylenimine (linear, molecular weight 25,000; Polysciences, Warrington, PA, USA; Cat# 23,966). The total amount of transfected DNA was maintained at 1 μg/well by supplementing with pBluescript II SK+ control plasmid (Addgene, Watertown, MA, USA). The human OCT 4 expression vector (pCEP4_WT_OCT4) was gift from James Thomson (Addgene Plasmid #40629; http://n2t.net/addgene:40629; RRID: Addgene_40629) and used for cotransfecting rG2-DC-1C cells.

Transfected cells were treated with DMSO for the indicated times and harvested 48 h post-transfection. Luciferase activity was measured using the Dual-Luciferase Reporter Assay System (Promega) according to the manufacturer's instructions, with detection performed using a GloMax 20/20 Luminometer (Promega). Reporter activity was

calculated as the ratio of Firefly luciferase to Renilla luciferase and expressed as fold induction relative to the empty vector in rG2-DC-1C cells [30]. All measurements were performed in duplicate, and values are presented as mean ± standard error of the mean (SEM) from at least three independent experiments.

5.10. shRNA lentivirus and autophagosome inhibitor

shRNA lentiviruses targeting human BAK1 (TRCN0000033464) were obtained from the siRNA Core Facility at Academia Sinica (Taipei, Taiwan). Predesigned ON-TARGETplus SMARTpool siRNA against human BAK1 and scrambled control siRNA were purchased from GE Dharmacon (Austin, TX, USA). Mouse embryonic fibroblasts (MEFs) were seeded into six-well plates (for Western blotting) or 24-well plates (for luciferase reporter assays) and transfected with 20–40 nM of either siRNA or control RNA in OPTI-MEM medium (0.5 mL for 24-well plates; 2 mL for six-well plates) using Lipofectamine RNAiMAX (Invitrogen). For rG2-DC-1C cells, shRNA was transduced at a multiplicity of infection (MOI) of 10. After 24 h, fresh culture medium containing 10% FBS was added, and cells were subsequently transfected with luciferase plasmids for reporter assays as described above. Knockdown efficiency was confirmed 48 h post-infection by immunoblotting and complementary analyses. To inhibit autophagosome formation, 10 μM chloroquine was added during rG2-DC-1C cell culture and invasion assays involving A. japonica bark extracts. Following treatment, protein levels of BAK, Bax, Bad, Bcl-2, and p62 were examined by Western blotting.

5.11. ROS detection using CM-H2DCFDA fluorescence

Reactive oxygen species (ROS) levels were measured as described previously [40]. rG2-DC-1C cells were cultured in 0.1% gelatin-coated 12-well plates with or without tree bark extracts (unfermented or fermented), or with 20 μM Hir or Ore for the indicated times. As positive controls, cells were treated with 150–200 μM hydrogen peroxide for 15 min or with 50 μM apigenin, luteolin, kaempferol, or quercetin for the indicated times before CM-H2DCFDA addition. Cells were rinsed with warm Hanks' balanced salt solution (HBSS; Gibco Invitrogen, Waltham, MA, USA) and incubated with 10 μM CM-H2DCFDA (C-6827; Life Technologies) in complete growth medium for 30 min at 37 °C in the dark. After treatment, cells were washed twice with HBSS and examined using a Nikon inverted fluorescence microscope. Five randomly selected fields were imaged using a 10× objective lens, and fluorescence intensity was quantified with ImageJ software (National Institutes of Health, Bethesda, MD, USA).

5.12. Cellular ROS accumulation

The concentration of 8-oxo-dGuo was measured by liquid chromatography–mass spectrometry as described previously [39,40]. Reduced glutathione (GSH) and oxidized glutathione (GSSG) concentrations (mmol/mg protein) were determined using a GSH assay kit (703002; Cayman Chemical Co., Ann Arbor, MI, USA) and calculated from a standard curve, with values normalized to protein concentration. NQO1 activity was assessed using a 2,6-dichlorophenolindophenol reduction assay, as described previously [40]. Net intracellular ROS accumulation was measured using the ROS-Glo™ H_2O_2 assay (Promega). Briefly, cells were treated with antioxidants or H_2O_2 for 2 h, washed twice with Hanks' balanced salt solution (HBSS), and incubated with ROS-Glo™ Detection

Solution for 20 min. Fluorescence was detected using a GloMax® fluorometer (Promega) [42-44].

5.13. RNA sequencing and gene clustering

RNA sequencing was performed by Welgene Biotech (Taipei, Taiwan) following the manufacturer's protocol (Illumina, San Diego, CA, USA). cDNA libraries were prepared using TruSeq RNA Sample Prep Kits and sequenced on an Illumina GAIIx platform. Raw sequences were processed using the CASAVA Pipeline software, and low-quality reads were trimmed with ConDeTri. After filtering, qualified reads were analyzed using TopHat/Cuffdiff for gene expression estimation. Human Genome Build 19 and associated gene features were used for data processing. Gene expression levels were calculated as fragments per kilobase of transcript per million mapped reads (FPKM). Differentially expressed genes were identified using the following criteria: FPKM ≥ 0.3, fold change ≥ 2, and $p < 0.05$. Gene-level normalization was performed by transforming FPKM values to a $\log_2$ median-centered ratio. Clustering was conducted using Euclidean distance and complete linkage settings. Heatmaps were generated by coloring each gene according to its $\log_2$ median-centered ratio. Lists of liver cancer genes, oncogenes, tumor suppressor genes, stemness-related genes, and OCT4-signaling-associated genes were compiled from GSEA, KEGG, Gene Ontology, Life Technologies panels, and Qiagen panels. RNA sequencing data were deposited in the NCBI BioProject Database under accession number PRJNA273617 [30,41].

5.14. Measurements of autophagy activity

Autophagic activity was assessed using the Autophagy LC3 HiBiT Reporter Assay System [45]. U2OS HiBiT-HaloTag-LC3 cells were cultured in Dulbecco's Modified Eagle Medium (DMEM) with high glucose, supplemented with 10% fetal bovine serum, 100 U/mL penicillin, and 100 U/mL streptomycin (8×10^3 cells per 80 µL per well) in 96-well white clear-bottom plates. Cells were treated with unfermented or fermented A. japonica bark extracts, 20 µM Hir, 20 µM Ore, or 50 µM apigenin, luteolin, kaempferol, or quercetin for the indicated times, and autophagic activity was measured using the screening kit as described above. Autophagy responses were further quantified using plasmid-based reporters [43,44]. Plasmids pMRX-IP-GFP-LC3-RFP-LC3ΔG (#84572) and pMRX-IP-GFP-LC3-RFP (#84573) were obtained from the RIKEN DNA Bank (Tsukuba, Japan). rG2-DC-1C cells were transfected with these plasmids as described previously, and stable transfectants were established. Cells were then treated with the indicated compounds for 12 h in DMEM supplemented with 10% dialyzed fetal bovine serum. Autophagic flux was measured and calculated based on the GFP/RFP ratio as described [46,47].

5.15. Autophagosome inhibitor

Chloroquine (10 µM; Sigma-Aldrich, C6628) was used as an autophagosome inhibitor [48] during rG2-DC-1C cell culture and invasion assays involving A. japonica bark extracts. Following treatment, protein levels of BAK and Bax were examined by Western blotting.

5.16. Invasion assay

Cells (1 × 10^4) were seeded onto Transwell inserts coated with Matrigel (1 mg/mL; Corning, NY, USA) in serum-free medium. The inserts were placed into wells containing DMEM supplemented with 10% FBS and incubated for 3 days in the presence or absence of 10 μM chloroquine (Selleck Inc., Japan; S6999). Treatments included unfermented or fermented A. japonica bark extracts, 20 μM Hir, 20 μM Ore, or 50 μM quercetin for the indicated times. Invaded cells on the lower surface of the membrane were fixed, stained, and counted under a microscope according to the manufacturer's instructions [40].

5.17. Tumor formation and immunohistochemistry

rG2-DC-1C cells (200–500) were injected subcutaneously into SCID mice, and teratomas were examined by immunohistochemistry as described previously [30]. Cells were fixed in 4% formaldehyde for 10 min, washed with PBS, and incubated with blocking solution containing 10% FBS and 0.1% Triton X-100 in PBS for 15 min. Cells were then incubated overnight with primary antibodies. After washing with PBS containing 0.05% Tween-20, cells were incubated for 1.5 h with the following secondary antibodies: Alexa Fluor® 594-labeled goat anti-rabbit IgG (Thermo Fisher Scientific; A-11037), Alexa Fluor® 488-conjugated rabbit anti-goat IgG (Thermo Fisher Scientific; A-11078), and Alexa Fluor® 647-labeled goat anti-rat IgG (H+L; Cell Signaling Technology; 4418). Nuclei were visualized using 4′,6-diamidino-2-phenylindole (DAPI; 1:3000 dilution; 5 mg/mL stock in DMSO; Sigma-Aldrich). Cells were mounted with ProLong® Gold antifade reagent (Molecular Probes, Thermo Fisher Scientific; P36934), and immunofluorescence was observed using an Olympus FV1000 confocal laser scanning microscope.

5.18. Ingenuity Pathway Analysis (IPA)

Ingenuity Pathway Analysis (IPA; QIAGEN Inc., release date November 4, 2025) was used to identify direct and indirect network molecules targeted by apigenin, luteolin, kaempferol, and quercetin. Targeted molecules for each compound were compared with those in the autophagy signaling pathway. Molecules common to all four drug networks and the autophagy pathway were retained in the final network. These common molecules were highlighted in the autophagy canonical signaling pathway using distinct color coding.

5.19. Statistical analysis

Data are presented as mean ± standard error. Statistical comparisons between two groups were performed using Student's T-test (two-tailed, paired). For multiple group comparisons, one-way analysis of variance (ANOVA) followed by Tukey's post hoc test was applied. All statistical analyses were conducted using GraphPad Prism 5.0 (GraphPad Software, San Diego, CA, USA). Differences were considered statistically significant at $p < 0.05$.

Supplementary Figures

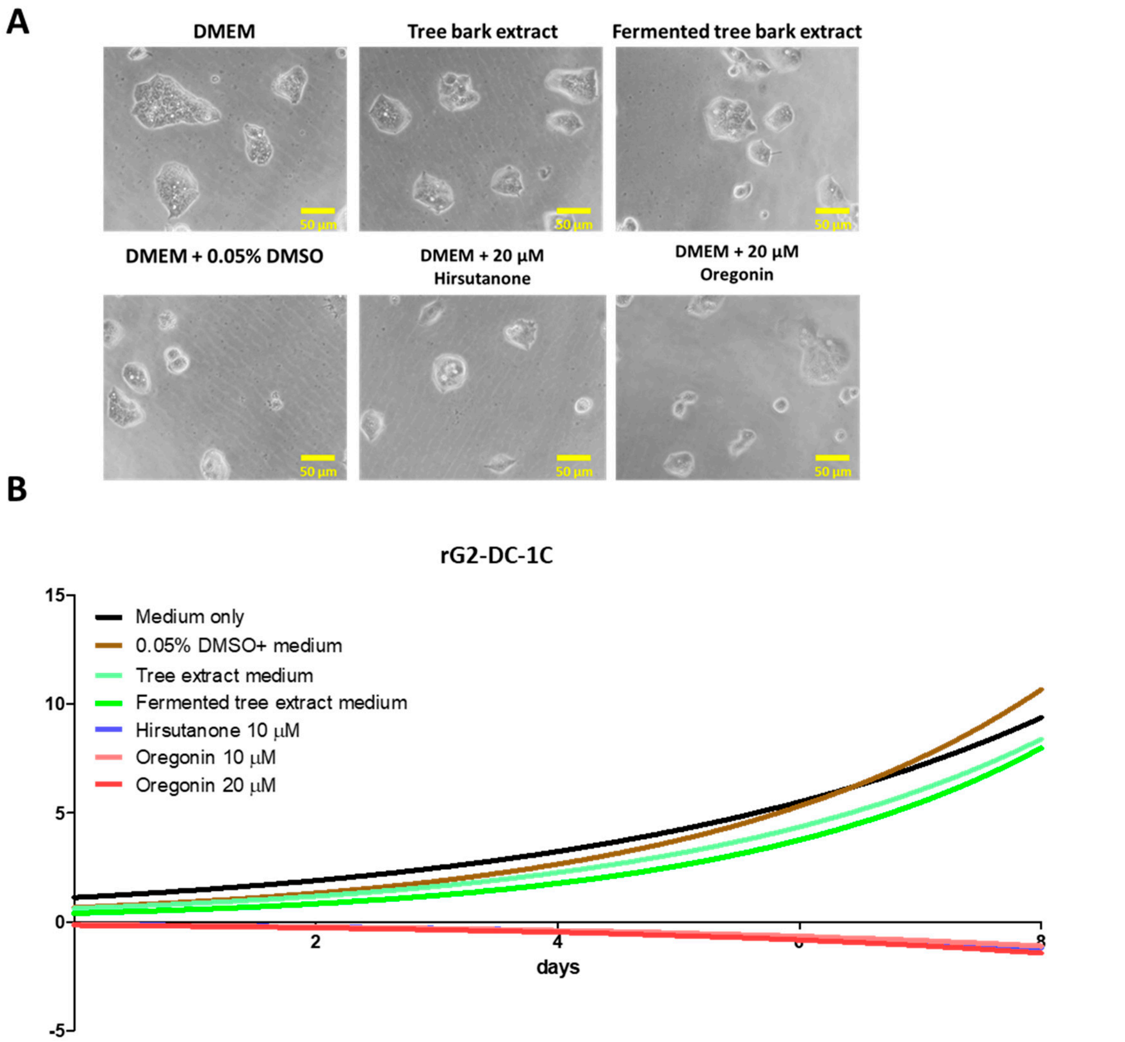

Figure S1. Microscopic morphology and cell proliferation/cytotoxicity of rG2-DC-1C cells after various treatments. (A) Microscopic morphologies of rG2-DC-1C cells after treatment with 0.05% DMSO, unfermented and fermented tree bark extracts, and 20 µM Hir and 20 µM Ore. (**B**) Cell viability was measured using MTT assay as described in Materials and methods. Cells (3×10^4) were exposed to unfermented and fermented tree bark extracts in culture medium and the indicated concentrations of Hir and Ore at 37 °C for the indicated time periods. The medium was then incubated with 10 µL of 10 mg/mL MTT solution for 2 h at 37 °C. After centrifugation for 5 min, the medium was removed, and 100 µL of DMSO was added to each well to dissolve the formazan. The

absorbance was then measured at 570 nm using a microplate reader. Cell viability over 8 days was determined relative to the control culture.

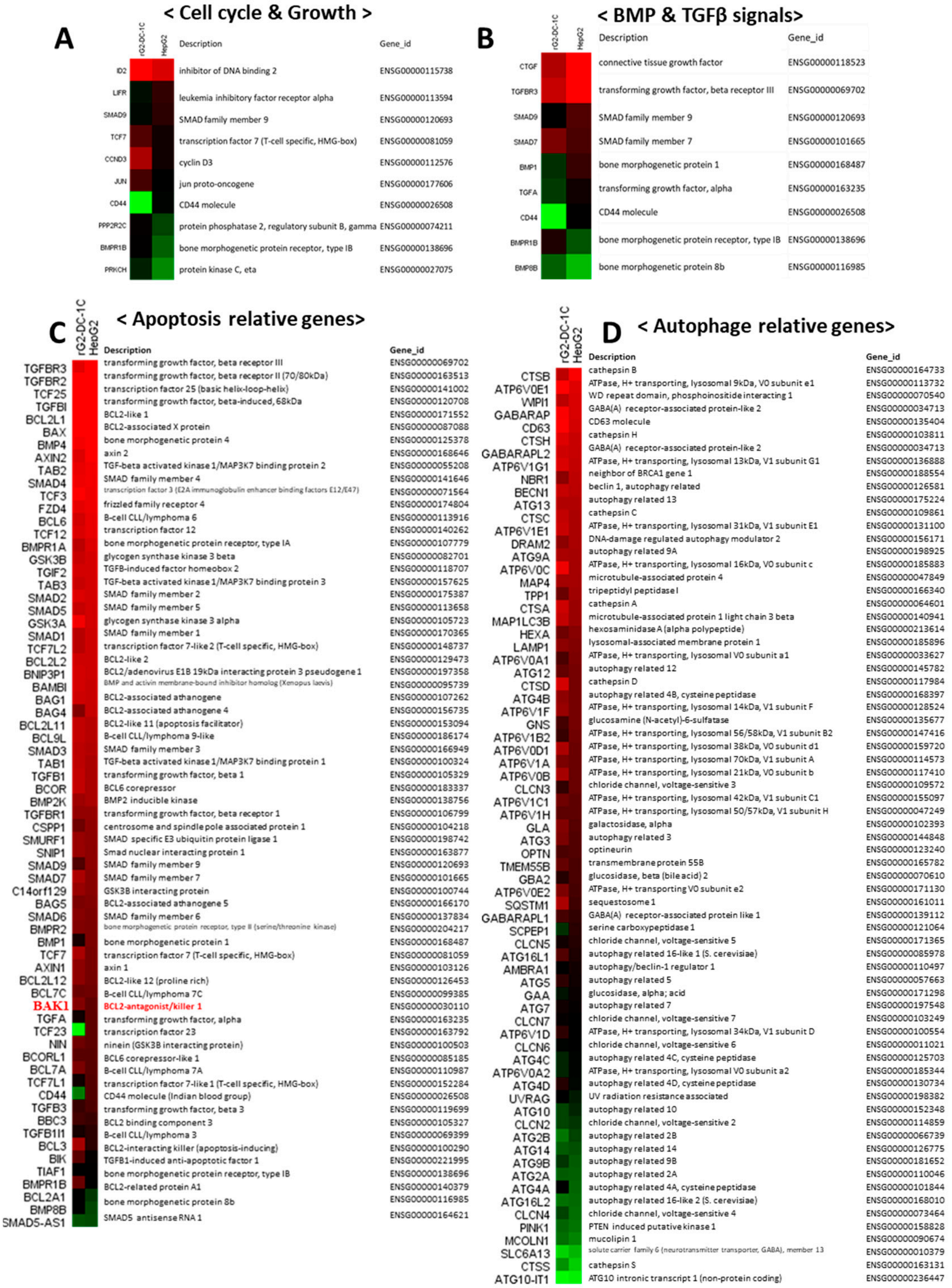
< Cell cycle & Growth >
A
rG2-DC-1C
HepG2
Description
Gene_id
ID2 inhibitor of DNA binding 2 ENSG00000115738
LIFR leukemia inhibitory factor receptor alpha ENSG00000113594
SMAD9 SMAD family member 9 ENSG00000120693
TCF7 transcription factor 7 (T-cell specific, HMG-box) ENSG00000081059
CCND3 cyclin D3 ENSG00000112576
JUN jun proto-oncogene ENSG00000177606
CD44 CD44 molecule ENSG00000026508
PPP2R2C protein phosphatase 2, regulatory subunit B, gamma ENSG00000074211
BMPR1B bone morphogenetic protein receptor, type IB ENSG00000138696
PRKCH protein kinase C, eta ENSG0000027075
< BMP & TGFβ signals>
B
rG2-DC-1C
HepG2
Description
Gene_id
CTGF connective tissue growth factor ENSG00000118523
TGFBR3 transforming growth factor, beta receptor III ENSG00000069702
SMAD9 SMAD family member 9 ENSG00000120693
SMAD7 SMAD family member 7 ENSG00000101665
BMP1 bone morphogenetic protein 1 ENSG00000168487
TGFA transforming growth factor, alpha ENSG00000163235
CD44 CD44 molecule ENSG00000026508
BMPR1B bone morphogenetic protein receptor, type IB ENSG00000138696
BMP8B bone morphogenetic protein 8b ENSG00000116985
< Apoptosis relative genes>
C
rG2-DC-1C
HepG2
Description
Gene_id
TGFBR3 transforming growth factor, beta receptor III ENSG00000069702
TGFBR2 transforming growth factor, beta receptor II (70/80kDa) ENSG00000163513
TCF25 transcription factor 25 (basic helix-loop-helix) ENSG00000141002
TGFBI transforming growth factor, beta-induced, 68kDa ENSG00000120708
BCL2L1 BCL2-like 1 ENSG00000171552
BAX BCL2-associated X protein ENSG00000087088
BMP4 bone morphogenetic protein 4 ENSG00000125378
AXIN2 axin 2 ENSG00000168646
TAB2 TGF-beta activated kinase 1/MAP3K7 binding protein 2 ENSG00000055208
SMAD4 SMAD family member 4 ENSG00000141646
TCF3 transcription factor 3 (E2A immunoglobulin enhancer binding factors E12/E47) ENSG00000071564
FZD4 frizzled family receptor 4 ENSG00000174804
BCL6 B-cell CLL/lymphoma 6 ENSG00000113916
TCF12 transcription factor 12 ENSG00000140262
BMPR1A bone morphogenetic protein receptor, type IA ENSG00000107779
GSK3B glycogen synthase kinase 3 beta ENSG00000082701
TGIF2 TGFB-induced factor homeobox 2 ENSG00000118707
TAB3 TGF-beta activated kinase 1/MAP3K7 binding protein 3 ENSG00000157625
SMAD2 SMAD family member 2 ENSG00000175387
SMAD5 SMAD family member 5 ENSG00000113658
GSK3A glycogen synthase kinase 3 alpha ENSG00000105723
SMAD1 SMAD family member 1 ENSG00000170365
TCF7L2 transcription factor 7-like 2 (T-cell specific, HMG-box) ENSG00000148737
BCL2L2 BCL2-like 2 ENSG00000129473
BNIP3P1 BCL2/adenovirus E1B 19kDa interacting protein 3 pseudogene 1 ENSG00000197358
BAMBI BMP and activin membrane-bound inhibitor homolog (Xenopus laevis) ENSG00000095739
BAG1 BCL2-associated athanogene ENSG00000107262
BAG4 BCL2-associated athanogene 4 ENSG00000156735
BCL2L11 BCL2-like 11 (apoptosis facilitator) ENSG00000153094
BCL9L B-cell CLL/lymphoma 9-like ENSG00000186174
SMAD3 SMAD family member 3 ENSG00000166949
TAB1 TGF-beta activated kinase 1/MAP3K7 binding protein 1 ENSG00000100324
TGFB1 transforming growth factor, beta 1 ENSG00000105329
BCOR BCL6 corepressor ENSG00000183337
BMP2K BMP2 inducible kinase ENSG00000138756
TGFBR1 transforming growth factor, beta receptor 1 ENSG00000106799
CSPP1 centrosome and spindle pole associated protein 1 ENSG00000104218
SMURF1 SMAD specific E3 ubiquitin protein ligase 1 ENSG00000198742
SNIP1 Smad nuclear interacting protein 1 ENSG00000163877
SMAD9 SMAD family member 9 ENSG00000120693
SMAD7 SMAD family member 7 ENSG00000101665
C14orf129 GSK3B interacting protein ENSG00000100744
BAG5 BCL2-associated athanogene 5 ENSG00000166170
SMAD6 SMAD family member 6 ENSG00000137834
BMPR2 bone morphogenetic protein receptor, type II (serine/threonine kinase) ENSG00000204217
BMP1 bone morphogenetic protein 1 ENSG00000168487
TCF7 transcription factor 7 (T-cell specific, HMG-box) ENSG00000081059
AXIN1 axin 1 ENSG00000103126
BCL2L12 BCL2-like 12 (proline rich) ENSG00000126453
BCL7C B-cell CLL/lymphoma 7C ENSG00000099385
BAK1 BCL2-antagonist/killer 1 ENSG00000030110
TGFA transforming growth factor, alpha ENSG00000163235
TCF23 transcription factor 23 ENSG00000163792
NIN ninein (GSK3B interacting protein) ENSG00000100503
BCORL1 BCL6 corepressor-like 1 ENSG00000085185
BCL7A B-cell CLL/lymphoma 7A ENSG00000110987
TCF7L1 transcription factor 7-like 1 (T-cell specific, HMG-box) ENSG00000152284
CD44 CD44 molecule (Indian blood group) ENSG00000026508
TGFB3 transforming growth factor, beta 3 ENSG00000119699
BBC3 BCL2 binding component 3 ENSG00000105327
TGFB1I1 B-cell CLL/lymphoma 3 ENSG00000069399
BCL3 BCL2-interacting killer (apoptosis-inducing) ENSG00000100290
BIK TGFB1-induced anti-apoptotic factor 1 ENSG00000221995
TIAF1 bone morphogenetic protein receptor, type IB ENSG00000138696
BMPR1B BCL2-related protein A1 ENSG00000140379
BCL2A1 bone morphogenetic protein 8b ENSG00000116985
BMP8B
SMAD5-AS1 SMAD5 antisense RNA 1 ENSG00000164621
< Autophage relative genes>
D
rG2-DC-1C
HepG2
Description
Gene_id
CTSB cathepsin B ENSG00000164733
ATP6V0E1 ATPase, H+ transporting, lysosomal 9kDa, V0 subunit e1 ENSG00000113732
WIPI1 WD repeat domain, phosphoinositide interacting 1 ENSG00000070540
GABARAP GABA(A) receptor-associated protein-like 2 ENSG00000034713
CD63 CD63 molecule ENSG00000135404
CTSH cathepsin H ENSG00000103811
GABARAPL2 GABA(A) receptor-associated protein-like 2 ENSG00000034713
ATP6V1G1 ATPase, H+ transporting, lysosomal 13kDa, V1 subunit G1 ENSG00000136888
NBR1 neighbor of BRCA1 gene 1 ENSG00000188554
BECN1 beclin 1, autophagy related ENSG00000126581
ATG13 autophagy related 13 ENSG00000175224
CTSC cathepsin C ENSG00000109861
ATP6V1E1 ATPase, H+ transporting, lysosomal 31kDa, V1 subunit E1 ENSG00000131100
DRAM2 DNA-damage regulated autophagy modulator 2 ENSG00000156171
ATG9A autophagy related 9A ENSG00000198925
ATP6V0C ATPase, H+ transporting, lysosomal 16kDa, V0 subunit c ENSG00000185883
MAP4 microtubule-associated protein 4 ENSG00000047849
TPP1 tripeptidyl peptidase I ENSG00000166340
CTSA cathepsin A ENSG00000064601
MAP1LC3B microtubule-associated protein 1 light chain 3 beta ENSG00000140941
HEXA hexosaminidase A (alpha polypeptide) ENSG00000213614
LAMP1 lysosomal-associated membrane protein 1 ENSG00000185896
ATP6V0A1 ATPase, H+ transporting, lysosomal V0 subunit a1 ENSG00000033627
ATG12 autophagy related 12 ENSG00000145782
CTSD cathepsin D ENSG00000117984
ATG4B autophagy related 4B, cysteine peptidase ENSG00000168397
ATP6V1F ATPase, H+ transporting, lysosomal 14kDa, V1 subunit F ENSG00000128524
GNS glucosamine (N-acetyl)-6-sulfatase ENSG00000135677
ATP6V1B2 ATPase, H+ transporting, lysosomal 56/58kDa, V1 subunit B2 ENSG00000147416
ATP6V0D1 ATPase, H+ transporting, lysosomal 38kDa, V0 subunit d1 ENSG00000159720
ATP6V1A ATPase, H+ transporting, lysosomal 70kDa, V1 subunit A ENSG00000114573
ATP6V0B ATPase, H+ transporting, lysosomal 21kDa, V0 subunit b ENSG00000117410
CLCN3 chloride channel, voltage-sensitive 3 ENSG00000109572
ATP6V1C1 ATPase, H+ transporting, lysosomal 42kDa, V1 subunit C1 ENSG00000155097
ATP6V1H ATPase, H+ transporting, lysosomal 50/57kDa, V1 subunit H ENSG00000047249
GLA galactosidase, alpha ENSG00000102393
ATG3 autophagy related 3 ENSG00000144848
OPTN optineurin ENSG00000123240
TMEM55B transmembrane protein 55B ENSG00000165782
GBA2 glucosidase, beta (bile acid) 2 ENSG00000070610
ATP6V0E2 ATPase, H+ transporting V0 subunit e2 ENSG00000171130
SQSTM1 sequestosome 1 ENSG00000161011
GABARAPL1 GABA(A) receptor-associated protein like 1 ENSG00000139112
SCPEP1 serine carboxypeptidase 1 ENSG00000121064
CLCN5 chloride channel, voltage-sensitive 5 ENSG00000171365
ATG16L1 autophagy related 16-like 1 (S. cerevisiae) ENSG00000085978
AMBRA1 autophagy/beclin-1 regulator 1 ENSG00000110497
ATG5 autophagy related 5 ENSG00000057663
GAA glucosidase, alpha; acid ENSG00000171298
ATG7 autophagy related 7 ENSG00000197548
CLCN7 chloride channel, voltage-sensitive 7 ENSG00000103249
ATP6V1D ATPase, H+ transporting, lysosomal 34kDa, V1 subunit D ENSG00000100554
CLCN6 chloride channel, voltage-sensitive 6 ENSG00000011021
ATG4C autophagy related 4C, cysteine peptidase ENSG00000125703
ATP6V0A2 ATPase, H+ transporting, lysosomal V0 subunit a2 ENSG00000185344
ATG4D autophagy related 4D, cysteine peptidase ENSG00000130734
UVRAG UV radiation resistance associated ENSG00000198382
ATG10 autophagy related 10 ENSG00000152348
CLCN2 chloride channel, voltage-sensitive 2 ENSG00000114859
ATG2B autophagy related 2B ENSG00000066739
ATG14 autophagy related 14 ENSG00000126775
ATG9B autophagy related 9B ENSG00000181652
ATG2A autophagy related 2A ENSG00000110046
ATG4A autophagy related 4A, cysteine peptidase ENSG00000101844
ATG16L2 autophagy related 16-like 2 (S. cerevisiae) ENSG00000168010
CLCN4 chloride channel, voltage-sensitive 4 ENSG00000073464
PINK1 PTEN induced putative kinase 1 ENSG00000158828
MCOLN1 mucolipin 1 ENSG00000090674
SLC6A13 solute carrier family 6 (neurotransmitter transporter, GABA), member 13 ENSG00000010379
CTSS cathepsin S ENSG00000163131
ATG10-IT1 ATG10 intronic transcript 1 (non-protein coding) ENSG00000236447

Figure S2. Comparative analysis between rG2-DC-1C and HepG2 cells. (**A**) Heatmap of the mRNA expression levels for select genes related to cell cycle and proliferation in rG2-DC-1C and HepG2 cells, as determined by RNA sequencing as described in Materials and methods. The number of upregulated (4.0-fold) and downregulated (0.25-fold) genes is indicated. (**B**) Heatmap of mRNA expression levels for BMP and TGF-β signaling genes in rG2-DC-1C and HepG2 cells, as determined by RNA sequencing. The number of upregulated (4.0-fold) and downregulated (0.25-fold) genes is indicated. (**C**) Comparison of mRNAs encoding apoptosis-related genes in rG2-DC-1C and HepG2 cells, as determined by RNA sequencing. (**D**) Comparison of mRNAs of autophagy-related genes in rG2-DC-1C and HepG2 cells, as determined by RNA sequencing. RNA sequencing data were deposited in the NCBI Bioproject Database (http://www.ncbi.nlm.nih.gov/bioproject) using the accession number PRJNA273617.

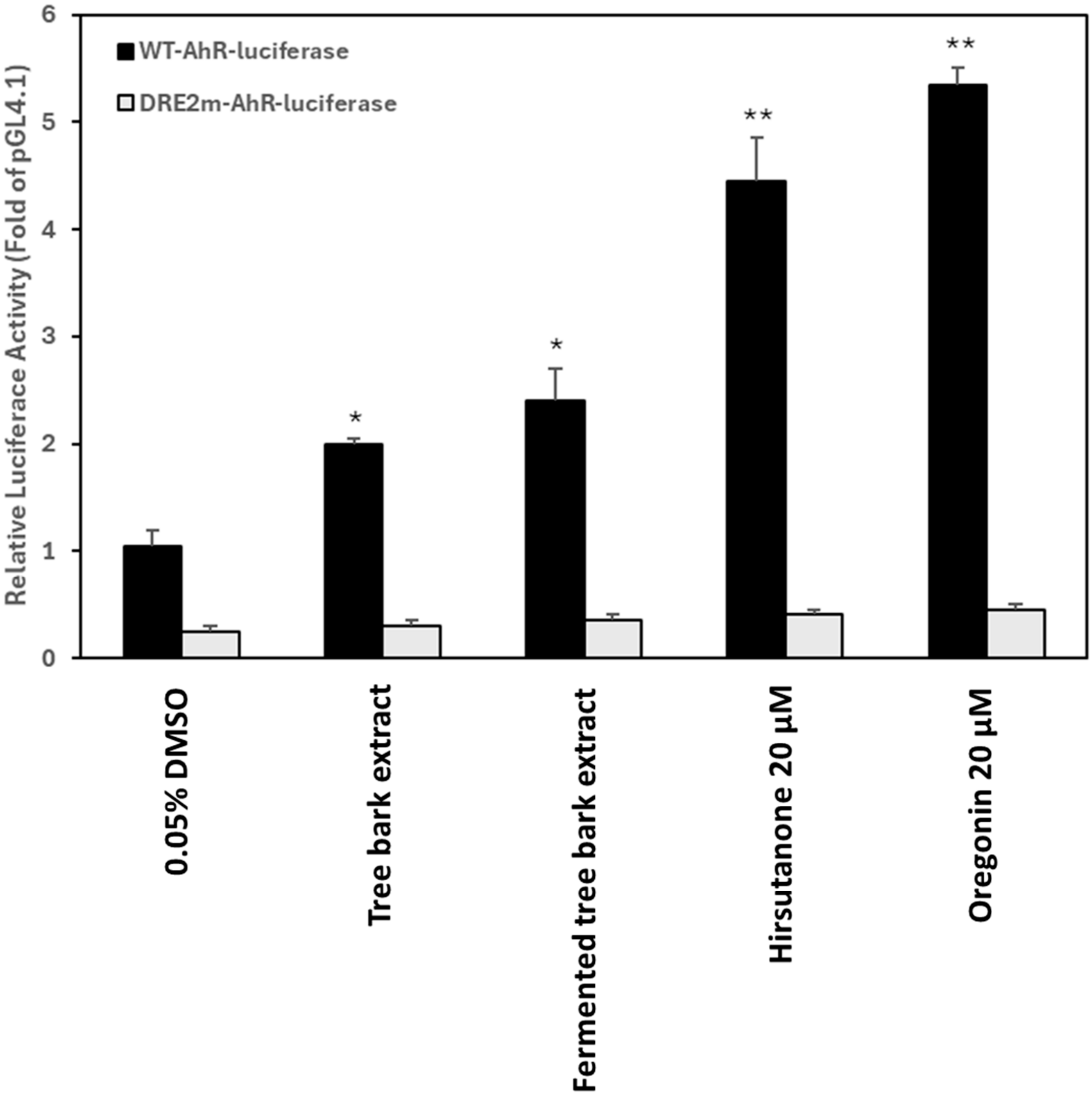

Figure S3. Relative AHR-luciferase activities in wild-type and BRD2 mutant constructs in rG2-DC-1C cells. Comparative luciferase activity of pGL4.1-AhR luciferase and DRE2 mutated pGL4.1 DRE2m-AhR luciferase was calculated in rG2-DC-1C cells. 0.05% DMSO addition was arbitrarily set at 1.0. Data were analyzed using one-way ANOVA with Tukey's post hoc test (* $p < 0.05$ and ** $p < 0.01$, n = 5).

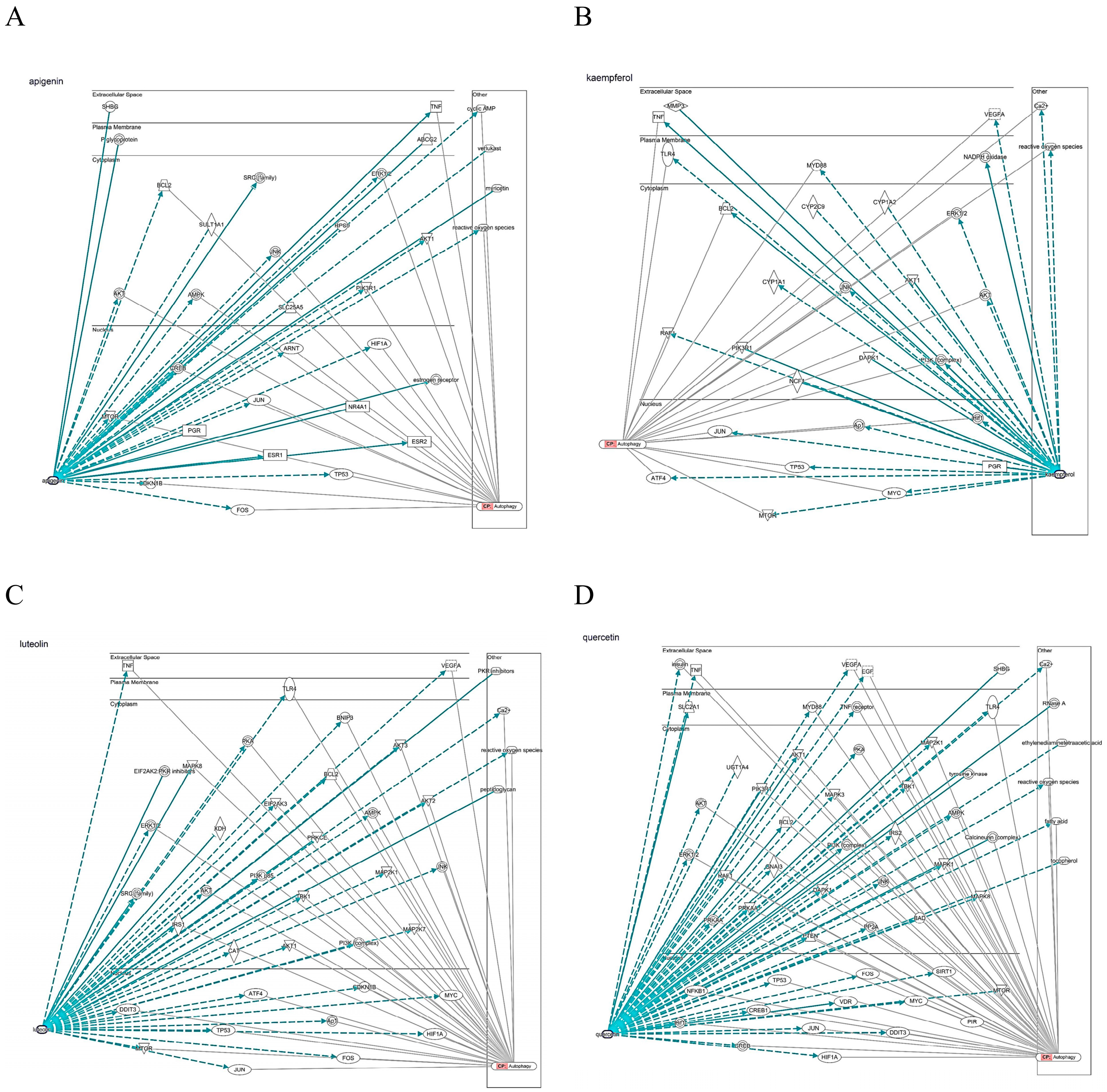
A
apigenin
Extracellular Space
Plasma Membrane
Cytoplasm
Nucleus
Other
B
kaempferol
Extracellular Space
Plasma Membrane
Cytoplasm
Nucleus
Other
C
luteolin
Extracellular Space
Plasma Membrane
Cytoplasm
Other
D
quercetin
Extracellular Space
Plasma Membrane
Cytoplasm
Other

E

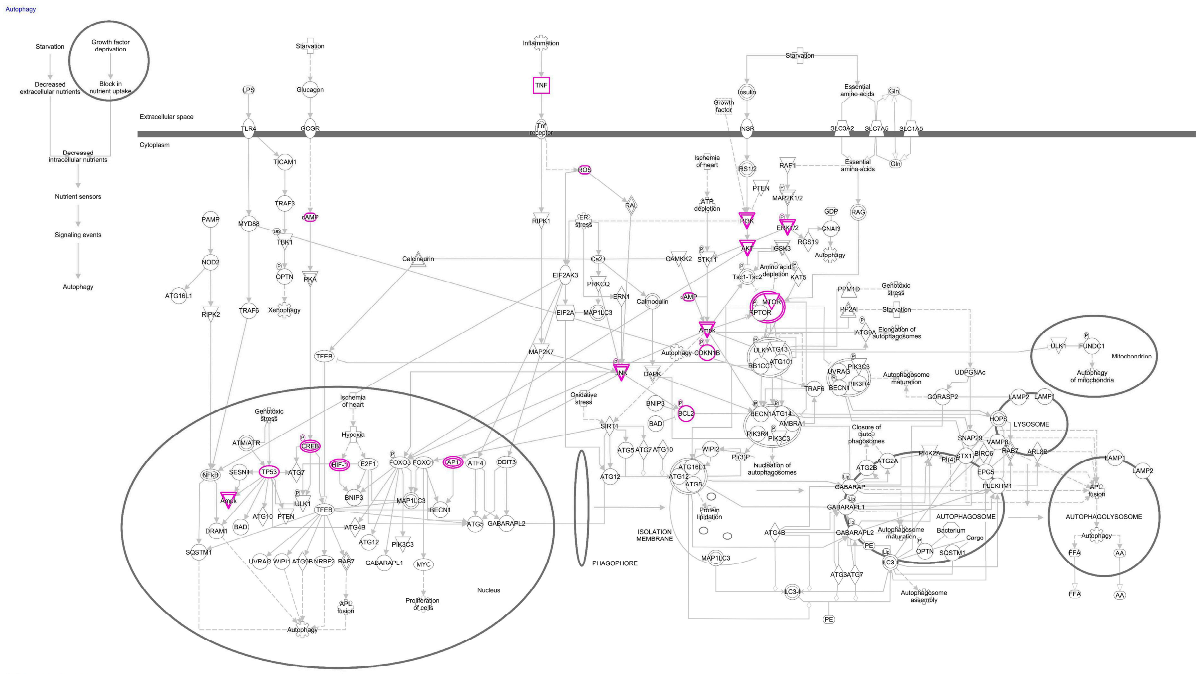

© 2000-2025 QIAGEN. All rights reserved.

F

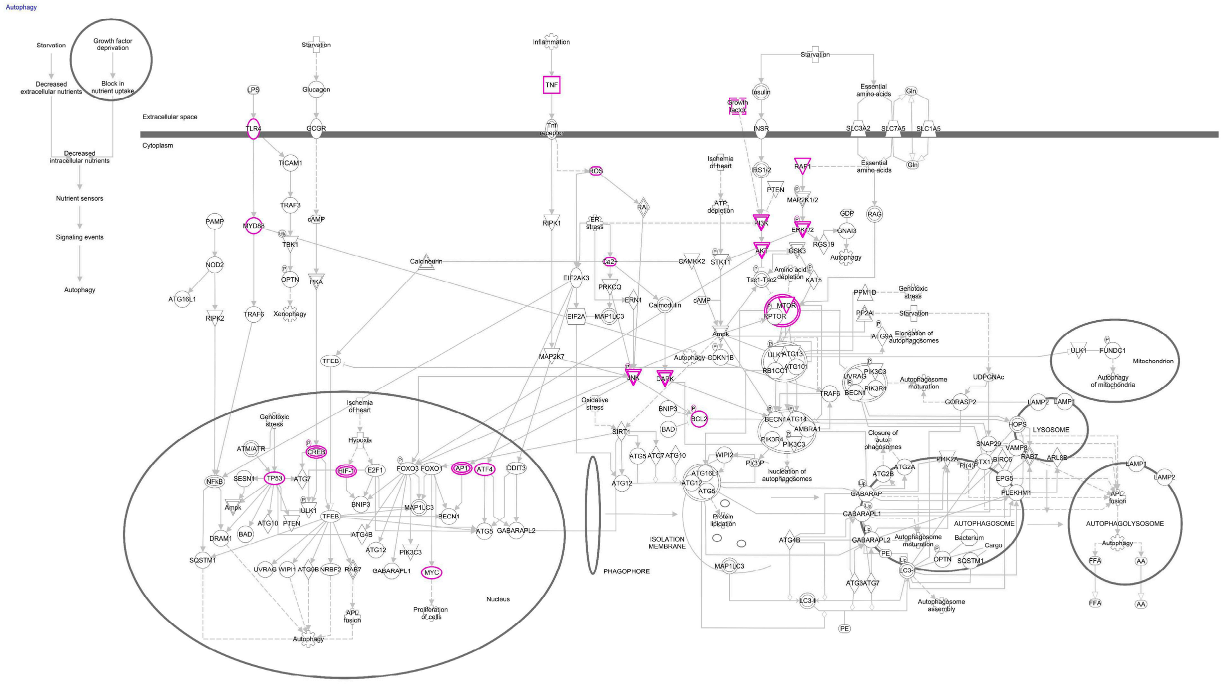

© 2000-2025 QIAGEN. All rights reserved.

G

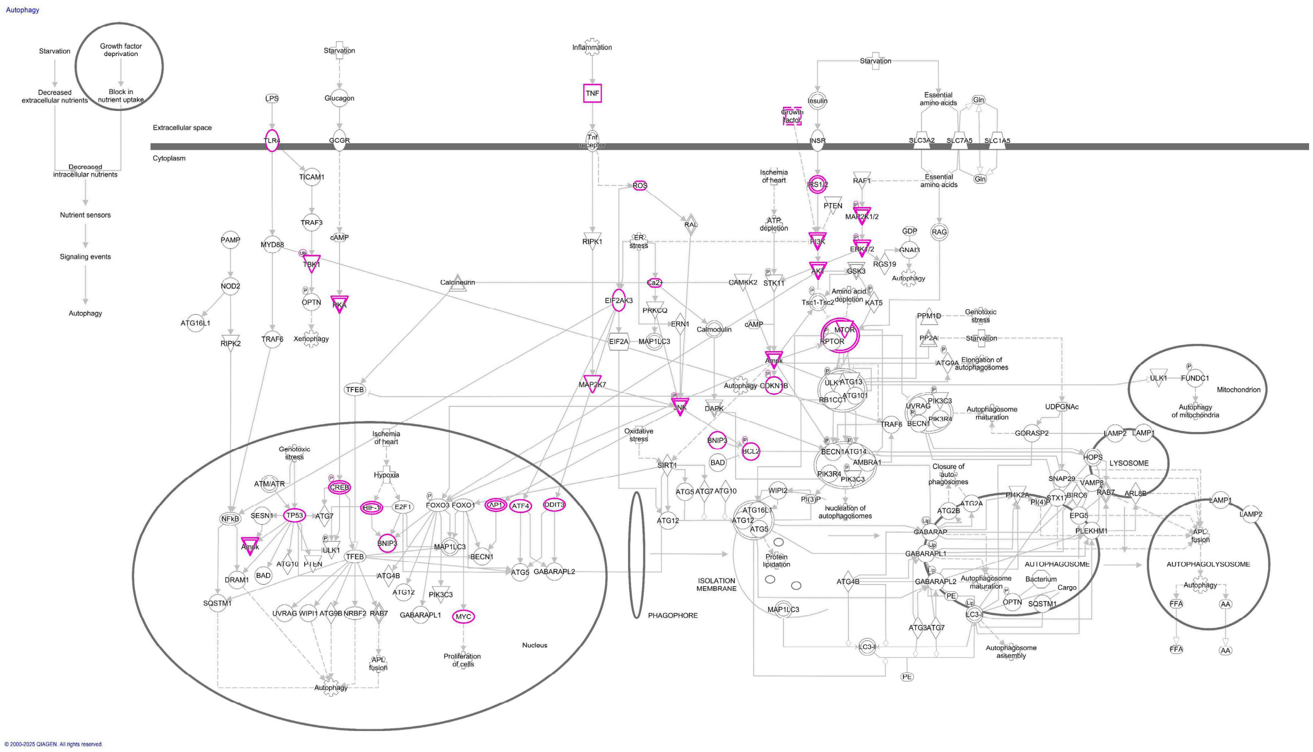
Autophagy
Starvation
Growth factor deprivation
Decreased extracellular nutrients
Block in nutrient uptake
Decreased intracellular nutrients
Nutrient sensors
Signaling events
Autophagy
LPS
Glucagon
Inflammation
TNF
Starvation
Insulin
Essential amino acids
Gln
Extracellular space
Cytoplasm
TLR4
GCGR
INSR
SLC3A2
SLC7A5
SLC1A5
TICAM1
TRAF3
cAMP
IRS1/2
PTEN
RAF1
MAP2K1/2
ROS
RAL
Ischemia of heart
ATP depletion
PI3K
ERK1/2
GDP
GNAI3
RAG
RGS19
PAMP
MYD88
TBK1
NOD2
OPTN
PKA
Calcineurin
RIPK1
ER stress
Ca2+
CAMKK2
STK11
AKT
GSK3
Autophagy
Amino acid depletion
Tsc1-Tsc2
KAT5
EIF2AK3
PRKCQ
ERN1
Calmodulin
cAMP
ATG16L1
RIPK2
TRAF6
Xenophagy
EIF2A
MAP1LC3
MTOR
RPTOR
PPM1D
Genotoxic stress
PP2A
Starvation
ATG9A
Elongation of autophagosomes
Ampk
ULK1
FUNDC1
Mitochondrion
Autophagy of mitochondria
TFEB
MAP2K7
Autophagy
CDKN1B
ATG13
ATG101
RB1CC1
UVRAG
PIK3C3
PIK3R4
BECN1
Autophagosome maturation
UDPGNAc
TRAF6
JNK
DAPK
GORASP2
LAMP2
LAMP1
Ischemia of heart
Oxidative stress
BNIP3
BCL2
BAD
HOPS
LYSOSOME
Genotoxic stress
Hypoxia
SIRT1
BECN1
ATG14
AMBRA1
PIK3R4
PIK3C3
Closure of auto-phagosomes
ATM/ATR
CREB
WIPI2
PI(3)P
SNAP29
VAMP8
RAB7
ARL8B
NFkB
SESN1
TP53
ATG7
HIF-1
E2F1
FOXO3
FOXO1
AP1
ATF4
DDIT3
ATG5
ATG7
ATG10
ATG12
ATG16L1
ATG12
ATG5
Nucleation of autophagosomes
PI4K2A
PI(4)P
STX17
BIRC6
EPG5
PLEKHM1
ATG2B
ATG2A
GABARAP
GABARAPL1
LAMP1
LAMP2
APL fusion
AUTOPHAGOSOME
AUTOPHAGOLYSOSOME
Autophagy
Ampk
ULK1
BNIP3
DRAM1
BAD
ATG10
PTEN
TFEB
MAP1LC3
BECN1
ATG4B
ATG12
PIK3C3
ATG5
GABARAPL2
Protein lipidation
ISOLATION MEMBRANE
ATG4B
GABARAPL2
Autophagosome maturation
Bacterium
Cargo
PE
OPTN
SQSTM1
SQSTM1
UVRAG
WIPI1
ATG9B
NRBF2
RAB7
GABARAPL1
MYC
PHAGOPHORE
MAP1LC3
LC3-I
LC3-II
ATG3
ATG7
Autophagosome assembly
FFA
AA
FFA
AA
Nucleus
APL fusion
Proliferation of cells
Autophagy
PE
© 2000-2025 QIAGEN. All rights reserved.

H

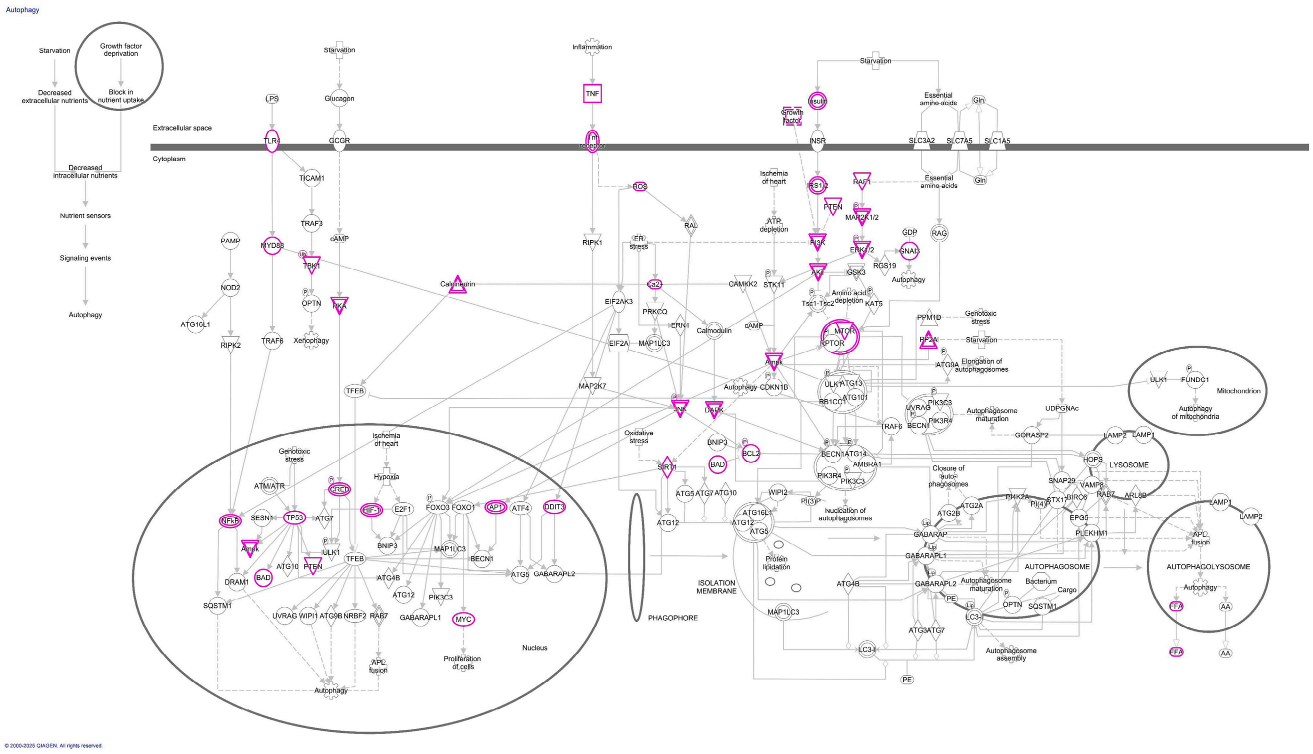
Autophagy
Starvation
Growth factor deprivation
Decreased extracellular nutrients
Block in nutrient uptake
Decreased intracellular nutrients
Nutrient sensors
Signaling events
Autophagy
LPS
Glucagon
Inflammation
TNF
Starvation
Insulin
Growth factor
Essential amino acids
Gln
Extracellular space
Cytoplasm
TLR4
GCGR
INSR
SLC3A2
SLC7A5
SLC1A5
TICAM1
TRAF3
cAMP
IRS1/2
PTEN
RAF1
MAP2K1/2
ROS
RAL
Ischemia of heart
ATP depletion
PI3K
ERK1/2
GDP
GNAI3
RAG
RGS19
PAMP
MYD88
TBK1
NOD2
OPTN
PKA
Calcineurin
RIPK1
ER stress
Ca2+
CAMKK2
STK11
AKT
GSK3
Autophagy
Amino acid depletion
Tsc1-Tsc2
KAT5
EIF2AK3
PRKCQ
ERN1
Calmodulin
cAMP
ATG16L1
RIPK2
TRAF6
Xenophagy
EIF2A
MAP1LC3
MTOR
RPTOR
PPM1D
Genotoxic stress
PP2A
Starvation
ATG9A
Elongation of autophagosomes
Ampk
ULK1
FUNDC1
Mitochondrion
Autophagy of mitochondria
TFEB
MAP2K7
Autophagy
CDKN1B
ATG13
ATG101
RB1CC1
UVRAG
PIK3C3
PIK3R4
BECN1
Autophagosome maturation
UDPGNAc
TRAF6
JNK
DAPK
GORASP2
LAMP2
LAMP1
Ischemia of heart
Oxidative stress
BNIP3
BCL2
BAD
HOPS
LYSOSOME
Genotoxic stress
Hypoxia
SIRT1
BECN1
ATG14
AMBRA1
PIK3R4
PIK3C3
Closure of auto-phagosomes
ATM/ATR
CREB
WIPI2
PI(3)P
SNAP29
VAMP8
RAB7
ARL8B
NFkB
SESN1
TP53
ATG7
HIF-1
E2F1
FOXO3
FOXO1
AP1
ATF4
DDIT3
ATG5
ATG7
ATG10
ATG12
ATG16L1
ATG12
ATG5
Nucleation of autophagosomes
PI4K2A
PI(4)P
STX17
BIRC6
EPG5
PLEKHM1
ATG2B
ATG2A
GABARAP
GABARAPL1
LAMP1
LAMP2
APL fusion
AUTOPHAGOSOME
AUTOPHAGOLYSOSOME
Autophagy
Ampk
ULK1
BNIP3
DRAM1
BAD
ATG10
PTEN
TFEB
MAP1LC3
BECN1
ATG4B
ATG12
PIK3C3
ATG5
GABARAPL2
Protein lipidation
ISOLATION MEMBRANE
ATG4B
GABARAPL2
Autophagosome maturation
Bacterium
Cargo
PE
OPTN
SQSTM1
SQSTM1
UVRAG
WIPI1
ATG9B
NRBF2
RAB7
GABARAPL1
MYC
PHAGOPHORE
MAP1LC3
LC3-I
LC3-II
ATG3
ATG7
Autophagosome assembly
FFA
AA
FFA
AA
Nucleus
APL fusion
Proliferation of cells
Autophagy
PE
© 2000-2025 QIAGEN. All rights reserved.

Figure S4. Ingenuity pathway analysis (QIAGEN Inc. released Nov. 4, 2025) was used to identify the network of extract components and the molecules of autophagy pathways. These targeted network molecules were matched to the molecules in the autophagy signaling pathway separately for each flavonoid using IPA. (**A**) Apigenin, (**B**) luteolin, (**C**) kaempferol, and (**D**) quercetin. After comparison, only the molecules common to these four networks and the autophagy signaling pathway were retained in the final network. Based on this result, these common molecules were also marked in the canonical autophagy signaling pathway using different colors. (**E**) Apigenin, (**F**) luteolin, (**G**) kaempferol, and (**H**) quercetin.

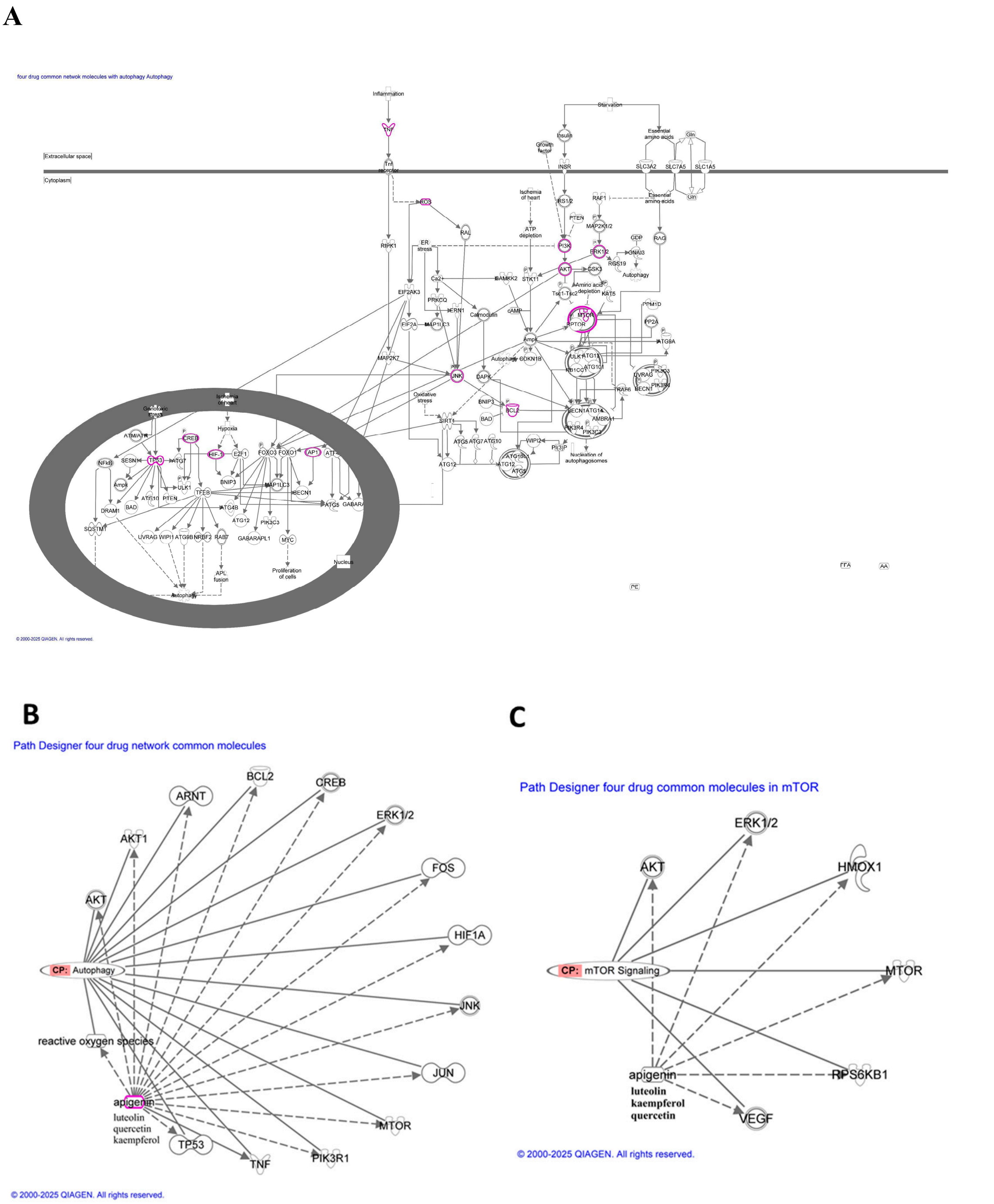

Figure S5. The proposed common network signaling of four flavonoids (apigenin, luteolin, kaempferol, and quercetin) and the autophagy pathway. The targeted network molecules identified by IPA were also linked to molecules in the autophagy signaling pathway, separately for each flavonoid. The common molecules were also

marked in the canonical autophagy signaling pathway. **(A)** Shared common pathway molecules in the signaling related to autophagy and the four flavonoids. (**B**) Schematic model of the signaling pathways common to these four flavonoids and autophagy. AKT, RNT, BCL2, CREB. ERK2, FOS, HIF1A, JNK, JUN, MTOR, PIK3R1, TNF, and TP53 were identified. (**C**) The common molecules identified across four networks were mapped to the mTOR signaling pathway.

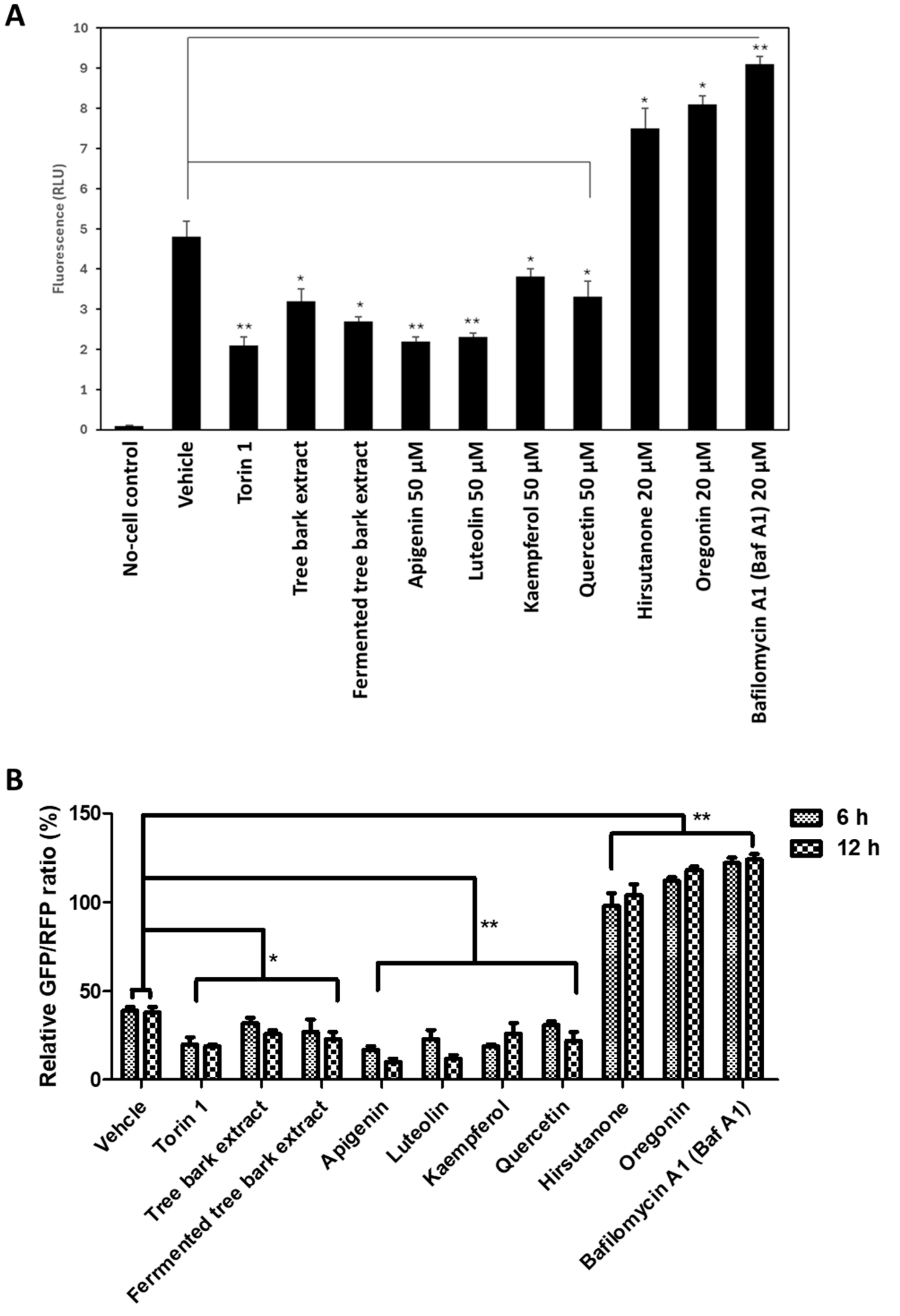

Figure S6. **Light chain 3 HiBiT receptor assays and relative GFP/RFP ration value of autophagy reaction using apigenin, luteolin, kaempferol, and quercetin.** Each

component was examined for autophagic activity using the Autophagy LC3 HiBiT Reporter Assay System. (**A**) U2OS HiBiT-HaloTag-LC3 cells were cultivated in Dulbecco's Modified Eagle Medium (high glucose) supplemented with 10% fetal bovine serum, 100 U/mL penicillin, and 100 U/mL streptomycin (8×10^3 cells per 80 µL in each well) in 96-well white clear-bottom plates. The indicated components (50 µM) in the unfermented or fermented tree bark extract from *A. japonica* and 20 µM Hir or 20 µM Ore were then examined for autophagic activity using this screening kit, as described in Methos and Materials. Data are presented as the mean ± SEM (n = 3). Data was analyzed using one-way ANOVA with Tukey's post hoc test (* $p < 0.05$ and ** $p < 0.01$). (**B**) rG2-DC-1C cells stably expressing pMRX-IP-GFP-LC3-RFP-LC3ΔG were cultured with different inducers and each of the four flavonoids (50 µM) and unfermented or fermented tree bark extracts, and 20 µM Hir or 20 µM Ore for 24 h. Then the GFP/RFP fluorescence ratio of the treated cells was expressed as a percentage relative to that of nontreated cells. Data represent mean ± SEM (n = 3). Bafilomycin A1 (BafA1); Torin1, mTOR inhibitor. Data are presented as the mean ± SEM (n = 3). Data was analyzed using one-way ANOVA with Tukey's post hoc test (* $p < 0.05$ and ** $p < 0.01$).

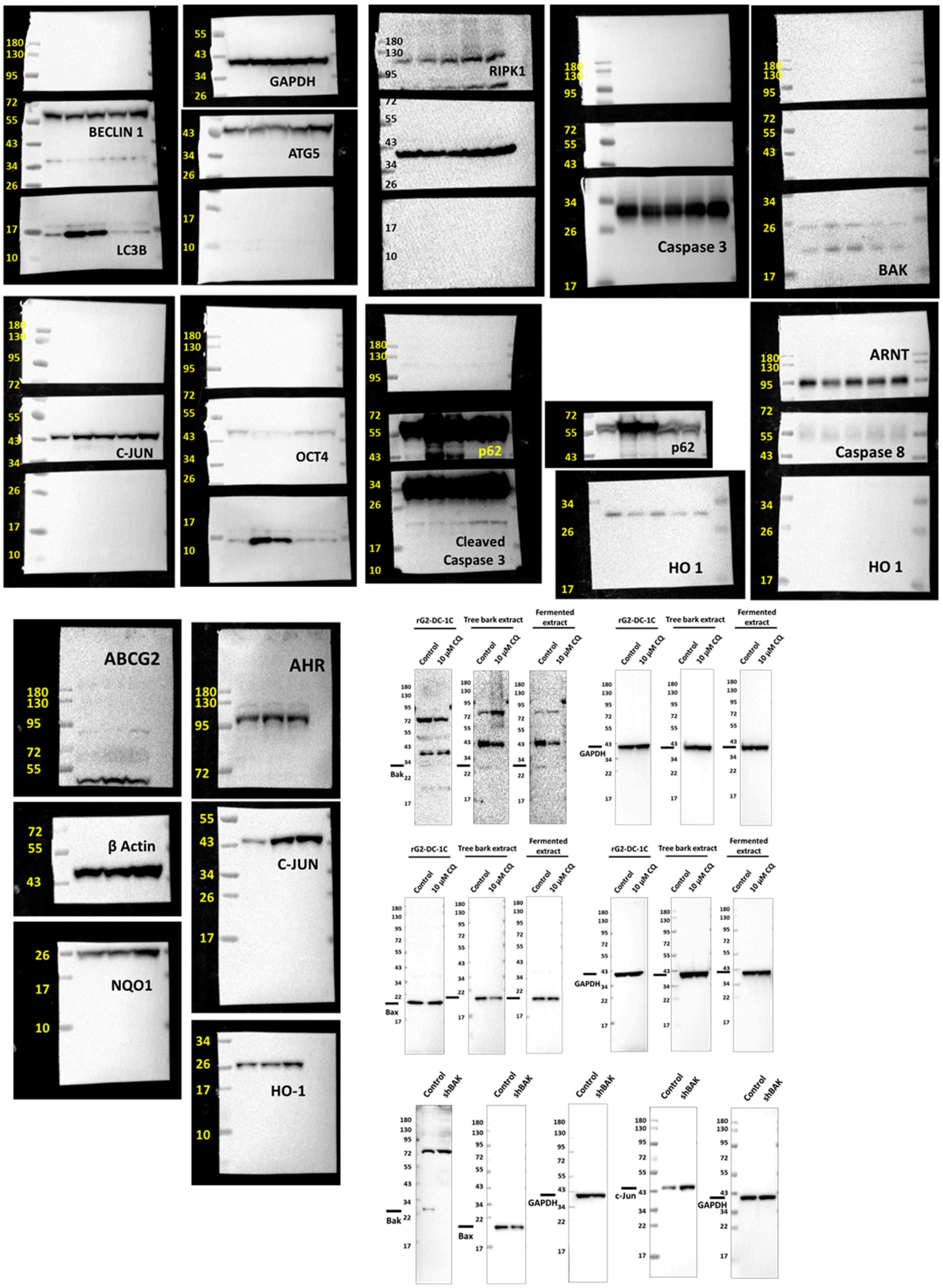

Figure S7. Full-length western blotting is used in this study.

Uncropped full length Western Blots are shown in these figure panels. The molecular weight markers are also listed.

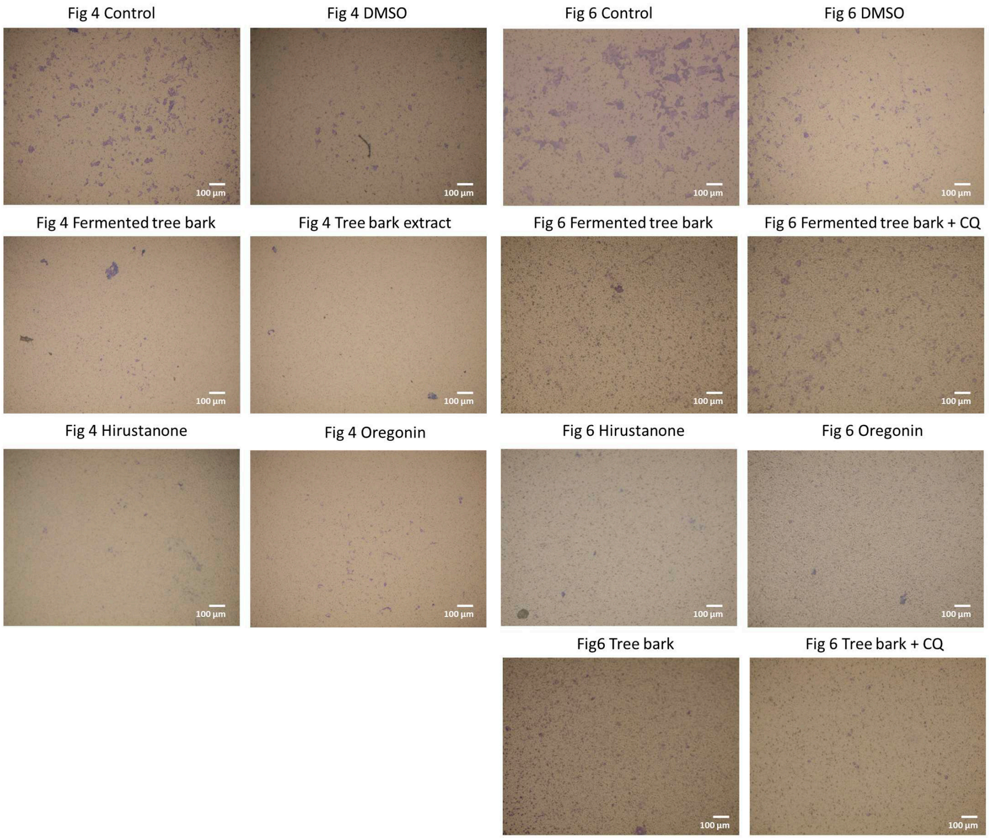

Figure S8. Bright view images taken in this study.

Original images are shown in figure 4 and figure 6 panels, the images were taken with 100x magnification under bright field.

Supplementary Table S1. Chemical constituents of the tree bark extract from *Alnus japonica*. Several candidate compounds derived from the bark were identified and reported by Sati et al. [18]. The major components were listed in this Table. Among these, we focused on four components such as apigenin, luteolin, kaempferol, and quercetin which have been previously implicated in induction of autophagy and apoptosis [45-50]. This Table lists the shared components detected in the non-fermented tree bark extracts and the unique composition of the fermented extract has not yet been fully characterized. Preliminary data suggest that fermentation alters the relative abundance of certain polyphenols, which may contribute to the observed differences in biological activity.

**1.**	**Lutein 7,4'-dimethyl ether (Pillion)**
**2.**	**Scutellarein-6,7,4'-trimethyl ether (salvigenin)**
**3.**	**Kaempferide**
**4.**	**Isohamnectin**
**5.**	**Rhamnazin**
**6.**	**Quercetin-7, 3', 4'-trimethyl ether**
**7.**	**6-hydoxyl-kaempferol-3,6, 4'-trimethyl ether**
**8.**	**Acacetin**
**9.**	**Apigenin-7,4'-dimethyl ether**
**10.**	**Scutellarein-6,4'-dimethyl ether**
**11.**	**Methyl (24-E)-3,4-secodammara-4 (28),20,24-trien-26-oic acid 3-oate**
**12.**	**Hirsutanonol 5-O-(6-O-galloyl)-β-D-glucopyranoside**
**13.**	**3-deoxo-hisutanonol 5-O-β-D-glucopyranoside**
**14.**	**Hirsutanonol-5-O-β-D-glucopyranoside**
**15.**	**Hirsutanone**
**16.**	**Oregonin**
**17.**	**β-amyrin**
**18.**	**3-O-acetyl-β-amyrin**
**19.**	**3-O-acetyltertaxerol**
**20.**	**Glutinane**
**21.**	**Lupenone**

**22.**	**Quercetin**
**23.**	**5-O-methyl hirsutanonol**
**24.**	**Glutinel**
**25.**	**Taraxrone**
**26.**	**Alnus japoninsA**
**27.**	**Alnus japonins B**
**28.**	**5-O-galloyl-shikimic acid**
**29.**	**4,6-di-O-galloyl-D-glucose**
**30.**	**1,4.-di-O-galloyl-β-D-glucose**
**31.**	**Strictinin**
**32.**	**Gemin D**
**33.**	**Pedunculagin**
**34.**	**Praecoxin A**
**35.**	**Flosin A**
**36.**	**Stachyurin**
**37.**	**Casuarinin**
**38.**	**Platyphylloside**
**39.**	**Garugamblin-3**
**40.**	**Acerogenin L**
**41.**	**Oregonoyl A**
**42.**	**Oregonoyl B**
**43.**	**Platyphyllme**
**44.**	**Platyphyllonol 5'-xylopyranpside**

References

1. Pokharel, A.; Mirza, B.S.; Dawson, J.O.; Hahn, D. Frankia populations in soil and root nodules of sympatrically grown Alnus taxa. *Microb Ecol* **2011**, *61*, 92-100, doi:10.1007/s00248-010-9726-2.
2. Lee, C.J.; Lee, S.S.; Chen, S.C.; Ho, F.M.; Lin, W.W. Oregonin inhibits lipopolysaccharide-induced iNOS gene transcription and upregulates HO-1 expression in macrophages and microglia. *Br J Pharmacol* **2005**, *146*, 378-388, doi:10.1038/sj.bjp.0706336.

3. Wada, H.; Tachibana, H.; Fuchino, H.; Tanaka, N. Three New Diarylheptanoid Glycosides from Alnus japonica. *CHEMICAL & PHARMACEUTICAL BULLETIN* **1998**, *46*, 1054-1055, doi:10.1248/cpb.46.1054.
4. Nomura, M.; Tokoroyama, T.; Kubota, T. Biarylheptanoids and other constituents from wood of *Alnus japonica*. *Phytochemistry* **1981**, *20*, 1097-1104, doi:https://doi.org/10.1016/0031-9422(81)83035-X.
5. Schwintzer, C.R.; Tjepkema, J.D. *The Biology of Frankia and Actinorhizal Plants*; Elsevier Science: 1990.
6. Gushiken, S. Japan Platform for Patent Information, No. 3803120. Available online: https://patentimages.storage.googleapis.com/21/5d/8d/fa2755968142ce/JP3803120B2.pdf (accessed on 7/18).
7. Mori, N. *Alnus japonica in Development of novel antiviral agents, Outline of research project, Microbiology/Oncology.*; University of the Ryukyus.: 2007; p. 186.
8. Nerome, K. Japan Platform for Patent Information, No. WO2010/005010A1. Available online: https://patents.google.com/patent/WO2010005010A1/ja (accessed on 7/18).
9. Nagayama, T. *Alnus japonica fermented extract*; Drinker testimonial materials (Japanese), 2021; Volume 2021.
10. GUSHIKEN, S.; Shimoji, Y. Alder deodorant. Available online: https://www.inpit.go.jp/blob/katsuyo/pdf/business/19t1-4.pdf (accessed on 7/19).
11. Okinawa Eco-Science Co., L. Hannoki hakkou ekisu no kouka nituite (Japanese). Inyousya taiken siryou: 2021; pp. 1-7.
12. Uto, T.; Tung, N.H.; Shoyama, Y. Hirsutanone Isolated from the Bark of Alnus japonica Attenuates Melanogenesis via Dual Inhibition of Tyrosinase Activity and Expression of Melanogenic Proteins. *Plants (Basel)* **2022**, *11*, doi:10.3390/plants11141875.
13. Dong, G.Z.; Jeong, J.H.; Lee, Y.I.; Han, Y.E.; Shin, J.S.; Kim, Y.J.; Jeon, R.; Kim, Y.H.; Park, T.J.; Kim, K.I.; et al. A lignan induces lysosomal dependent degradation of FoxM1 protein to suppress beta-catenin nuclear translocation. *Sci Rep* **2017**, *7*, 45951, doi:10.1038/srep45951.
14. Kim, H.J.; Yeom, S.H.; Kim, M.K.; Shim, J.G.; Paek, I.N.; Lee, M.W. Nitric oxide and prostaglandin E2 synthesis inhibitory activities of diarylheptanoids from the barks of Alnus japonica steudel. *Arch Pharm Res* **2005**, *28*, 177-179, doi:10.1007/BF02977711.
15. Ren, X.; He, T.; Chang, Y.; Zhao, Y.; Chen, X.; Bai, S.; Wang, L.; Shen, M.; She, G. The Genus Alnus, A Comprehensive Outline of Its Chemical Constituents and Biological Activities. *Molecules* **2017**, *22*, doi:10.3390/molecules22081383.
16. Kang, S.; Kim, J.E.; Li, Y.; Jung, S.K.; Song, N.R.; Thimmegowda, N.R.; Kim, B.Y.; Lee, H.J.; Bode, A.M.; Dong, Z.; et al. Hirsutenone in Alnus extract inhibits akt activity and

suppresses prostate cancer cell proliferation. *Mol Carcinog* **2015**, *54*, 1354-1362, doi:10.1002/mc.22211.

17. Uto, T.; Tung, N.H.; Appiah-Opong, R.; Aning, A.; Morinaga, O.; Edoh, D.; Nyarko, A.K.; Shoyama, Y. Antiproliferative and Pro-Apoptotic Activity of Diarylheptanoids Isolated from the Bark of Alnus japonica in Human Leukemia Cell Lines. *Am J Chin Med* **2015**, *43*, 757-767, doi:10.1142/S0192415X15500470.
18. Sati, S.C.; Sati, N.; Sati, O.P. Bioactive constituents and medicinal importance of genus Alnus. *Pharmacogn Rev* **2011**, *5*, 174-183, doi:10.4103/0973-7847.91115.
19. Kuroyanagi, M.; Shimomae, M.; Nagashima, Y.; Muto, N.; Okuda, T.; Kawahara, N.; Nakane, T.; Sano, T. New diarylheptanoids from Alnus japonica and their antioxidative activity. *Chem Pharm Bull (Tokyo)* **2005**, *53*, 1519-1523, doi:10.1248/cpb.53.1519.
20. Shim, J.K.; Lim, S.H.; Jeong, J.H.; Choi, R.J.; Oh, Y.; Park, J.; Choi, S.; Hong, J.; Kim, S.J.; Moon, J.H.; et al. A lignan from Alnus japonica inhibits glioblastoma tumorspheres by suppression of FOXM1. *Sci Rep* **2022**, *12*, 13990, doi:10.1038/s41598-022-18185-w.
21. Choi, S.E.; Kim, K.H.; Kwon, J.H.; Kim, S.B.; Kim, H.W.; Lee, M.W. Cytotoxic activities of diarylheptanoids from Alnus japonica. *Arch Pharm Res* **2008**, *31*, 1287-1289, doi:10.1007/s12272-001-2108-z.
22. Lee, C.S.; Jang, E.R.; Kim, Y.J.; Myung, S.C.; Kim, W.; Lee, M.W. Diarylheptanoid hirsutenone enhances apoptotic effect of TRAIL on epithelial ovarian carcinoma cell lines via activation of death receptor and mitochondrial pathway. *Invest New Drugs* **2012**, *30*, 548-557, doi:10.1007/s10637-010-9601-5.
23. Liu, N.; Lv, B.; Zeng, Q. Hirsutenone selectively induces cytotoxic effects in human thyroid cancer cells by inhibiting cell migration and invasion, inducing apoptosis and targeting Wnt/beta-catenin signalling pathway. *All Life* **2021**, *14*, 1081-1090, doi:10.1080/26895293.2021.1946432.
24. Leon-Gonzalez, A.J.; Acero, N.; Munoz-Mingarro, D.; Lopez-Lazaro, M.; Martin-Cordero, C. Cytotoxic activity of hirsutanone, a diarylheptanoid isolated from Alnus glutinosa leaves. *Phytomedicine* **2014**, *21*, 866-870, doi:10.1016/j.phymed.2014.01.008.
25. Park, J.Y.; Jeong, H.J.; Kim, J.H.; Kim, Y.M.; Park, S.J.; Kim, D.; Park, K.H.; Lee, W.S.; Ryu, Y.B. Diarylheptanoids from Alnus japonica inhibit papain-like protease of severe acute respiratory syndrome coronavirus. *Biol Pharm Bull* **2012**, *35*, 2036-2042, doi:10.1248/bpb.b12-00623.
26. Tung, N.H.; Kim, S.K.; Ra, J.C.; Zhao, Y.Z.; Sohn, D.H.; Kim, Y.H. Antioxidative and hepatoprotective diarylheptanoids from the bark of Alnus japonica. *Planta Med* **2010**, *76*, 626-629, doi:10.1055/s-0029-1240595.

27. Chi, J.H.; Seo, G.S.; Lee, S.H. Oregonin inhibits inflammation and protects against barrier disruption in intestinal epithelial cells. *Int Immunopharmacol* **2018**, *59*, 134-140, doi:10.1016/j.intimp.2018.04.006.
28. Tung, N.H.; Kwon, H.J.; Kim, J.H.; Ra, J.C.; Ding, Y.; Kim, J.A.; Kim, Y.H. Anti-influenza diarylheptanoids from the bark of Alnus japonica. *Bioorg Med Chem Lett* **2010**, *20*, 1000-1003, doi:10.1016/j.bmcl.2009.12.057.
29. Jamal, Q.M.S. Antiviral Potential of Plants against COVID-19 during Outbreaks-An Update. *Int J Mol Sci* **2022**, *23*, doi:10.3390/ijms232113564.
30. Kuo, K.K.; Lee, K.T.; Chen, K.K.; Yang, Y.H.; Lin, Y.C.; Tsai, M.H.; Wuputra, K.; Lee, Y.L.; Ku, C.C.; Miyoshi, H.; et al. Positive Feedback Loop of OCT4 and c-JUN Expedites Cancer Stemness in Liver Cancer. *Stem Cells* **2016**, *34*, 2613-2624, doi:10.1002/stem.2447.
31. Gushiken, S. Effects of Alnus japonica fermented extract. In *Drinker testimonial materials*; Okinawa Eco-Science Co., Ltd.: **2021**; pp. p1-7.
32. Gushiken, S. *Annual reports on Research Activity in Faculty of Medicine*; University of the Ryukyu, Okinawa, Japan.: **2007.**
33. Hertog, M.G.L.; Hollman, P.C.H.; and Vennema, D.P. Optimization of the Quantitative HPLC Determination of Potentially Anticarcinogenic Flavonoids in Vegetables and Fruits. *Journal of Agricultural and Food Chemistry.* **1992**, 40, 1591-1598. doi.:10.1021/jf00021a023
34. Choi, C.W.; Jung, H.A.; Kang, S.S.; Choi, J.S. Antioxidant constituents and a new triterpenoid glycoside from Flos Lonicerae. *Arch Pharm Res*. **2007**, 30, 1-7. doi: 10.1007/BF02977770.
35. Sun, S.; Liu, M.; He, J.; Li, K.; Zhang, X.; Yin, G. S. Identification and Determination of Seven Phenolic Acids in Brazilian Green Propolis by UPLC-ESI-QTOF-MS and HPLC. *Molecules* **2019**, 24, 1791. doi: 10.3390/molecules24091791.
36. Mosmann, T. Rapid colorimetric assay for cellular growth and survival: application to proliferation and cytotoxicity assays. *J Immunol Methods* **1983**, *65*, 55-63, doi:10.1016/0022-1759(83)90303-4.
37. Strober, W. Trypan Blue Exclusion Test of Cell Viability. *Curr Protoc Immunol* **2015**, *111*, A3 B 1-A3 B 3, doi:10.1002/0471142735.ima03bs111.
38. Claro, F.; Hayes, H.; Cribiu, E.P. The C-, G-, and R-banded karyotype of the scimitar-horned oryx (Oryx dammah). *Hereditas* **1994**, *120*, 1-6, doi:10.1111/j.1601-5223.1994.00001.x.
39. Pan, J.; Nakade, K.; Huang, Y.C.; Zhu, Z.W.; Masuzaki, S.; Hasegawa, H.; Murata, T.; Yoshiki, A.; Yamaguchi, N.; Lee, C.H.; et al. Suppression of cell-cycle progression by Jun dimerization protein-2 (JDP2) involves downregulation of cyclin-A2. *Oncogene* **2010**, *29*, 6245-6256, doi:10.1038/onc.2010.355.

40. Wuputra, K.; Tsai, M.H.; Kato, K.; Ku, C.C.; Pan, J.B.; Yang, Y.H.; Saito, S.; Wu, C.C.; Lin, Y.C.; Cheng, K.H.; et al. Jdp2 is a spatiotemporal transcriptional activator of the AhR via the Nrf2 gene battery. *Inflamm Regen* **2023**, *43*, 42, doi:10.1186/s41232-023-00290-6.
41. Tanigawa, S.; Lee, C.H.; Lin, C.S.; Ku, C.C.; Hasegawa, H.; Qin, S.; Kawahara, A.; Korenori, Y.; Miyamori, K.; Noguchi, M.; et al. Jun dimerization protein 2 is a critical component of the Nrf2/MafK complex regulating the response to ROS homeostasis. *Cell Death Dis* **2013**, *4*, e921, doi:10.1038/cddis.2013.448.
42. Jin, C.; Kato, K,; Chimura, T.; Yamasaki, T.; Nakade, K.; Murata, T.; Li. H.; Pan, J.: Zhao, M.; Sun K.; et al. Regulation of histone acetylation and nucleosome assembly by transcription factor JDP2. *Nat Struct Mol Biol* **2006**, 13, 331-338. doi: 10.1038/nsmb1063. Epub 2006 Mar 5. PMID: 16518400
43. Schneider, C.A.; Rasband, W.S.; Eliceiri, K.W. NIH Image to ImageJ: 25 years of image analysis. *Nat Methods* **2012**, *9*, 671-675, doi:10.1038/nmeth.2089.
44. Schindelin, J.; Arganda-Carreras, I.; Frise, E.; Kaynig, V.; Longair, M.; Pietzsch, T.; Preibisch, S.; Rueden, C.; Saalfeld, S.; Schmid, B.; et al. Fiji: an open-source platform for biological-image analysis. *Nat Methods* **2012**, *9*, 676-682, doi:10.1038/nmeth.2019.
45. Will, R.; Bauer, K.; Kudla, M.; Montero-Vergara, J.; Wiemann, S.; Jendrossek, V.; Pena-Llopis, S.; Vega-Rubin-de-Celis, S. A Dual HiBiT-GFP-LC3 Lentiviral Reporter for Autophagy Flux Assessment. *Methods Mol Biol* **2022**, *2445*, 75-98, doi:10.1007/978-1-0716-2071-7_6.
46. Morishita, H.; Kaizuka, T.; Hama, Y.; Mizushima, N. A new probe to measure autophagic flux in vitro and in vivo. *Autophagy* **2017**, *13*, 757-758, doi:10.1080/15548627.2016.1278094.
47. Mizushima, N.; Murphy, L.O. Autophagy Assays for Biological Discovery and Therapeutic Development. *Trends Biochem Sci* **2020**, *45*, 1080-1093, doi:10.1016/j.tibs.2020.07.006.
48. Mauthe, M.; Orhon, I.; Rocchi, C.; Zhou, X.; Luhr, M.; Hijlkema, K.J.; Coppes, R.P.; Engedal, N.; Mari, M.; Reggiori, F. Chloroquine inhibits autophagic flux by decreasing autophagosome-lysosome fusion. *Autophagy* **2018**, *14*, 1435-1455, doi:10.1080/15548627.2018.1474314.
49. Yoon, C.H.; Kim, M.J.; Kim, R.K.; Lim, E.J.; Choi, K.S.; An, S.; Hwang, S.G.; Kang, S.G.; Suh, Y.; Park, M.J.; et al. c-Jun N-terminal kinase has a pivotal role in the maintenance of self-renewal and tumorigenicity in glioma stem-like cells. *Oncogene* **2012**, *31*, 4655-4666, doi:10.1038/onc.2011.634.
50. An, D.H.; Lee, C.H.; Kwon, Y.; Kim, T.H.; Kim, E.J.; Jung, J.I.; Min, S.; Cheong, E.J.; Kim, S.; Kim, H.K.; et al. Effects of Alnus japonica Hot Water Extract and Oregonin on Muscle Loss and Muscle Atrophy in C2C12 Murine Skeletal Muscle Cells. *Pharmaceuticals (Basel)* **2024**, *17*, doi:10.3390/ph17121661.

51. Liu, Y.; Gong, W.; Yang, Z.Y.; Zhou, X.S.; Gong, C.; Zhang, T.R.; Wei, X.; Ma, D.; Ye, F.; Gao, Q.L. Quercetin induces protective autophagy and apoptosis through ER stress via the p-STAT3/Bcl-2 axis in ovarian cancer. *Apoptosis* **2017**, *22*, 544-557, doi:10.1007/s10495-016-1334-2.
52. Monti, E.; Marras, E.; Prini, P.; Gariboldi, M.B. Luteolin impairs hypoxia adaptation and progression in human breast and colon cancer cells. *Eur J Pharmacol* **2020**, *881*, 173210, doi:10.1016/j.ejphar.2020.173210.
53. Kim, M.J.; Song, Y.R.; Kim, Y.E.; Bae, S.J.; Lee, W.Y.; Bak, S.B.; Kim, Y.W. Kaempferol stimulation of autophagy regulates the ferroptosis under the oxidative stress as mediated with AMP-activated protein kinase. *Free Radic Biol Med* **2023**, *208*, 630-642, doi:10.1016/j.freeradbiomed.2023.09.008.
54. Sharma, N.; Gupta, M.; Anand, P.; Akhter, Y.; Al-Dayan, N.; Majed, H.A.; Biswas, S.; Ali, S.; Sarwat, M. Mechanistic Insight into the Autophagic and Apoptotic Activity of Kaempferol on Liver Cancer Cells. *Onco Targets Ther* **2024**, *17*, 579-601, doi:10.2147/OTT.S460359.
55. Yan, X.; Qi, M.; Li, P.; Zhan, Y.; Shao, H. Apigenin in cancer therapy: anti-cancer effects and mechanisms of action. *Cell Biosci* **2017**, *7*, 50, doi:10.1186/s13578-017-0179-x.
56. Yuan, Y.G.; Wang, Y.H.; Xing, H.H.; Gurunathan, S. Quercetin-mediated synthesis of graphene oxide-silver nanoparticle nanocomposites: a suitable alternative nanotherapy for neuroblastoma. *Int J Nanomedicine* **2017**, *12*, 5819-5839, doi:10.2147/IJN.S140605.
57. Qadeer, A.; Al-Khalaifah, H.; Wajid, A.; Zahoor Khan, M.; Khan, S.; Xie, J.; Jiang, J.; Zhong, K.; Kong, B.; Xia, Z. Therapeutic potential of quercetin in hepatocellular carcinoma: Mechanisms, challenges, and clinical insights. *Journal of Agriculture and Food Research* **2026**, *27*, 102812, doi:https://doi.org/10.1016/j.jafr.2026.102812.
58. Mehta, P.A.; Nelson, A.; Loveless, S.; Lane, A.; Fukuda, T.; Teusink-Cross, A.; Elder, D.; Lagory, D.; Miller, E.; Cancelas, J.A.; et al. Phase 1 study of quercetin, a natural antioxidant for children and young adults with Fanconi anemia. *Blood Adv* **2025**, *9*, 1927-1939, doi:10.1182/bloodadvances.2024015053.
59. Nambiar, A.; Kellogg, D., 3rd; Justice, J.; Goros, M.; Gelfond, J.; Pascual, R.; Hashmi, S.; Masternak, M.; Prata, L.; LeBrasseur, N.; et al. Senolytics dasatinib and quercetin in idiopathic pulmonary fibrosis: results of a phase I, single-blind, single-center, randomized, placebo-controlled pilot trial on feasibility and tolerability. *EBioMedicine* **2023**, *90*, 104481, doi:10.1016/j.ebiom.2023.104481.
60. Gao, W.; Wang, X.; Zhou, Y.; Wang, X.; Yu, Y. Autophagy, ferroptosis, pyroptosis, and necroptosis in tumor immunotherapy. *Signal Transduct Target Ther* **2022**, *7*, 196, doi:10.1038/s41392-022-01046-3.

61. Bialik, S.; Zalckvar, E.; Ber, Y.; Rubinstein, A.D.; Kimchi, A. Systems biology analysis of programmed cell death. *Trends Biochem Sci* **2010**, *35*, 556-564, doi:10.1016/j.tibs.2010.04.008.
62. Elmore, S. Apoptosis: a review of programmed cell death. *Toxicol Pathol* **2007**, *35*, 495-516, doi:10.1080/01926230701320337.
63. Dai, W.; Gao, Q.; Qiu, J.; Yuan, J.; Wu, G.; Shen, G. Quercetin induces apoptosis and enhances 5-FU therapeutic efficacy in hepatocellular carcinoma. *Tumour Biol* **2016**, *37*, 6307-6313, doi:10.1007/s13277-015-4501-0.
64. Lindqvist, L.M.; Frank, D.; McArthur, K.; Dite, T.A.; Lazarou, M.; Oakhill, J.S.; Kile, B.T.; Vaux, D.L. Autophagy induced during apoptosis degrades mitochondria and inhibits type I interferon secretion. *Cell Death & Differentiation* **2018**, *25*, 784-796, doi:10.1038/s41418-017-0017-z.
65. Mintseris, J.; Yu, E.; Harvey, E.P.; Hauseman, Z.J.; Fan, L.; et al. Inhibition of oligomeric BAX by an anti-apoptotic dimer. *Cell* **2025**, *188*, 7397-7412 e7321, doi:10.1016/j.cell.2025.10.037.
66. Moretti, L.; Attia, A.; Kim, K.W.; Lu, B. Crosstalk between Bak/Bax and mTOR signaling regulates radiation-induced autophagy. *Autophagy* **2007**, *3*, 142-144, doi:10.4161/auto.3607.
